# Potential Biomarkers and Signaling Pathways Associated with the Pathogenesis of Primary Ameloblastoma: A Systems Biology Approach

**DOI:** 10.1155/2022/3316313

**Published:** 2022-09-16

**Authors:** Zeynab Bayat, Azin Mirzaeian, Amir Taherkhani

**Affiliations:** ^1^Department of Oral and Maxillofacial Medicine, Faculty of Dentistry, Hamadan University of Medical Sciences, Hamadan, Iran; ^2^Research Center for Molecular Medicine, Hamadan University of Medical Sciences, Hamadan, Iran

## Abstract

**Objective:**

Ameloblastoma is a benign odontogenic tumor that may lead to ameloblastic carcinoma. This study aimed to determine potential signaling pathways and biological processes, critical genes and their regulating transcription factors (TFs), and miRNAs, as well as protein kinases involved in the etiology of primary ameloblastoma.

**Methods:**

The dataset GSE132472 was obtained from the GEO database, and multivariate statistical analyses were applied to identify differentially expressed genes (DEGs) in primary ameloblastoma tissues compared to the corresponding normal gingiva samples. A protein-protein interaction (PPI) map was built using the STRING database. The Cytoscape software identified significant modules and the hub genes within the PPI network. Gene Ontology annotation and signaling pathway analyses were executed by employing the DAVID and Reactome databases, respectively. Significant TFs and miRNAs acting on the hub genes were identified using the iRegulon plugin and MiRWalk 2.0 database, respectively. A protein kinase enrichment analysis was conducted using the online Kinase Enrichment Analysis 2 (KEA2) web server. The approved drugs acting on the hub genes were also found.

**Results:**

A total of 1,629 genes were differentially expressed in primary ameloblastoma (*P* value <0.01 and |Log2FC| > 1). *HRAS*, *CDK1*, *MAPK3*, *ERBB2*, *COL1A1*, *CYCS*, and *BRCA1* demonstrated high degree and betweenness centralities in the PPI network. *E2F4* was the most significant TF acting on the hub genes. *BTK* was the protein kinase significantly enriched by the TFs. Cholesterol biosynthesis was considerably involved in primary ameloblastoma.

**Conclusions:**

This study provides an intuition into the potential mechanisms involved in the etiology of ameloblastoma.

## 1. Introduction

Odontogenic tumors are a class of heterogeneous lesions arising from the tooth-forming tissue, including ectomesenchyme and epithelium remnants associated with the teeth' formation [1, 2]. These tumors could affect individuals of different ages, with peripheral or central tumor locations in the maxillary or mandibular region, leading to facial swelling [3, 4]. Ameloblastoma is an odontogenic tumor originating from the cells close to the tooth-root derived from the ectoderm germ layer. Although ameloblastoma is the pathology in 1% of all tumors in the jaw region, they are known as the second commonest odontogenic tumor. They occur equally in men and women with ages ranging from 30 to 50, with approximately 80% of the cases in the mandible. They are principally benign tumors; however, they show aggressive behavior and could lead to ameloblastic carcinoma (also known as malignant ameloblastoma). The recurrence rate of ameloblastoma is high if the lesions are not cut off perfectly during surgery [5]. Although several types of research have been executed to elucidate molecular changes in odontogenic tumors such as ameloblastoma, their exact underlying mechanisms, including cellular differentiation and tumorigenesis, are unclear and need more studies in this field [6].

Inside human cells, thousands of genes, transcriptomes, and proteins function in various complicated biological networks, including protein-protein interaction (PPI), gene regulatory, metabolic, and signaling networks [7]. Therefore, the conventional gene-by-gene method is not as vigorous to achieve a comprehensive view of cellular action mechanisms. The microarray technique has been developed to simultaneously measure the complete genome's expression in biosystems; this provides scientists an excellent opportunity to disclose signaling pathways and biological processes dysregulated in several human disorders. By using microarray technology, functional genomics is also achievable, unraveling gene functions. Biomarker discovery is another application of this practical approach, leading to identifying disease-specific markers including diagnostic, predictive, and prognostic molecular markers. All these applications could potentially lead to a better diagnosis and medication of the disease. As a high-throughput technique, analyzing the large amount of data generated by microarray technology needs new and vigorous methods to comprehend biological complexity and therapeutic development accurately. To this end, bioinformatics approaches are recommended, which use computational, statistical, and mathematical algorithms to analyze massive biological datasets [8, 9].

In this study, we hypothesized that the significant change in the expression of numerous transcripts in primary ameloblastoma tissues compared to the adjacent healthy gingiva samples leads to an abnormal expression of several proteins, which may lead to the misregulation of miscellaneous pathways and biological processes (BPs) involved in the etiology of ameloblastoma. Moreover, the most critical genes (hub genes), transcription factors (TFs), and protein kinases associated with the pathogenesis of primary ameloblastoma could be illustrated by the protein-protein interaction (PPI) network, gene regulatory network (GRN), and protein kinase enrichment analyses, respectively, therefore, may be assigned as potential biomarkers of primary ameloblastoma.

The present study aimed to identify (1) differentially expressed genes (DEGs) in primary ameloblastoma tissues compared to the corresponding normal gingiva samples, (2) hub genes and clusters in the PPI network associated with the primary ameloblastoma, (3) TFs and protein kinases regulating hub genes and TFs, respectively, (4) the most important signaling pathways and BPs enriched in primary ameloblastoma, and (5) approved drugs for possible inhibition or activating the upregulated and downregulated hub genes, respectively.

## 2. Methods

### 2.1. Microarray Dataset Retrieval

A gene expression dataset developed by Kondo et al. [10] was analyzed to study the mRNA expression alterations in primary ameloblastoma tumors compared to the corresponding normal oral tissue derived from patients with ameloblastoma. The diagnoses were completed by pathologists at Aichi Medical University Hospital, based on the standard histological classification of odontogenic tumors by the World Health Organization (WHO) [11]. The scalpel was used to obtain fresh normal and tumor samples (0.5–2 cm in diameter) from patients for further experiments including cDNA in microarray techniques, real-time RT-PCR, and the western blotting analysis. All the ameloblastoma tumors were located in the mandible.

The total RNA was extracted with DNase treatment using the TRIzolTM reagent (Thermo Fisher Scientific KK) and NucleoSpin RNA (TaKaRa Bio Inc.). The experimental protocol for the cDNA microarray was executed according to the procedure described by Agilent Technologies [12]. Concisely, the Agilent Low Input Quick Amp Labeling Kit (Agilent Technologies) was used for cDNA synthesis and cRNA labeling with the cyanine 3 (Cy3) dye. The Gene Expression Hybridization kit (Agilent Technologies) was used for purification, fragmentation, and hybridization of Cy3-labeled cRNA on a Human Gene Expression 8 × 60 K v2 Microarray Chip containing 26,740 Entrez Gene RNAs. Kondo et al. [10] submitted the normalized and raw microarray data to the GEO database, namely, GSE132472.

The gene expression dataset of GSE132472 [10] was retrieved for subsequent reanalyzing from the Gene Expression Omnibus (GEO), available at https://www.ncbi.nlm.nih.gov/geo/ [13], which is a public access database of microarray profiles. This dataset was based on the GPL16699 platform (Agilent-039494 SurePrint G3 Human GE v2 8 × 60 K Microarray). It consisted of eight primary ameloblastoma tumors, eight normal oral tissue samples, one human ameloblastoma cell line, and one human fibroblast cell line derived from ameloblastoma patients. This project has been reviewed and approved by the Ethical Committee of Hamadan University of Medical Sciences, Hamadan, Iran (Ethics no. IR.UMSHA.REC.1400.599).

### 2.2. Multivariate Statistical Analyses

A new dataset was selected from the GSE132472 TXT file, which consisted of eight ameloblastoma tissue samples and eight normal gingiva tissue specimens collected from patients with primary ameloblastoma. Normalization was executed before statistical analysis, and the unsupervised principal component analysis (PCA) was applied to the dataset to detect outlier sample(s) [14]. Subsequently, the supervised Orthogonal Projections to Latent Structures Discriminant Analysis (OPLS-DA) [14] was used to identify DEGs between early-stage ameloblastoma and healthy tissue samples. All multivariate statistical analyses were performed using the“ropls” package from the R programming environment (version 4.0.0; R Core Team, available at *www*.R-project.org). Genes having an absolute value of Log2 fold change (|Log2 FC|) > 1 and *P* value <0.01 were considered statistically significant.

### 2.3. PPI Network Construction and Identification of Significant Clusters and Hub Genes

The interactions between proteins encoded by the DEGs were identified using the online search tool for the Retrieval of Interacting Genes (STRING) database version 11.5, which is available at https://string-db.org/ [15]. The interaction score cutoff was set to 0.4, and the unconnected nodes were removed from the initial graph. Next, Cytoscape 3.8.0, available at https://www.cytoscape.org [16], was used to visualize the PPI network and execute further structural analyses. Clusters were detected using the Molecular Complex Detection (MCODE) plugin [17], and the modules with the following characteristics were considered significant [18]: MCODE score >3, degree cutoff = 2, node score cutoff = 0.2, *k*-score = 2, and max depth = 100, and the number of nodes ≥10. MCODE is ordinarily used to identify condensed regions in PPI networks, which are assumed to include proteins involved in common signaling pathways and biological processes [19]. The seed node is also detected by the MCODE, which is known as the vertex of each cluster according to its biological function [20]. Furthermore, the network analyzer tool was utilized to calculate the topological features for each node within the PPI network. The hub genes were detected based on their degree and betweenness centralities [19].

### 2.4. Gene Ontology Annotation and Pathway Enrichment Analyses

Gene Ontology (GO) annotation analyses, including biological process (BP), cellular component (CC), and molecular function (MF) enrichment analysis, were carried out by using the Database for Annotation, Visualization and Integrated Discovery (DAVID) database version 6.8, which is available at https://david.ncifcrf.gov/ [21]. Moreover, pathway enrichment analysis was conducted using the Reactome database version 77, available at https://reactome.org/ [22]. Reactome is an open-source and manually curated peer-reviewed pathway database. It provides wise bioinformatics tools to illustrate, interpret, and analyze pathways to support clinical and basic studies, genome analysis, modeling, systems biology, and education [22]. Significant modules were considered for pathway enrichment and BP annotation analysis [18–20, 23]. However, DEGs were used to identify CCs and MFs significantly dysregulated in primary ameloblastoma. The cutoff conditions were set to false discovery rate (FDR) <0.05 and the number of enriched genes ≥2 [23].

### 2.5. GRN Construction

The two master regulators control the expression levels of genes within the cell, including TFs and miRNAs [24]. Previous studies have indicated that TFs and miRNAs can either induce or suppress the expression of their target genes [25, 26]. The binding of miRNA to the promoter can induce the expression of the target gene while binding to 3′ UTR and 5′ UTR, as well as the coding sequence can result in gene silencing [26, 27]. The present study built a GRN consisting of the hub genes, miRNAs, and TFs. The iRegulon plugin was used for the detection of potential TFs for the hub genes [18]. In this regard, the TFs with a normalized enrichment score (NES) >3 were considered statistically significant [28]. Furthermore, the validated upstream miRNAs for the hub genes were identified using the MiRWalk 2.0 database, available at https://zmf.umm.uni-heidelberg.de/apps/zmf/mirwalk2/index.html [29]. Only miRNAs with a number of targets ≥10 were selected for constructing the GRN.

### 2.6. Protein Kinases Enrichment Analysis

Protein kinases and phosphatases play a critical role in posttranslation modification by adding and removing phosphate group to and from proteins. The activity of several essential proteins in the cell depends on the coordination of these two types of enzymes. Therefore, the cell's dysregulation of kinases and phosphatases could cause dysregulation of many crucial signaling pathways and biological processes [30]. Previous studies have linked overexpression of most protein kinases to enhanced cell proliferation, survival, carcinogenesis, metastases, and recurrence of different types of cancer [31–33]. Thus, inhibition of protein kinases has become a promising strategy in cancer therapy, which has already provided satisfying effects [34]. The online Kinase Enrichment Analysis 2 (KEA2) web tool, which is available at https://www.maayanlab.net/KEA2/ [35], was employed to identify protein kinase(s) affecting TFs regulating the hub genes [27]. The KEA2 is being made by the Ma'ayan Lab at the Icahn School of Medicine at Mount Sinai, New York, by manually curating interactions between kinases and their targets in the literature. A list of significant TFs with the criteria of NES >3 was considered as an input list in the KEA2 database.

### 2.7. Identification of Approved Drugs Acting on Hub Proteins

Following the methods of Mahfuz et al. [27], the online DrugBank database (version 5.1.8, released on 2020-01-03; available at https://go.drugbank.com/) [36] was used to identify experimental/investigational approved drugs for inhibiting and activating the proteins encoded by the upregulated and downregulated hub genes in ameloblastoma, respectively. This may accelerate the development of new therapies for ameloblastoma in the future. However, the therapeutic effects of these drugs in ameloblastoma must be studied *in vitro* and *in vivo*. This version of DrugBank provides valuable information about 14,583 drug entries including 2,702 approved small molecules, 1,499 approved biologics (peptides, proteins, vaccines, and allergens), 132 nutraceuticals, and over 6,652 experimental drugs (discovery-phase).

## 3. Results

### 3.1. Identification of Differentially Expressed Genes in Ameloblastoma

**Figure 1:**
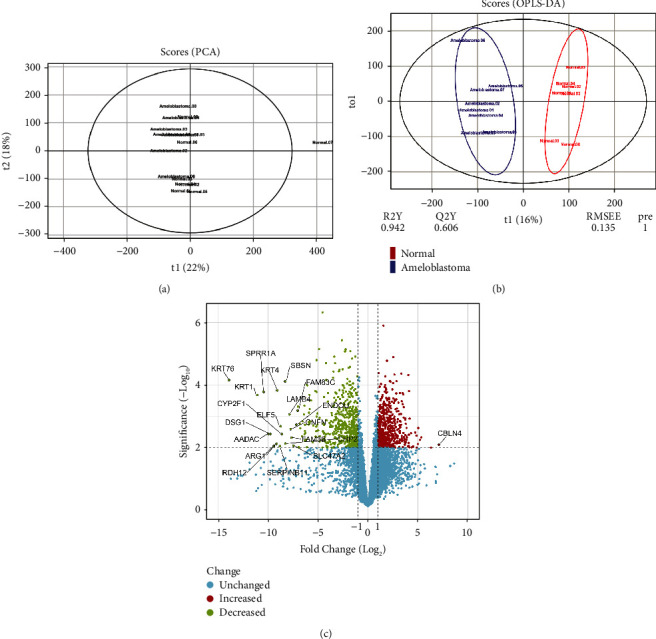
The score plots in the predictive (*x*-axis) and orthogonal (*y*-axis) components of the microarray data of the tissue samples using the (a) PCA and (b) OPLS-DA models. (c) The volcano plot of the genes in primary ameloblastoma compared with normal gingiva. PCA, principal component analysis; OPLS-DA, orthogonal projections to latent structural discriminant analysis.

Following the normalization process, the initial dataset consisted of 16 observations (normal, 8; ameloblastoma, 8) and 58717 variables (probe IDs). According to the PCA plot (*R*^2^*X* = 0.549), one of the observations related to the normal tissue samples was identified as an outlier, and therefore, it was removed before further discriminant analysis (Figure 1(a)). The new dataset consisted of 15 observations and was used for OPLS-DA; this predictive model robustly distinguished primary ameloblastoma from healthy controls (*R*^2^*X* = 0.308, *R*^2^*Y* = 0.942, *Q*^2^ = 0.63) (Figure 1(b)). For multiple probes related to the unique gene, the average value of their expressions was calculated. Finally, a total of 1629 DEGs, including 541 upregulated and 1,088 downregulated genes, with the criteria of *P* value <0.01 and |Log2FC| > 1, were identified for primary ameloblastoma tissue compared with the normal gingiva tissue (Supplementary Table 1).  Figure 1(c) shows the volcano plot demonstrating the overview of DEGs, which was achieved using the Shiny apps web-based tool, available at https://huygens.science.uva.nl/ [37].

### 3.2. PPI Network Analysis

**Figure 2:**
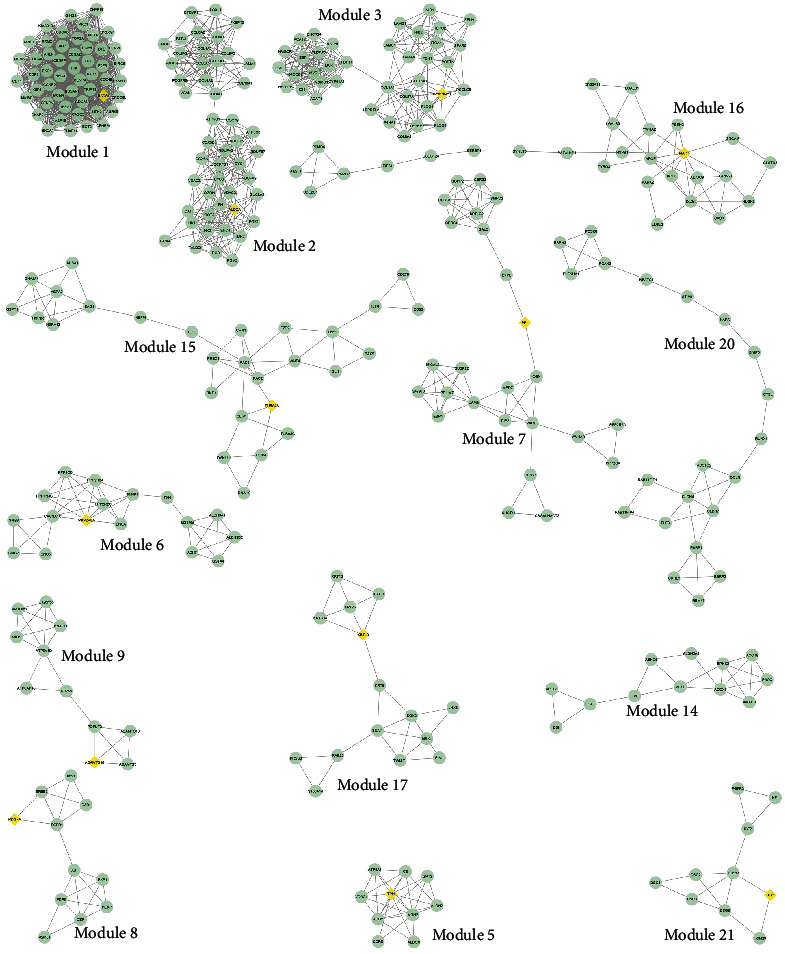
Clustering analysis. These modules were identified using the MCODE plugin in a PPI network based on the DEGs in ameloblastoma compared to normal gingiva. The STRING database was the reference for the identification of interactions between proteins. The yellow circles represent the seed nodes in each cluster. PPI, protein-protein interaction; STRING, search tool for the retrieval of interacting genes; DEG, differentially expressed genes; MCODE, molecular complex detection complex.

**Figure 3:**
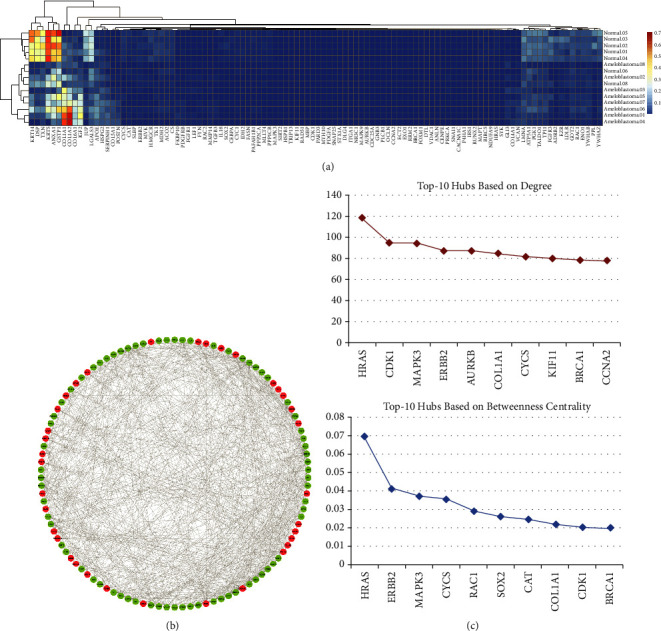
(a) The hierarchical clustering and heat-map of the hub genes. (b) Demonstrating the interaction between hub genes in the PPI network associated with the primary ameloblastoma. The red and green circles represent up- and downregulated genes in ameloblastoma, respectively. (c) Top-10 hub genes based on the degree (red diagram) and betweenness centrality (blue diagram). The *x*-axis and *y*-axis present the name of the gene and their corresponding centrality value, respectively. PPI, protein-protein interaction.

A PPI network was built with a total of 1,500 connected proteins and 9,203 edges. A total of 14 significant clusters were identified within the PPI network using the MCODE plugin (Figure 2). Number 1 was the most considerable condensed region among all modules, with 51 nodes, 1,111 edges, and an MCODE score of 44.44. The characteristics of all modules are presented in Table 1. In addition to the PPI network analysis, the nodes' topological features, including the degree and betweenness centrality of the proteins, were determined using the network analyzer tool. The nodes with the degree and betweenness centrality criteria above the average value of all vertexes within the PPI network were regarded as hub proteins. The average value of the degree and betweenness centrality for the nodes were determined to be 12.27 and 0.00246, respectively. Accordingly, a total of 106 proteins revealed high levels of centrality and were considered hubs (Supplementary Table 2). The heat-map of hub proteins was generated using the R language (version 4.0.2), which is available at https://cran.r-project.org/bin/windows/base/ [38] (Figure 3(a)). Furthermore, the interactions between the hubs were discovered using the STRING knowledge database (Figure 3(b)). The top-10 hubs based on their degree values in the PPI network were as follows: *HRAS*, *CDK1*, *MAPK3*, *ERBB2*, *AURKB*, *COL1A1*, *CYCS*, *KIF11*, *BRCA1*, and *CCNA2*, while *HRAS*, *ERBB2*, *MAPK3*, *CYCS*, *RAC1*, *SOX2*, *CAT*, *COL1A1*, *CDK1*, and *BRCA1* demonstrated the most betweenness centrality value among all genes, respectively (Figure 3(c)).

### 3.3. GO Annotation and Signaling Pathway Analyses

**Figure 4:**
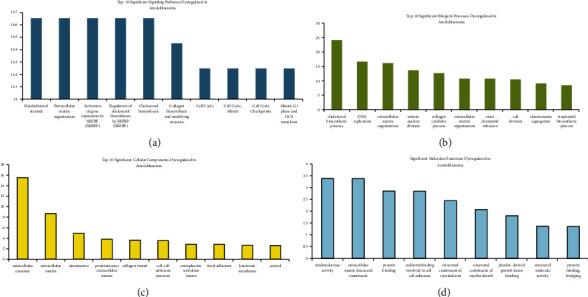
Gene regulatory network. Red and green circles represent up- and downregulated hub genes, respectively. Blue diamonds demonstrate TFs, while yellow rectangles show miRNAs. The violet hexagon illustrates the protein kinase enriched by the TFs. TF, transcription factor.

The Reactome and DAVID knowledge databases revealed that a total of 529 pathways and 78 BPs were significantly affected in ameloblastoma (FDR <0.05). The “metabolism of steroids,” “extracellular matrix organization,” “activation of gene expression by SREBF,” “regulation of cholesterol biosynthesis by SREBF,” “collagen biosynthesis and modifying enzymes,” as well as “cell cycle” were the most significant signaling pathways found to be deregulated in ameloblastoma (Figure 4(a)). Moreover, the most significant BPs deregulated in ameloblastoma were as follows: “cholesterol biosynthesis process,” “DNA replication,” “extracellular matrix organization,” “mitotic nuclear division,” “collagen catabolic process,” and “cell division” (Figure 4(b)). In addition to the results obtained from the DAVID database, it was found that a total of 20CCs and 9MFs were significantly enriched in ameloblastoma. In this regard, “extracellular exosome” (CC), “extracellular matrix” (CC), “desmosome” (CC), “proteinaceous extracellular matrix” (CC), “collagen trimer” (CC), “oxidoreductase activity” (MF), “extracellular matrix structural constituent” (MF), and “protein binding” (MF) demonstrated the most significant FDR (Figures 4(c) and 4(d)). The details of signaling pathways, BPs, and CCs enriched by clusters and DEGs are presented in Supplementary Tables 3–5, respectively.

### 3.4. Identification of Enriched Protein Kinases

By using the KEA2 web server, it was found that the tyrosine-protein kinase (*BTK*) could significantly regulate the general transcription factor II-I (*GTF2I*) at three different phosphorylation sites, including GTF2I_Y248, GTF2I_Y398, and GTF2I_Y503 (adjusted *P* value = 3.84*E* − 07).

### 3.5. Identification of Master Regulators Acting on the Hub Genes

**Figure 5:**
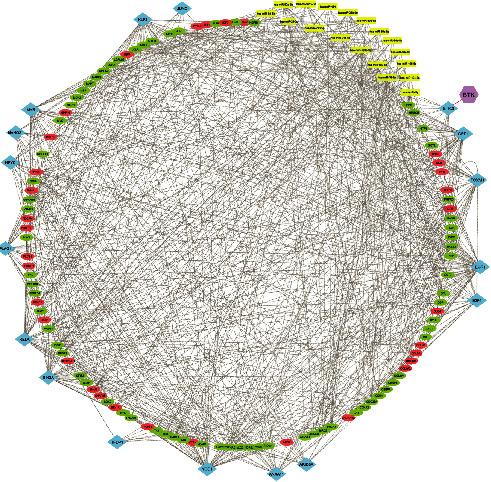
Gene regulatory network. Red and green circles represent up- and downregulated hub genes, respectively. Blue diamonds demonstrate TFs, while yellow rectangles show miRNAs. The violet hexagon illustrates the protein kinase enriched by the TFs. TF, transcription factor.

Master regulators, including TFs and miRNAs, affecting the hub genes were identified, and subsequently, a GRN was built. The TFs with an NES of >3 and miRNAs with a total number of target genes of ≥10 were considered for constructing the GRN. This network included 822 edges and 140 nodes, including 105 hubs, 18 miRNAs, and s17TFs. Of note, the *BTK* was also included in the GRN (Figure 5). The list of target genes related to the TFs and miRNAs is presented in Tables 2 and 3, respectively.

### 3.6. Identification of Approved Drugs

By searching the DrugBank database, a total of 42 and 46 approved drugs (or drug-like compounds) were identified that could act on the upregulated and downregulated hub proteins, respectively. In the case of upregulated proteins, 16 drugs were identified to affect *CACNA1C*, 13 components with *PDGFRB*, five compounds with *NR3C1*, four drugs with *FGFR1*, two compounds with *APOE*, one compound with *MMP14*, one compound with *SNAP25*, one compound with *FYN*, one drug with *PRKCA*, one compound with *P4HA1*, and one compound with *RAC2*. Regarding the downregulated proteins, a total of 33 drugs could act on *ADRB2*, nine components with *ANXA1*, one compound with *MAPK3*, one compound with *NDUFA9*, one drug with *GOT2*, and one compound with *MDH2*.  Table 4 presents the list of identified approved drugs and their associated targets.

## 4. Discussion

Ameloblastoma constitutes 1% of all tumors in the oral cavity, with a prevalence of 0.5 per million individuals each year [39, 40]. Although it is a benign lesion, it could lead to malignant ameloblastoma with a dismal prognosis [41]. This study performed integrated bioinformatics analyses to disclose potential biological procedures, genes, TFs, miRNAs, and protein kinases triggering ameloblastoma.

The OPLS-DA model revealed that several ECM-associated genes including *COL8A1*, *COL8A2*, *COL4A2*, *COL27A1*, *COLEC12*, *COL10A1*, *PCOLCE*, *COLEC11*, *COL6A2*, *COL6A3*, *COL13A*, *COL5A2*, *MMP14*, *COL5A1*, *COL4A1*, *COL1A2*, *COL1A1*, and *COL6A1* were significantly overexpressed in ameloblastoma compared to corresponding normal oral tissue with the criteria of FC >2 and *P* value <0.01. Several previous studies have linked the upregulation of ECM-associated genes and initiation and progression of cancer cells [42–44]. Therefore, overexpression of a considerable number of ECM-associated genes in ameloblastoma may be involved in the formation and development of the disease.

The GO annotation analysis was conducted using the DAVID database to reveal biological processes affected in ameloblastoma. Accordingly, it was found that the most considerable modules in the PPI network were primarily enriched in the cholesterol biosynthesis process, DNA replication, extracellular matrix organization, mitotic nuclear division, and collagen catabolic process. Moreover, the Reactome pathway analysis demonstrated that the most considerable clusters were principally correlated with the metabolism of steroids, extracellular matrix organization, activation of gene expression by *SREBF*, regulation of cholesterol biosynthesis by *SREBF*, and cholesterol biosynthesis, as well as collagen biosynthesis and modifying enzymes. The extracellular matrix term was also associated with the most significant cellular components and molecular functions affected by the DEGs in ameloblastoma. Deregulation of GO annotations and signaling pathways related to the extracellular matrix and collagen biosynthesis in ameloblastoma is expected due to the upregulation of several ECM-related genes in this disease. Therefore, it is suggested that targeting ECM-related genes may result in therapeutic effects in patients with ameloblastoma, although this needs confirmation in the future. Several flavonoids have shown inhibitory effects on *MMP13* and *MMP8* in previous studies [45, 46]. However, the binding affinity of these natural compounds should be examined on other ECM-related genes such as *MMP14*. Of note, the approved drug named Marimastat inhibits *MMP14* [47].

Previous studies have shown that increased uptake and *de novo* biosynthesis of lipids are closely associated with the initiation and progression of cancer [48]. In this regard, it has been reported that glycerophospholipids (GPLs) are involved in several biological processes linked with malignancy, including adhesion, migration, signal transduction, and apoptosis [49]. Moreover, cholesterol plays a role in cellular signal transduction, carcinogenesis signaling pathway, metastasis, and drug resistance [50]. Aparna et al. [51] reported numerous cholesterol clefts in the ameloblastomatous variant of the calcifying odontogenic cyst. It is suggested that the decreased expression and/or activity of lecithin cholesterol acyltransferase in the cyst fluid leads to the accumulation of unesterified cholesterol, which is associated with the formation of cholesterol granuloma in the cyst wall [52].

It may be suggested that hub genes play a significant role in the etiology of ameloblastoma, or that they are dysregulated in response to the tissue's abnormal cellular and molecular changes. In this regard, *HRAS*, *CDK1*, *MAPK3*, *ERBB2*, *COL1A1*, *CYCS*, and *BRCA1* were involved in the top-10 hubs based on degree and betweenness centralities.

The *HRAS* gene encodes GTPase HRas protein [53]. The HRas protein induces the formation of Mst1/Mst2 heterodimers, leading to the deactivation of the Hippo signaling pathway [54]. The present results found that *HRAS* was significantly downregulated in ameloblastoma tissue compared to the corresponding normal area (FC = 0.42; *P*-value = 0.0086). This may be due to the cellular defense mechanism to reduce the formation of Mst1/Mst2 heterodimers, leading to Hippo pathway activation, although this needs confirmation.

Rotellini et al. [55] designed a study to examine the expression of several cancer-related proteins, including cellular tumor antigen p53 (TP53), serine/threonine-protein kinase B-Raf (*BRAF*), and *EGFR*, as well as *ERBB2* in maxillary ameloblastoma. This was executed by using immunohistochemistry and fluorescent in situ hybridization analysis. According to the study results by Rotellini et al. [55], the *ERBB2* protein was negative in all disease samples including primary and metastatic lesions. According to the present results, *ERBB2* was significantly downregulated in ameloblastoma tissue compared to the corresponding normal area (FC = 0.43, *P* value = 0.002).

It has been clarified that cell-cycle dysregulation is a central hallmark of tumor progression [56], in which *CDK1* is one of the primary master regulators involved in the entry of the cell into the M phase. In a study performed by Sonoda et al. [57], no significant change was observed in the *CDK1* expression in ameloblastoma cell line 1(AM-1) cells using polymerase chain reaction (PCR) and western blotting. However, our multivariate analysis found that CDK1 expression was significantly downregulated in ameloblastoma tissue compared to the corresponding healthy controls (FC = 0.33; *P* value = 0.0099).

The mitogen-activated protein kinase 3 is encoded by the *MAPK3* gene. Its overexpression leads to tumor invasion, metastasis, and drug resistance in several carcinomas [58–60]. According to the present results, *MAPK3* was underexpressed in ameloblastoma samples compared to the corresponding healthy controls (FC = 0.33; *P* value = 0.005).

Several previous studies have linked the expression of collagen type I alpha 1(*COL1A1*) and tumorigenesis in different cancers including human gastric cancer [61], lung cancer [62], and ovarian cancer. He et al. [63] reported that *COL1A1* is a tumor promoter gene in OSCC. No specific research was found demonstrating the association between *COL1A1* and ameloblastoma. According to the present results, *COL1A1* was considerably upregulated in ameloblastoma tissues compared to the healthy controls with the criteria of FC = 6.73 and *P* value = 0.00098. Due to the upregulation of several ECM-related genes in ameloblastoma, it may be suggested that *COL1A1* is involved in the tumorigenesis of the disease.

In our previous report, a positive correlation was observed between *CYCS* (cytochrome c protein) overexpression and a dismal prognosis in patients with OSCC; we suggested that this may be due to the enhanced tumorigenesis in OSCC patients with poor prognosis [19]. According to the present results, *CYCS* was downregulated in ameloblastoma tissue compared with the corresponding healthy controls (FC = 0.314; *P* value = 0.004); this may be a part of a physiopathological mechanism involved in the initiation and/or progression of ameloblastoma, although confirmation is inevitable.

The silenced *BRCA1* and/or *BRCA2* have been associated with the deficiency in the DNA double-strand repair and genomic instability, leading to cancer predisposition [64]. Our findings showed significant underexpression in ameloblastoma tissue and corresponding healthy controls (FC = 0.44; *P* value = 0.005).

Accumulating evidence indicates that dysregulation of *E2F* family members could lead to various malignancies, including breast, ovarian, prostate, bladder, colon, and lung adenocarcinoma [65]. According to the present results, *E2F4* was the most significant transcription factor enriched by the hub genes, with an NES of 4.951. Moreover, 28 hub genes were found to be regulated by this gene.

Bruton's tyrosine kinase (BTK) is a critical mediator in the cytoplasm involved in signaling pathways associated with cellular differentiation, proliferation, and immune responses [66]. Recently, Liu et al. [67] demonstrated that *BTK* was considerably overexpressed in the clinical concurrent chemo-radiotherapy-resistant OSCC tissue array and was significantly associated with poor prognosis in OSCC cohorts. Since the exact role of *BTK* in the etiology of ameloblastoma has not been elucidated, further research is required to demonstrate the exact function of the gene in the disease.

Several bioinformatics studies have previously reported the underlying mechanisms of ameloblastoma and potential biomarkers for the disease. Each method and strategy has its advantages, and by integrating all the outcomes from different studies, more reliable results are obtained. Santos et al. [68] performed a valuable bioinformatics analysis to illustrate possible genes involved in ameloblastoma and keratocystic odontogenic tumor (KCOT). In comparison to our study, some of the methods used by Santos et al. [68] were common, and some others were unique. Santos et al. [68] used the GeneCard, available at https://www.genecards.org/ [69] and STRING databases to extract possible genes involved in the pathogenesis of ameloblastoma and to find interactions between the genes, respectively. Furthermore, Santos et al. [68] expanded the PPI network using the STRING database in their study. The authors calculated the sum of the interaction scores of each node in the PPI network and then adjusted the score by multiplying it by 1,000 [70] to achieve a single score named a weighted number of links (WNL). They called the most important genes with the highest WNL “leader genes.” In our study, the PPI network was constructed based on the DEGs obtained from analysis of the microarray technique, which is a high-throughput approach, and our team did not expand the PPI network. Moreover, our study identified the hub genes based on the degree and betweenness of centralities. Furthermore, Santos et al. [68] executed functional enrichment analysis using the STRING database, but we used the DAVID and Reactome databases to perform GO annotation and pathway enrichment analysis, respectively. According to Santos et al. [68] and the present study, CDK1 was considered a critical gene, playing a significant role in the etiology of ameloblastoma. Zhang et al. [71] recently executed an integrated bioinformatics analysis to demonstrate potential biomarkers and molecular mechanisms associated with the epithelial-mesenchymal transition (EMT) and immune infiltration in ameloblastoma. The authors used the CIBERSORT algorithm to study the immune infiltration in ameloblastoma. Zhang et al. [71] reported that a total of 776 genes were deregulated in ameloblastoma, in which *FN1* was upregulated and was linked to the macrophage M2 polarization.

The current study had certain limitations. Only ameloblastoma patients from Aichi Medical University Hospital in Japan were included in the GSE132472 dataset. Therefore, our results may not fully translate to patients in other countries. Moreover, only 16 tissue samples, including eight normal and eight ameloblastoma tissues, were included in the GSE132472 dataset, and therefore, the sample size that we reanalyzed was small. Generating and analyzing more datasets with a more significant number of samples is recommended for future studies. In addition, these findings should be confirmed using molecular experiments such as qRT-PCR, western blotting, and immunofluorescence. Moreover, the absence of subtypes of ameloblastoma samples was another limitation of our study. Analyzing different histological types of tumors may lead to significant results associated with the pathogenesis of ameloblastoma subtypes.

## 5. Conclusion

The present study suggests that a total of 1,629 genes, including 541 up- and 1088 downregulated genes, are differentially expressed in the primary ameloblastoma tissue compared to the corresponding healthy controls. Moreover, 106 hub genes were identified in the PPI network associated with the pathogenesis of the disease. In this regard, *HRAS*, *CDK1*, *MAPK3*, *ERBB2*, *COL1A1*, *CYCS*, and *BRCA1* revealed high levels of centralities based on the degree and betweenness values. Furthermore, 17TFs and 18 miRNAs were also found as master regulators of the hub genes. *E2F4* was the most significant TF, with the NES of 4.951. *BTK* was also found to be significantly enriched by the TFs. The “cholesterol biosynthetic process” and “cholesterol biosynthesis” were demonstrated to be in the top-10 ranked BPs and pathways significantly enriched in ameloblastoma based on their FDR value. Besides, “MAPK1/MAPK3 signaling” (R-HSA-5684996), “MAPK family signaling cascades” (R-HSA-5683057), “MAPK6/MAPK4 signaling” (R-HSA-5687128), and “MAPK cascade” (GO:0000165) were also dysregulated in ameloblastoma (Supplementary Tables 3 and 4). Moreover, translating these results from bioinformatics studies to clinical practice requires more research and concentration on designing new diagnostic kits, which are easy to use in clinical laboratories, and discovering novel therapeutic drugs.

## Figures and Tables

**Table 1:** A total of 14 clusters were detected in the PPI network associated with the primary ameloblastoma.

Cluster no.	MCODE score	No. of nodes	No. of edges	Seed node	Seed degree	Seed betweenness
1	44.44	51	1111	RAD51	75	0.0128
2	12.936	48	304	ALDOA	40	0.0038
3	11.838	38	219	SERPINH1	38	0.0069
5	6.444	10	29	TPI1	64	0.0109
6	4.875	17	39	PPP2R5A	22	0.003
7	4.583	25	55	PPL	28	0.0075
8	4.2	11	21	PDGFA	26	0.0049
9	4	11	20	ADAMTS10	11	0.0018
14	3.818	12	21	NA	NA	NA
15	3.63	28	49	TUBA4A	19	0.0046
16	3.63	28	49	MAPT	49	0.0165
17	3.571	15	25	KRT10	16	0.0025
20	3.2	21	32	NA	NA	NA
21	3.111	10	14	THY1	31	0.0049

PPI, protein-protein interaction.

**Table 2:** A total of 17 transcription factors acting on the hub genes in primary ameloblastoma.

TF	NES	No. of targets	Target genes
E2F4	4.951	28	FASN, NR3C1, TK1, FOXM1, BIRC5, AURKB, SLBP, TRIP13, ANLN, CDC25A, RRM2, HSPB1, CDK1, DLT, EXO1, TPL1, CCNA2, BRCA1, ECT2, KIF11, RAC1, CYCS, RAD51, MAPK3, EZR, MVK, PGK1, FGFR3
MYB	4.555	42	KRT5, PAFAH1B1, SNAP25, FYN, VDAC1, YWHAZ, DLG4, RUNX2, IDH2, IDH2, ENO1, COL5A1, COL1A1, MYH10, ERBB2, SLBP, PPL, SERPINH1, HSPB1, IGF2, YWHAB, TGFB3, HSPG2, MAPK9, EXO1, GLI3, STX1A, MAPK3, JUP, SOX2, GOT2, CDC25A, ANXA1, VCAN, LMNA, PDGFRB, NR3C1, FKBP10, CACNA1C, IRS1, PRKCA, FGFR3, EZR
SIN3A	4.339	48	MVK, NR3C1, ANLN, FGFR1, IRS1, GSTP1, TALDO1, FOXM1, LEF1, RAC1, TGFB3, CYC1, LMNA, MAPK9, DTL, MDH2, FASN, SNAP25, SIRT2, PPL, SYK, RAD51, PDGFA, CYCS, IDH2, GOT2, LDLR, CCNA2, YWHAZ, RRM2, HSPB1, FYN, TRIP13, TK1, AURKB, CDK1, BIRC5, EXO1, KIF11, COL1A1, TPI1, CS, MBP, CDC25A, ENO1, STX1A, SLBP, BRCA1
FOXM1	4.335	34	JUP, IGF2, CDK1, CCNA2, ECT2, KIF11, RAD51, EZR, HRAS, YWHAZ, ANXA1, AURKB, MBP, CDC25A, ANLN, TK1, RRM2, PDGFA, IL18, DTL, LDLR, BIRC5, KRT5, PPL, LMNA, GLI3, KRT14, IDH2, ERBB2, CENPE, FYN, COL1A1, COL6A1
EGR1	4.275	68	CYCS, JUP, COL1A1, DLG4, FKBP10, PDGFRG, IRS1, STX1A, MAPK9, ECT2, PPP2CA, TPI1, ANXA1, CDC25A, CAT, KRT14, HMGCR, GNB3, SNAP25, RUNX2, FGFR1, LMNA, PAFAH1B1, MMP14, MAPK3, LEF1, GLI3, MAPT, NR3C1, CACNA1C, HSPG2, ENO1, SYK, PPL, COL6A1, SOX2, IGF2, TGFB3, ERBB2, FYN, APOE, SERPINH1, VCAN, EZR, PLCB1, OCLN, HSPB1, COL1A2, MYH10, TXN, SNAI1, YWHAZ, FGFR3, P4HA1, MLLT4, YWHAB, CEBPA, PDGFA, ATP5A1, CDK1, MBP, IDH2, COL5A1, PPP1CB, DSP, RAC2, VDAC1, ITGA1
GPD1	4.127	54	TPI1, JUP, COL1A1, PDGFRB, LMNA, STX1A, FGFR3, SERPINH1, HSPG2, IGF2, ACO2, MMP14, PPL, ERBB2, HSPB1, COL5A1, SNAI1, FGFR1, MAPT, APOE, VCAN, SIRT2, KRT5, MBP, GSTP1, NR3C1, GLI3, YWHAZ, FASN, RUNX2, TXN, CACNA1C, LEF1, PAFAH1B1, DLG4, MAPK3, TGFB3, CDC25A, IRS1, KRT14, COL6A1, MAPK9, RAC2, VDAC1, PDGFA, GNB3, SNAP25, FKBP10, SOX2, CEBPA, IDH2, COL1A2, ENO1, FYN
TFDP1	3.901	10	BRCA1, CDK1, EXO1, CDC25A, RRM2, AURKB, TK1, DTL, RAD51, CCNA2
NANOS1	3.794	22	IRS1, YWHAB, PAFAH1B1, SNAP25, NR3C1, YWHAZ, RUNX2, PLCB1, MAPT, FGFR1, CACNA1C, SOX2, FYN, TGFB3, COL1A2, LEF1, GLI3, MAPK9, PPP1CB, ERBB2, MYH10, PDGFRB
KLF4	3.704	43	JUP, ERBB2, ANXA1, LMNA, COL1A2, FGFR3, COL1A1, MAPT, STX1A, HSPB1, FGFR1, HSPG2, DLG4, SNAP25, YWHAZ, PAFAH1B1, TXN, CDK1, LGALS3, IGF2, PPP2CA, PDGFRB, SNAI1, RUNX2, NR3C1, PPL, PGK1, FYN, KRT5, CACNA1C, RAC1, MAPK3, COL5A1, IRS1, SERPINH1, COL4A1, MMP14, ECT2, GLI3, CAT, MYH10, HMGCR, MBP
RELA	3.673	51	CACNA1C, IGF2, JUP, SNAP25, HSPG2, P4HA1, TGFB3, ADRB2, RUNX2, IRS1, YWHAZ, FYN, FGFR1, PGK1, FGFR3, VCAN, COL5A1, SYK, KRT14, VDAC1, LEF1, GLI3, PDGFRB, PPP2CA, CDC25A, DSP, IL18, MYH10, NR3C1, POSTN, IDH2, CENPE, PPP1CB, ECT2, COL4A1, PDGFA, COL6A1, MAPK9, MLLT4, PLCB1, STX1A, ANXA1, ITGA1, PAFAH1B1, EZR, GSTP1, TXN, NDUFA9, LGALS3, YWHAB, RAC1
PLAG1	3.619	37	SOX2, SNAP25, MMP14, DLG4, COL5A1, KRT14, PDGFA, IDH2, FGFR1, LMNA, STX1A, PLCB1, SNAI1, COL1A1, JUP, PAFAH1B1, RUNX2, PDGFRB, IGF2, MAPK9, PPL, IRS1, FYN, LGALS3, MAPT, COL6A1, VCAN, EZR, HSPG2, PPP2CA, ENO1, ADRB2, CACNA1C, NR3C1, SERPINH1, YWHAB, FKBP10
PDLIM5	3.55	27	IRS1, COL1A1, VCAN, LEF1, PAFAH1B1, JUP, PDGFRB, MMP14, COL5A1, STX1A, FYN, IGF2, FGFR1, CACNA1C, NR3C1, LMNA, SNAP25, FKBP10, DLG4, SERPINH1, YWHAZ, ERBB2, MAPT, P4HA1, FGFR3, APOE, PLCB1
UBB	3.519	19	COL1A1, CACNA1C, PDGFRB, JUP, YWHAZ, SOX2, NR3C1, SERPINH1, STX1A, FGFR3,TGFB3, LMNA, COL5A1, KRT5, MMP14, FGFR1, ANXA1, MBP, ADRB2
YOD1	3.244	58	PAFAH1B1, SOX2, FYN, YWHAZ, VCAN, CS, KIF11, GLI3, CDC25A, OCLN, ANXA1, IRS1, DSP, JUP, GOT2, RAC1, ,TGFB3, PLCB1, SNAP25, PPL, RUNX2, STX1A, LEF1, MYH10, ERBB2, CYCS, FGFR1, IDH2, CAT, ITGA1,PDGFRB, YWHAB, MBP, EZR, HSPG2, NR3C1, RRM2, TXN, ATP5A1, MAPK9, PPP2CA, CACNA1C, COL1A1, EXO1, VDAC1, IL18, ACO2, BRCA1, LGALS3, SNAI1, ENO1, FGFR3, PARD3, CDK1, SYK, P4HA1, HMGCR, RAD51
GTF2I	3.241	14	COL1A1, LMNA, JUP, STX1A, PDGFRB, MMP14, CACNA1C, PAFAH1B1, ERBB2, HSPG2, IRS1, SERPINH1, FKBP10, DLG4
NFYA	3.233	19	CDC25A, MYH10, IGF2, IRS1, DLG4, ECT2, YWHAZ, MVK, COL5A1, CDK1, CAT, HMGCR, SOX2, DTL, VCAN, PDGFRB, VDAC1, MAPK9, SLBP
JUND	3.045	7	NDUFA9, PAFAH1B1, PPP2CA, YWHAZ, SNAP25, MYH10, DTL

TF, transcription factor; NES, normalized enrichment score.

**Table 3:** A total of 18 miRNAs acting on the hub genes in primary ameloblastoma.

miRNA ID	No. of targets	Target genes
has-miR-193b-3p	21	BRCA1, CCNA2, CDK1, KIF11, CDC15A, COLA41, CS, ECT2, EXO1, FASN, HMGCR, LEF1, MDH2, PRKCA, RAC2, RAD51, RRM2, TK1, TPI1, TRIP13, YWHAZ
has-miR-124-3p	18	AURKB, COL1A1, ERBB2, CEBPA, COL4A1, FGFR1, JUP, KRT14, LDLR, LMNA, MLLT4, MVK, NR3C1, RAC1, RAD51, RUNX2, SERPINH1, SNAI1
has-miR-92a-3p	18	AURKB, CDK1, ATP5A1, CDC25A, COL4A1, DTL, ENO1, EZR, FASN, GOT2, GSTP1, HMGCR, LDLR, MAPK9, MLLT4, PAFAH1B1, PRKCA, TALDO1
has-miR-335-5p	18	BRCA1, ANXA1, BIRC5, COL6A1, HMGCR, HSPG2, ITGA1, JUP, KRT5, LDLR, MLLT4, MMP14, OCLN, P4HA1, PPL, RUNXA2, SNAI1, STX1A
has-miR-16-5p	15	AURKB, BRCA1, CDK1, CYCS, ATP5A1, BIRC5, CDC25A, COL4A1, DSP, FASN, FGFR1, GOT2, NDUFA9, PAFAH1B1, TPI1
has-miR-26b-5p	15	BRCA1, ANXA1, COL5A1, DTL, ECT2, FGFR3, FOXM1, GNB3, GSTP1, IDH2, SERPINH1, SLBP, SNAI1, STX1A, TK1
has-miR-615-3p	15	HRAS, COL6A1M , CYC1, ENO1, EZR, FASN, HMGCR, JUP, LMNA, MDH2, PPP1CB, SYK, TPI1, VDAC1, YWHAZ
has-miR-93-5p	12	CYCS, BIRC5, FASN, GLI3, IGF2, LDLR, MAPK9, PAFAH1B1, PARD3, RRM2, VDAC1, YWHAZ
has-miR-192-5p	12	BRCA1, CYCS, ANLN, CDC25A, CENPE, DLT, ECT2, MAPK9, PAFAH1B1, PPP1CB, RAD51, TRIP13
has-miR-484	12	BRCA1, BRIC5, CDC25A, ENO1, FASN, GOT2, LDLR, MDH2, RRM2, RUNX2, SERPINH1, YWHAZ
has-miR-30a-5p	11	KIF11, ANXA1, CAT, DTL, JUP, LDLR, RRM2, RUNX2, SNAI1, TPI1, YWHAZ
has-let-7b-5p	11	AURKB, CCNA2, HRAS, BIRC5, CDC25A, CS, DSP, LDLR, OCLN, RRM2, YWHAZ
has-miR-155-5p	11	AURKB, CAT, JUP, NR3C1, PPL, RAC1, RAD51, RRM2, RUNX2, TRIP13, YWHAZ
has-miR-34a-5p	11	KIF11, MAPK3, BIRC5, CDC25A, DSP, LEF1, PDGFRB, RRM2, SNAI1, SOX2, STX1A
has-miR-215-5p	11	BRCA1, CYCS, ANLN, CENPE, DTL, ECT2, MAPK9, PAFAH1B1, PPP1CB, RAD51, TRIP13
has-miR-218-5p	10	BIRC5, COL4A1, FKBP10, HSPG2, LEF1, PARD3, PRKCA, RUNX2, VCAN, YWHAB
has-miR-30c-5p	10	KIF11, BIRC5, ECT2, JUP, LDLR, PLCB1, RAC1, RRM2, RUNX2, SNAI1
has-miR-24-3p	10	AURKB, BRCA1, CCNA2, CDK1, DTL, FGFR3, MMP14, PRKCA, RRM2, YWHAZ

miRNA, microRNA.

**Table 4:** A list of approved drugs in the drugbank database acting on some of the hub genes in primary ameloblastoma.

A, inhibitor/antagonists/blocker (drugbank ID) for upregulated genes	
Target	Drug name
FGFR1	Regorafenib (DB08896), Ponatinib (DB08901), Sorafenib (DB00398), Lenvatinib (DB09078)
MMP14	Marimastat (DB00786)
SNAP25	Botulinum toxin type A (DB00083)
PDGFRB	Imatinib (DB00619), Sorafenib (DB00398), Dasatinib (DB01254), Sunitinib (DB01268), Pazopanib (DB06589), Midostaurin (DB06595), Regorafenib (DB08896), Nintedanib (DB09079), Fostamatinib (DB12010), Pexidartinib (DB12978), Ripretinib (DB14840), Pralsetinib (DB15822), Tivozanib (DB11800)
FYN	Fostamatinib (DB12010)
PRKCA	Midostaurin (DB06595),
P4HA1	Hydralazine (DB01275)
CACNA1C	Nicardipine (DB00622), Isradipine (DB00270), Verapamil (DB00661), Dronedarone (DB04855), Amlodipine (DB00381), Felodipine (DB01023), Nifedipine (DB01115), Nimodipine (DB00393), Nisoldipine (DB00401), Nitrendipine (DB01054), Cinnarizine (DB00568), Nilvadipine (DB06712), Levamlodipine (DB09237), Isavuconazole (DB11633), Propiverine (DB12278), Diltiazem (DB00343)
APOE	Zinc chloride (DB14533), zinc sulfate (DB14548)
NR3C1	Mifepristone (DB00834), Fluoxymesterone (DB01185), Ulipristal (DB08867), Spironolactone (DB00421), Gestrinone (DB11619)
RAC2	Dextromethorphan (DB00514)

B, activator/agonists/cofactor/inducer (drugbank ID) for downregulated genes	
Target	Drug name
NDUFA9	Flavin adenine dinucleotide (DB03147)
GOT2	Pyridoxal phosphate (DB00114)
MDH2	Xanthinol (DB09092)
ANXA1	Hydrocortisone (DB00741), Amcinonide (DB00288), Dexamethasone (DB01234 ), Fluocinolone acetonide (DB00591), Methylprednisolone (DB00959), Clobetasol propionate (DB01013), Betamethasone phosphate (DB14669), Dexamethasone acetate (DB14649), Cortisone acetate (DB01380)
MAPK3	Arsenic trioxide (DB01169)
ADRB2	Orciprenaline (DB00816), Ritodrine (DB00867), Terbutaline (DB00871), Salmeterol (DB00938), Formoterol (DB00983), Salbutamol (DB01001), Epinephrine (DB00668), Pseudoephedrine (DB00852), Pindolol (DB00960), Isoprenaline (DB01064), Arformoterol (DB01274), Procaterol (DB01366), Clenbuterol (DB01407), Fenoterol (DB01288), Pirbuterol (DB01291), Norepinephrine (DB00368), Indacaterol (DB05039), Droxidopa (DB06262), Acebutolol (DB01193), Arbutamine (DB01102), Dobutamine (DB00841), Dipivefrin (DB00449), Bopindolol (DB08807), Isoetharine (DB00221), Phenylpropanolamine (DB00397), Olodaterol (DB09080), Vilanterol (DB09082), Celiprolol (DB04846), Levosalbutamol (DB13139), Protokylol (DB06814), Racepinephrine (DB11124), Etafedrine (DB11587), Ephedrine (DB01364)

## Data Availability

The datasets used and/or analyzed during the current study are available from the corresponding author upon reasonable request.

## Supplementary Materials

Supplementary Materials Supplementary Table 1. A total of 1,629 differentially expressed genes in primary ameloblastoma compared to normal gingiva identified by microarray analysis. Supplementary Table 2. A total of 106 genes in the PPI network considered as hubs. Supplementary Table 3. Signaling pathways significantly deregulated in primary ameloblastoma compared to the normal gingiva. Supplementary Table 4. Biological processes significantly deregulated in primary ameloblastoma compared with the normal gingiva. Supplementary Table 5. Cellular components significantly deregulated in primary ameloblastoma compared to the normal gingiva.
