# Supplementary material for: Potential Biomarkers and Signaling Pathways Associated with the Pathogenesis of Primary Ameloblastoma: A Systems Biology Approach
Source: Int J Dent. 2022 Sep 16;2022:3316313. doi: 10.1155/2022/3316313 (PMC9507750; doi:10.1155/2022/3316313)
Supplement: Supplementary Materials — Supplementary Table 1. A total of 1,629 differentially expressed genes in primary ameloblastoma compared to normal gingiva identified by microarray analysis. Supplementary Table 2. A total of 106 genes in the PPI network considered as hubs. Supplementary Table 3. Signaling pathways significantly deregulated in primary ameloblastoma compared to the normal gingiva. Supplementary Table 4. Biological processes significantly deregulated in primary ameloblastoma compared with the normal gingiva. Supplementary Table 5. Cellular components significantly deregulated in primary ameloblastoma compared to the normal gingiva. [file 3316313.f1.docx]

**Supplementary Tables**

**Supplementary Table 1.** A total of 1629 differentially expressed genes in primary ameloblastoma compared to normal gingiva identified by microarray analysis.

| **A, Upregulated genes** | | | |
| --- | --- | --- | --- |
| **Gene name** | **P-value** | **Log2FC** | **AbsLog2FC** |
| EFEMP2 | 1.23E-05 | 2.479 | 2.479 |
| TBXA2R | 1.89E-05 | 2.365 | 2.365 |
| UGGT2 | 3.67E-05 | 1.452 | 1.452 |
| ZFHX4 | 3.85E-05 | 2.450 | 2.450 |
| P4HA1 | 4.65E-05 | 1.496 | 1.496 |
| PHLDA1 | 6.61E-05 | 2.426 | 2.426 |
| CPXM1 | 7.01E-05 | 3.941 | 3.941 |
| HSPG2 | 7.41E-05 | 1.448 | 1.448 |
| MYH10 | 7.52E-05 | 2.315 | 2.315 |
| KIAA1217 | 8.80E-05 | 1.563 | 1.563 |
| ATP6AP1L | 8.83E-05 | 3.308 | 3.308 |
| WTIP | 1.05E-04 | 1.370 | 1.370 |
| PTPRD | 1.16E-04 | 3.982 | 3.982 |
| FAM19A5 | 1.24E-04 | 2.731 | 2.731 |
| EMILIN2 | 1.27E-04 | 1.998 | 1.998 |
| GLI3 | 1.37E-04 | 1.952 | 1.952 |
| CD200 | 1.39E-04 | 2.112 | 2.112 |
| EVC | 1.42E-04 | 1.502 | 1.502 |
| DENND5A | 1.48E-04 | 1.161 | 1.161 |
| PLEKHG2 | 1.55E-04 | 1.449 | 1.449 |
| FAM59B | 1.59E-04 | 1.964 | 1.964 |
| DCHS1 | 1.92E-04 | 2.396 | 2.396 |
| FCN3 | 1.93E-04 | 2.283 | 2.283 |
| SERPINH1 | 2.01E-04 | 2.366 | 2.366 |
| STX1A | 2.09E-04 | 1.573 | 1.573 |
| PLAT | 2.32E-04 | 2.933 | 2.933 |
| CLIP3 | 2.36E-04 | 2.412 | 2.412 |
| MIAT | 2.37E-04 | 3.209 | 3.209 |
| LEPREL4 | 2.41E-04 | 1.840 | 1.840 |
| SNCAIP | 2.41E-04 | 2.548 | 2.548 |
| PRAF2 | 2.42E-04 | 1.421 | 1.421 |
| MATN3 | 2.43E-04 | 4.205 | 4.205 |
| RAI14 | 2.45E-04 | 2.176 | 2.176 |
| RNF215 | 2.46E-04 | 1.613 | 1.613 |
| TIMP2 | 2.60E-04 | 2.161 | 2.161 |
| GALC | 2.96E-04 | 1.210 | 1.210 |
| CLSTN3 | 3.27E-04 | 1.274 | 1.274 |
| NRP2 | 3.29E-04 | 2.310 | 2.310 |
| TMEM108 | 3.32E-04 | 3.684 | 3.684 |
| FAM87B | 3.39E-04 | 1.816 | 1.816 |
| MMP14 | 3.40E-04 | 2.494 | 2.494 |
| MARVELD1 | 3.40E-04 | 1.798 | 1.798 |
| FYN | 3.48E-04 | 1.878 | 1.878 |
| CERCAM | 3.85E-04 | 2.712 | 2.712 |
| ATF7IP2 | 3.89E-04 | 1.617 | 1.617 |
| ROR2 | 3.99E-04 | 3.053 | 3.053 |
| FAM195B | 4.06E-04 | 1.246 | 1.246 |
| CHRFAM7A | 4.10E-04 | 1.447 | 1.447 |
| CASK | 4.19E-04 | 1.687 | 1.687 |
| CMTM3 | 4.23E-04 | 2.350 | 2.350 |
| KIF7 | 4.46E-04 | 2.258 | 2.258 |
| KDELC1 | 4.49E-04 | 1.649 | 1.649 |
| MGC4294 | 4.49E-04 | 3.177 | 3.177 |
| LEPREL2 | 4.70E-04 | 1.634 | 1.634 |
| ST8SIA4 | 4.72E-04 | 2.143 | 2.143 |
| ADRA1B | 4.73E-04 | 3.133 | 3.133 |
| IQCA1 | 4.79E-04 | 2.105 | 2.105 |
| CRISPLD1 | 4.83E-04 | 3.270 | 3.270 |
| GLIS2 | 4.84E-04 | 3.407 | 3.407 |
| PXDN | 5.18E-04 | 2.542 | 2.542 |
| FZD2 | 5.21E-04 | 2.719 | 2.719 |
| CPNE5 | 5.27E-04 | 1.397 | 1.397 |
| EMILIN1 | 5.27E-04 | 2.683 | 2.683 |
| DPYSL3 | 5.30E-04 | 1.686 | 1.686 |
| COL8A1 | 5.53E-04 | 3.651 | 3.651 |
| MPPED2 | 5.61E-04 | 3.163 | 3.163 |
| SALL4 | 6.14E-04 | 2.815 | 2.815 |
| COL8A2 | 6.16E-04 | 3.172 | 3.172 |
| GXYLT2 | 6.35E-04 | 2.861 | 2.861 |
| STAT2 | 6.43E-04 | 1.102 | 1.102 |
| CLEC11A | 6.85E-04 | 2.601 | 2.601 |
| TSHZ3 | 6.87E-04 | 2.466 | 2.466 |
| NLGN2 | 7.03E-04 | 1.567 | 1.567 |
| SNED1 | 7.14E-04 | 1.568 | 1.568 |
| FGFR1 | 7.28E-04 | 2.902 | 2.902 |
| KLF12 | 7.33E-04 | 1.796 | 1.796 |
| ITGA1 | 7.37E-04 | 3.561 | 3.561 |
| IDUA | 7.38E-04 | 1.351 | 1.351 |
| FSTL1 | 7.44E-04 | 2.548 | 2.548 |
| FGL1 | 7.68E-04 | 4.262 | 4.262 |
| CDC42BPA | 7.79E-04 | 1.377 | 1.377 |
| LEPRE1 | 8.15E-04 | 2.331 | 2.331 |
| NOX4 | 8.16E-04 | 3.467 | 3.467 |
| FADS1 | 8.25E-04 | 1.502 | 1.502 |
| A1BG | 8.31E-04 | 1.565 | 1.565 |
| TMEM2 | 8.54E-04 | 2.326 | 2.326 |
| NALCN | 8.54E-04 | 2.804 | 2.804 |
| ARHGEF40 | 8.68E-04 | 1.984 | 1.984 |
| ST3GAL5 | 8.77E-04 | 1.730 | 1.730 |
| WDR27 | 8.78E-04 | 1.821 | 1.821 |
| PMEPA1 | 9.03E-04 | 2.438 | 2.438 |
| MYO1B | 9.08E-04 | 1.602 | 1.602 |
| TBC1D9 | 9.09E-04 | 2.223 | 2.223 |
| FAM167B | 9.14E-04 | 1.946 | 1.946 |
| ZNF618 | 9.15E-04 | 1.633 | 1.633 |
| PTK7 | 9.31E-04 | 1.369 | 1.369 |
| SYT12 | 9.32E-04 | 1.786 | 1.786 |
| FNDC3B | 9.33E-04 | 1.793 | 1.793 |
| DSEL | 9.35E-04 | 2.606 | 2.606 |
| COL4A2 | 9.39E-04 | 2.701 | 2.701 |
| HEYL | 9.49E-04 | 1.906 | 1.906 |
| PNMA2 | 9.52E-04 | 4.124 | 4.124 |
| CDK14 | 9.62E-04 | 2.686 | 2.686 |
| EDA2R | 9.75E-04 | 2.265 | 2.265 |
| NID1 | 9.84E-04 | 2.793 | 2.793 |
| COL1A1 | 9.90E-04 | 2.750 | 2.750 |
| RCN1 | 1.00E-03 | 1.691 | 1.691 |
| PTPRU | 1.01E-03 | 3.353 | 3.353 |
| PRR5L | 1.01E-03 | 2.601 | 2.601 |
| THY1 | 1.08E-03 | 3.065 | 3.065 |
| DISC1 | 1.09E-03 | 1.734 | 1.734 |
| SEPT11 | 1.14E-03 | 1.891 | 1.891 |
| VCAN | 1.15E-03 | 3.470 | 3.470 |
| TSPAN18 | 1.16E-03 | 2.260 | 2.260 |
| CD276 | 1.16E-03 | 1.947 | 1.947 |
| ZNF469 | 1.16E-03 | 3.341 | 3.341 |
| DAPK1 | 1.19E-03 | 2.004 | 2.004 |
| TIAM2 | 1.23E-03 | 2.324 | 2.324 |
| SNAI1 | 1.24E-03 | 1.596 | 1.596 |
| PNKD | 1.26E-03 | 2.003 | 2.003 |
| STARD13 | 1.27E-03 | 1.955 | 1.955 |
| FHOD3 | 1.27E-03 | 2.995 | 2.995 |
| HMGXB3 | 1.28E-03 | 1.084 | 1.084 |
| MDFI | 1.29E-03 | 1.934 | 1.934 |
| MAGEL2 | 1.30E-03 | 4.145 | 4.145 |
| RAC2 | 1.30E-03 | 1.032 | 1.032 |
| RUNX1 | 1.32E-03 | 2.050 | 2.050 |
| ARHGEF17 | 1.32E-03 | 1.422 | 1.422 |
| PDZRN3 | 1.34E-03 | 1.441 | 1.441 |
| AFAP1 | 1.36E-03 | 1.812 | 1.812 |
| PTHLH | 1.38E-03 | 3.437 | 3.437 |
| TNS3 | 1.38E-03 | 1.789 | 1.789 |
| STRA6 | 1.39E-03 | 4.688 | 4.688 |
| HEPH | 1.41E-03 | 2.978 | 2.978 |
| C17orf82 | 1.41E-03 | 1.422 | 1.422 |
| GLT8D2 | 1.42E-03 | 3.041 | 3.041 |
| DZIP1 | 1.44E-03 | 1.801 | 1.801 |
| CALHM2 | 1.46E-03 | 1.563 | 1.563 |
| APBB2 | 1.48E-03 | 1.383 | 1.383 |
| MRAS | 1.49E-03 | 1.817 | 1.817 |
| CCDC88A | 1.50E-03 | 1.344 | 1.344 |
| MC1R | 1.52E-03 | 1.070 | 1.070 |
| DPY19L1 | 1.52E-03 | 1.347 | 1.347 |
| FLJ10038 | 1.55E-03 | 1.656 | 1.656 |
| VASH1 | 1.55E-03 | 1.714 | 1.714 |
| COL27A1 | 1.56E-03 | 1.943 | 1.943 |
| CACNA1C | 1.59E-03 | 1.432 | 1.432 |
| LRRC3 | 1.59E-03 | 1.137 | 1.137 |
| NAIP | 1.60E-03 | 1.664 | 1.664 |
| MXRA5 | 1.61E-03 | 2.040 | 2.040 |
| PHTF1 | 1.62E-03 | 1.555 | 1.555 |
| RNU11 | 1.63E-03 | 1.289 | 1.289 |
| AXL | 1.64E-03 | 1.557 | 1.557 |
| KIAA1462 | 1.67E-03 | 1.831 | 1.831 |
| AEBP1 | 1.69E-03 | 3.580 | 3.580 |
| SPOCD1 | 1.72E-03 | 4.964 | 4.964 |
| PDLIM7 | 1.72E-03 | 1.564 | 1.564 |
| PGM2L1 | 1.73E-03 | 1.873 | 1.873 |
| SCARF2 | 1.73E-03 | 3.045 | 3.045 |
| AHI1 | 1.74E-03 | 1.380 | 1.380 |
| C11orf95 | 1.75E-03 | 1.430 | 1.430 |
| NAT14 | 1.77E-03 | 1.472 | 1.472 |
| TMEM130 | 1.80E-03 | 2.673 | 2.673 |
| MXRA8 | 1.82E-03 | 3.131 | 3.131 |
| FKBP14 | 1.84E-03 | 1.393 | 1.393 |
| ZNF275 | 1.85E-03 | 1.361 | 1.361 |
| EXTL2 | 1.91E-03 | 1.692 | 1.692 |
| MRC2 | 1.91E-03 | 2.137 | 2.137 |
| CHST11 | 1.91E-03 | 3.621 | 3.621 |
| PBX4 | 1.96E-03 | 1.658 | 1.658 |
| PLXNA3 | 1.97E-03 | 1.147 | 1.147 |
| TRPC1 | 1.97E-03 | 1.550 | 1.550 |
| RHOBTB3 | 1.98E-03 | 2.045 | 2.045 |
| PRSS23 | 2.01E-03 | 2.287 | 2.287 |
| OSBPL7 | 2.04E-03 | 1.421 | 1.421 |
| FAM101B | 2.05E-03 | 2.248 | 2.248 |
| RFX8 | 2.06E-03 | 2.502 | 2.502 |
| CNN2 | 2.07E-03 | 1.199 | 1.199 |
| FBN1 | 2.10E-03 | 3.700 | 3.700 |
| IER5L | 2.10E-03 | 1.447 | 1.447 |
| COL6A1 | 2.11E-03 | 1.940 | 1.940 |
| FAM198B | 2.14E-03 | 2.422 | 2.422 |
| ADAMTS18 | 2.16E-03 | 4.925 | 4.925 |
| PKD2 | 2.17E-03 | 1.071 | 1.071 |
| TNFSF11 | 2.17E-03 | 4.149 | 4.149 |
| APOC1 | 2.22E-03 | 1.939 | 1.939 |
| ARMCX2 | 2.24E-03 | 2.127 | 2.127 |
| PRKCA | 2.25E-03 | 1.795 | 1.795 |
| GJC1 | 2.26E-03 | 2.068 | 2.068 |
| BAI2 | 2.29E-03 | 2.196 | 2.196 |
| MCTP2 | 2.31E-03 | 1.797 | 1.797 |
| PRTFDC1 | 2.31E-03 | 1.609 | 1.609 |
| LTBP1 | 2.31E-03 | 1.718 | 1.718 |
| TPM2 | 2.34E-03 | 1.589 | 1.589 |
| TTC3 | 2.35E-03 | 1.221 | 1.221 |
| FBLN1 | 2.35E-03 | 2.489 | 2.489 |
| TSPAN4 | 2.37E-03 | 1.406 | 1.406 |
| HAPLN1 | 2.37E-03 | 5.441 | 5.441 |
| DAB2 | 2.42E-03 | 2.029 | 2.029 |
| IGFBP5 | 2.43E-03 | 1.952 | 1.952 |
| PLEKHO1 | 2.43E-03 | 1.492 | 1.492 |
| CEP290 | 2.44E-03 | 1.490 | 1.490 |
| TMEM204 | 2.46E-03 | 1.634 | 1.634 |
| NUAK1 | 2.48E-03 | 1.776 | 1.776 |
| PDE4DIP | 2.51E-03 | 1.314 | 1.314 |
| OSBPL3 | 2.53E-03 | 1.220 | 1.220 |
| C20orf195 | 2.54E-03 | 3.083 | 3.083 |
| SPATA20 | 2.54E-03 | 1.120 | 1.120 |
| NACAD | 2.54E-03 | 1.649 | 1.649 |
| CDH11 | 2.59E-03 | 3.836 | 3.836 |
| PDIA5 | 2.61E-03 | 1.013 | 1.013 |
| FBXO16 | 2.61E-03 | 1.614 | 1.614 |
| NID2 | 2.62E-03 | 2.653 | 2.653 |
| ARHGAP22 | 2.62E-03 | 2.060 | 2.060 |
| FKBP10 | 2.67E-03 | 3.354 | 3.354 |
| C17orf57 | 2.69E-03 | 1.846 | 1.846 |
| TGFBI | 2.70E-03 | 1.942 | 1.942 |
| CCDC8 | 2.70E-03 | 2.317 | 2.317 |
| SLC9B2 | 2.72E-03 | 1.305 | 1.305 |
| LRCH2 | 2.73E-03 | 2.334 | 2.334 |
| APOL2 | 2.74E-03 | 1.228 | 1.228 |
| IGFBP7 | 2.74E-03 | 1.649 | 1.649 |
| Q29HP5 | 2.77E-03 | 2.906 | 2.906 |
| TMEM119 | 2.78E-03 | 3.530 | 3.530 |
| GALNTL2 | 2.79E-03 | 3.132 | 3.132 |
| PCDHB11 | 2.79E-03 | 1.497 | 1.497 |
| CTSO | 2.81E-03 | 1.659 | 1.659 |
| F2R | 2.81E-03 | 2.016 | 2.016 |
| ZMYND15 | 2.81E-03 | 1.209 | 1.209 |
| SLC9A5 | 2.83E-03 | 1.739 | 1.739 |
| FKBP7 | 2.84E-03 | 2.040 | 2.040 |
| LOXL3 | 2.85E-03 | 1.728 | 1.728 |
| ZNF415 | 2.87E-03 | 1.480 | 1.480 |
| BEND6 | 2.93E-03 | 2.420 | 2.420 |
| DOCK4 | 2.95E-03 | 1.365 | 1.365 |
| GLIPR1 | 2.96E-03 | 2.270 | 2.270 |
| SUSD1 | 2.98E-03 | 1.662 | 1.662 |
| TRAF3IP3 | 2.99E-03 | 1.397 | 1.397 |
| TMEM121 | 3.00E-03 | 1.742 | 1.742 |
| MAGEH1 | 3.01E-03 | 1.059 | 1.059 |
| GOLIM4 | 3.01E-03 | 1.405 | 1.405 |
| CSGALNACT2 | 3.02E-03 | 1.367 | 1.367 |
| RANBP17 | 3.03E-03 | 2.047 | 2.047 |
| C1QTNF6 | 3.04E-03 | 3.911 | 3.911 |
| FKBP9 | 3.05E-03 | 1.114 | 1.114 |
| DUSP6 | 3.07E-03 | 2.023 | 2.023 |
| FOXF1 | 3.07E-03 | 2.413 | 2.413 |
| INHBA | 3.08E-03 | 3.631 | 3.631 |
| CPXM2 | 3.09E-03 | 1.667 | 1.667 |
| SSBP4 | 3.15E-03 | 1.219 | 1.219 |
| CLMP | 3.16E-03 | 3.229 | 3.229 |
| TPST1 | 3.21E-03 | 2.605 | 2.605 |
| TGFB3 | 3.21E-03 | 2.461 | 2.461 |
| MDK | 3.22E-03 | 2.240 | 2.240 |
| CTSL1 | 3.25E-03 | 1.312 | 1.312 |
| C6orf97 | 3.30E-03 | 2.554 | 2.554 |
| PLOD3 | 3.33E-03 | 1.135 | 1.135 |
| IGSF9B | 3.35E-03 | 1.020 | 1.020 |
| NAP1L3 | 3.37E-03 | 2.507 | 2.507 |
| COLEC12 | 3.38E-03 | 3.050 | 3.050 |
| C1QTNF5 | 3.41E-03 | 2.875 | 2.875 |
| BICD1 | 3.42E-03 | 1.600 | 1.600 |
| ANTXR1 | 3.44E-03 | 1.735 | 1.735 |
| SH3KBP1 | 3.51E-03 | 1.592 | 1.592 |
| TK2 | 3.53E-03 | 1.443 | 1.443 |
| C20orf201 | 3.53E-03 | 1.341 | 1.341 |
| MICAL2 | 3.57E-03 | 1.005 | 1.005 |
| HDGFRP3 | 3.58E-03 | 1.189 | 1.189 |
| PHLDB1 | 3.61E-03 | 1.501 | 1.501 |
| SLC4A3 | 3.63E-03 | 1.965 | 1.965 |
| LAMB1 | 3.64E-03 | 2.714 | 2.714 |
| LSAMP | 3.64E-03 | 2.154 | 2.154 |
| USP11 | 3.64E-03 | 1.122 | 1.122 |
| PDGFRB | 3.66E-03 | 2.153 | 2.153 |
| AKT3 | 3.69E-03 | 1.418 | 1.418 |
| KCTD12 | 3.69E-03 | 1.256 | 1.256 |
| PEAK1 | 3.70E-03 | 1.189 | 1.189 |
| ADAMTS5 | 3.72E-03 | 2.906 | 2.906 |
| SELL | 3.75E-03 | 2.307 | 2.307 |
| ARHGAP25 | 3.78E-03 | 1.351 | 1.351 |
| VPS13C | 3.79E-03 | 1.329 | 1.329 |
| ADC | 3.79E-03 | 3.243 | 3.243 |
| LAMA4 | 3.80E-03 | 1.655 | 1.655 |
| COL10A1 | 3.81E-03 | 5.009 | 5.009 |
| HSPA13 | 3.81E-03 | 1.100 | 1.100 |
| MAP3K12 | 3.81E-03 | 1.571 | 1.571 |
| SNX10 | 3.82E-03 | 1.797 | 1.797 |
| EIF5A2 | 3.85E-03 | 1.228 | 1.228 |
| LAMC1 | 3.85E-03 | 1.165 | 1.165 |
| SPARC | 3.86E-03 | 2.495 | 2.495 |
| HVCN1 | 3.86E-03 | 1.632 | 1.632 |
| IL7R | 3.89E-03 | 3.144 | 3.144 |
| TMSB15B | 3.89E-03 | 1.984 | 1.984 |
| RUNX2 | 3.90E-03 | 2.764 | 2.764 |
| ST3GAL2 | 3.90E-03 | 1.115 | 1.115 |
| SCUBE1 | 3.90E-03 | 1.258 | 1.258 |
| WDR86 | 3.91E-03 | 4.001 | 4.001 |
| GPR171 | 3.92E-03 | 2.267 | 2.267 |
| HHIPL1 | 4.00E-03 | 2.315 | 2.315 |
| PCDH12 | 4.01E-03 | 2.032 | 2.032 |
| CHST12 | 4.02E-03 | 1.009 | 1.009 |
| ISLR | 4.02E-03 | 2.351 | 2.351 |
| PREX1 | 4.09E-03 | 1.598 | 1.598 |
| IKBIP | 4.14E-03 | 1.822 | 1.822 |
| PRAM1 | 4.15E-03 | 1.551 | 1.551 |
| CHN1 | 4.16E-03 | 2.933 | 2.933 |
| TBX2 | 4.17E-03 | 1.474 | 1.474 |
| RNF144A | 4.21E-03 | 1.917 | 1.917 |
| ENOX1 | 4.23E-03 | 2.991 | 2.991 |
| C8orf31 | 4.26E-03 | 2.072 | 2.072 |
| FBXL7 | 4.26E-03 | 1.332 | 1.332 |
| C10orf114 | 4.32E-03 | 2.190 | 2.190 |
| ADAMTS10 | 4.32E-03 | 1.573 | 1.573 |
| NPR2 | 4.36E-03 | 1.197 | 1.197 |
| A1BG-AS1 | 4.37E-03 | 1.415 | 1.415 |
| ITGBL1 | 4.38E-03 | 2.799 | 2.799 |
| VWCE | 4.39E-03 | 2.087 | 2.087 |
| SAP25 | 4.40E-03 | 1.282 | 1.282 |
| LCAT | 4.43E-03 | 1.269 | 1.269 |
| KIF26B | 4.43E-03 | 2.270 | 2.270 |
| DKK3 | 4.45E-03 | 1.690 | 1.690 |
| SLC39A14 | 4.51E-03 | 2.901 | 2.901 |
| PDIA4 | 4.51E-03 | 1.038 | 1.038 |
| FAM92A1 | 4.53E-03 | 1.468 | 1.468 |
| PCDHB10 | 4.54E-03 | 2.457 | 2.457 |
| PCOLCE | 4.55E-03 | 3.664 | 3.664 |
| C21orf96 | 4.56E-03 | 4.161 | 4.161 |
| SULF1 | 4.57E-03 | 3.607 | 3.607 |
| LTBP2 | 4.58E-03 | 1.728 | 1.728 |
| C14orf37 | 4.60E-03 | 1.716 | 1.716 |
| GPX8 | 4.63E-03 | 2.298 | 2.298 |
| CFI | 4.64E-03 | 2.053 | 2.053 |
| POMGNT1 | 4.66E-03 | 1.062 | 1.062 |
| JAM3 | 4.66E-03 | 1.557 | 1.557 |
| EID2B | 4.68E-03 | 2.271 | 2.271 |
| IGF2 | 4.70E-03 | 2.364 | 2.364 |
| CTSK | 4.71E-03 | 3.489 | 3.489 |
| SLC36A4 | 4.74E-03 | 1.137 | 1.137 |
| NDNF | 4.74E-03 | 1.955 | 1.955 |
| ARNT2 | 4.75E-03 | 1.747 | 1.747 |
| TTC17 | 4.77E-03 | 1.299 | 1.299 |
| MAGED4B | 4.78E-03 | 2.174 | 2.174 |
| TWIST1 | 4.80E-03 | 1.740 | 1.740 |
| MAGI2-AS3 | 4.81E-03 | 1.765 | 1.765 |
| COLEC11 | 4.88E-03 | 1.981 | 1.981 |
| CHSY1 | 4.89E-03 | 1.076 | 1.076 |
| STAT4 | 4.91E-03 | 1.735 | 1.735 |
| RGL1 | 4.97E-03 | 1.430 | 1.430 |
| SLC22A17 | 4.98E-03 | 1.563 | 1.563 |
| CLEC2D | 4.98E-03 | 2.200 | 2.200 |
| GNG2 | 4.99E-03 | 2.252 | 2.252 |
| NTM | 4.99E-03 | 2.270 | 2.270 |
| SLC39A13 | 5.00E-03 | 1.242 | 1.242 |
| TGFB1I1 | 5.00E-03 | 1.546 | 1.546 |
| MAP9 | 5.02E-03 | 2.042 | 2.042 |
| LRRC15 | 5.02E-03 | 4.765 | 4.765 |
| CTSZ | 5.02E-03 | 1.509 | 1.509 |
| COL6A2 | 5.03E-03 | 2.236 | 2.236 |
| DLG4 | 5.03E-03 | 1.964 | 1.964 |
| FAT1 | 5.09E-03 | 1.862 | 1.862 |
| UACA | 5.09E-03 | 1.241 | 1.241 |
| POFUT2 | 5.10E-03 | 1.227 | 1.227 |
| L3MBTL3 | 5.11E-03 | 1.254 | 1.254 |
| C3orf18 | 5.18E-03 | 2.125 | 2.125 |
| SLC18A3 | 5.27E-03 | 1.052 | 1.052 |
| RAB34 | 5.31E-03 | 1.160 | 1.160 |
| LAMA1 | 5.35E-03 | 2.770 | 2.770 |
| EMID1 | 5.36E-03 | 2.767 | 2.767 |
| EDIL3 | 5.39E-03 | 3.635 | 3.635 |
| MPP3 | 5.40E-03 | 1.372 | 1.372 |
| RAB23 | 5.44E-03 | 2.081 | 2.081 |
| SLC41A2 | 5.44E-03 | 2.187 | 2.187 |
| PKIG | 5.45E-03 | 1.115 | 1.115 |
| FAM7A1 | 5.48E-03 | 2.007 | 2.007 |
| MSX1 | 5.48E-03 | 2.301 | 2.301 |
| POSTN | 5.57E-03 | 3.228 | 3.228 |
| IRS1 | 5.62E-03 | 2.117 | 2.117 |
| FMOD | 5.64E-03 | 2.218 | 2.218 |
| MME | 5.66E-03 | 3.125 | 3.125 |
| LOXL1 | 5.67E-03 | 1.875 | 1.875 |
| TPBG | 5.69E-03 | 1.340 | 1.340 |
| C1orf216 | 5.72E-03 | 1.525 | 1.525 |
| RORB | 5.73E-03 | 2.028 | 2.028 |
| PPM1K | 5.74E-03 | 1.832 | 1.832 |
| ZNF532 | 5.74E-03 | 1.078 | 1.078 |
| ADAM12 | 5.76E-03 | 4.261 | 4.261 |
| P2RX2 | 5.76E-03 | 1.422 | 1.422 |
| APOL4 | 5.79E-03 | 1.658 | 1.658 |
| FBXL2 | 5.80E-03 | 2.418 | 2.418 |
| WDR90 | 5.81E-03 | 1.008 | 1.008 |
| ANGEL1 | 5.81E-03 | 1.087 | 1.087 |
| ADAMTS3 | 5.82E-03 | 4.779 | 4.779 |
| CD83 | 5.84E-03 | 1.770 | 1.770 |
| STON1 | 5.84E-03 | 2.319 | 2.319 |
| FLRT2 | 5.87E-03 | 3.669 | 3.669 |
| THBS3 | 5.88E-03 | 1.103 | 1.103 |
| FLJ43663 | 5.88E-03 | 1.237 | 1.237 |
| LZTS1 | 5.91E-03 | 2.830 | 2.830 |
| ZNF445 | 5.93E-03 | 1.063 | 1.063 |
| COL5A1 | 5.93E-03 | 3.646 | 3.646 |
| SLIT2 | 5.98E-03 | 2.263 | 2.263 |
| LAYN | 6.09E-03 | 2.463 | 2.463 |
| PCDHB12 | 6.11E-03 | 1.801 | 1.801 |
| TTC28 | 6.17E-03 | 1.130 | 1.130 |
| PRDM6 | 6.21E-03 | 2.722 | 2.722 |
| NF1 | 6.23E-03 | 1.227 | 1.227 |
| PCDH17 | 6.26E-03 | 2.045 | 2.045 |
| MRGPRF | 6.27E-03 | 1.505 | 1.505 |
| COL4A1 | 6.36E-03 | 3.062 | 3.062 |
| MMD | 6.36E-03 | 1.417 | 1.417 |
| PLAU | 6.37E-03 | 2.494 | 2.494 |
| LGALS1 | 6.37E-03 | 1.967 | 1.967 |
| ZFHX3 | 6.37E-03 | 1.149 | 1.149 |
| C2orf27A | 6.38E-03 | 1.355 | 1.355 |
| FHAD1 | 6.39E-03 | 2.670 | 2.670 |
| FYB | 6.43E-03 | 2.198 | 2.198 |
| LARP6 | 6.49E-03 | 1.383 | 1.383 |
| CHRNA7 | 6.50E-03 | 1.432 | 1.432 |
| HTRA3 | 6.50E-03 | 2.220 | 2.220 |
| NR3C1 | 6.51E-03 | 1.348 | 1.348 |
| MKX | 6.55E-03 | 3.805 | 3.805 |
| JAK3 | 6.58E-03 | 1.869 | 1.869 |
| CALU | 6.63E-03 | 1.661 | 1.661 |
| RAB31 | 6.64E-03 | 2.114 | 2.114 |
| SYNPO | 6.68E-03 | 2.573 | 2.573 |
| SLIT3 | 6.69E-03 | 1.565 | 1.565 |
| ST5 | 6.71E-03 | 1.585 | 1.585 |
| FAM165B | 6.77E-03 | 1.522 | 1.522 |
| NRSN2 | 6.77E-03 | 1.361 | 1.361 |
| ZNF664-FAM101A | 6.79E-03 | 2.203 | 2.203 |
| PHF12 | 6.80E-03 | 1.006 | 1.006 |
| GNB3 | 6.80E-03 | 2.023 | 2.023 |
| MPDZ | 6.83E-03 | 1.492 | 1.492 |
| MERTK | 6.83E-03 | 2.839 | 2.839 |
| LEF1 | 6.85E-03 | 2.246 | 2.246 |
| GPX7 | 6.85E-03 | 1.819 | 1.819 |
| COTL1 | 6.88E-03 | 1.579 | 1.579 |
| KGFLP2 | 6.91E-03 | 1.234 | 1.234 |
| B3GAT3 | 6.92E-03 | 1.017 | 1.017 |
| GLS | 6.92E-03 | 1.068 | 1.068 |
| KIAA1644 | 7.12E-03 | 4.017 | 4.017 |
| PARVG | 7.14E-03 | 2.024 | 2.024 |
| SNAP25 | 7.18E-03 | 2.256 | 2.256 |
| COL1A2 | 7.20E-03 | 2.850 | 2.850 |
| PDGFA | 7.22E-03 | 1.267 | 1.267 |
| BMP5 | 7.24E-03 | 4.232 | 4.232 |
| PHLDB2 | 7.32E-03 | 4.398 | 4.398 |
| MEGF11 | 7.32E-03 | 1.540 | 1.540 |
| SH3TC1 | 7.32E-03 | 1.784 | 1.784 |
| COL6A3 | 7.33E-03 | 3.587 | 3.587 |
| FNDC1 | 7.37E-03 | 4.767 | 4.767 |
| RGS5 | 7.38E-03 | 1.376 | 1.376 |
| FAM92A3 | 7.40E-03 | 2.150 | 2.150 |
| CBLN4 | 7.46E-03 | 7.075 | 7.075 |
| ADAM19 | 7.46E-03 | 2.433 | 2.433 |
| VMO1 | 7.50E-03 | 2.556 | 2.556 |
| CEP170 | 7.58E-03 | 1.064 | 1.064 |
| MLLT11 | 7.64E-03 | 1.267 | 1.267 |
| ABTB1 | 7.66E-03 | 1.367 | 1.367 |
| PPEF1 | 7.69E-03 | 2.940 | 2.940 |
| C20orf103 | 7.69E-03 | 2.718 | 2.718 |
| FAM176A | 7.69E-03 | 3.556 | 3.556 |
| ASPN | 7.70E-03 | 4.411 | 4.411 |
| IGF2-AS | 7.73E-03 | 2.485 | 2.485 |
| CCM2 | 7.76E-03 | 1.377 | 1.377 |
| DACT1 | 7.77E-03 | 2.037 | 2.037 |
| C7orf10 | 7.79E-03 | 1.937 | 1.937 |
| NOVA2 | 7.79E-03 | 1.498 | 1.498 |
| MGC4473 | 7.80E-03 | 1.412 | 1.412 |
| RBMS3 | 7.82E-03 | 1.542 | 1.542 |
| PLOD1 | 7.86E-03 | 1.067 | 1.067 |
| DOK3 | 7.86E-03 | 1.474 | 1.474 |
| RHOBTB1 | 7.89E-03 | 2.120 | 2.120 |
| EPHB2 | 7.90E-03 | 3.111 | 3.111 |
| KANK2 | 7.90E-03 | 1.496 | 1.496 |
| WIPF1 | 7.98E-03 | 1.925 | 1.925 |
| MTHFD1L | 7.98E-03 | 1.387 | 1.387 |
| C15orf5 | 8.02E-03 | 1.665 | 1.665 |
| TNFSF12 | 8.09E-03 | 1.146 | 1.146 |
| TSPYL5 | 8.21E-03 | 1.486 | 1.486 |
| SLC2A10 | 8.33E-03 | 1.550 | 1.550 |
| GAA | 8.36E-03 | 1.085 | 1.085 |
| KALRN | 8.38E-03 | 1.523 | 1.523 |
| CMKLR1 | 8.40E-03 | 2.131 | 2.131 |
| HAPLN3 | 8.40E-03 | 1.109 | 1.109 |
| DDR2 | 8.40E-03 | 1.568 | 1.568 |
| PLCB1 | 8.41E-03 | 1.254 | 1.254 |
| CD96 | 8.41E-03 | 1.248 | 1.248 |
| F7 | 8.45E-03 | 1.920 | 1.920 |
| CCDC149 | 8.46E-03 | 1.170 | 1.170 |
| IL18R1 | 8.48E-03 | 1.330 | 1.330 |
| NFATC1 | 8.51E-03 | 1.179 | 1.179 |
| DIRAS3 | 8.52E-03 | 3.319 | 3.319 |
| FAM3C | 8.57E-03 | 1.062 | 1.062 |
| SPRY4 | 8.58E-03 | 3.034 | 3.034 |
| PTPRCAP | 8.59E-03 | 1.648 | 1.648 |
| MEIS3 | 8.60E-03 | 2.257 | 2.257 |
| APOE | 8.72E-03 | 1.376 | 1.376 |
| C5orf62 | 8.72E-03 | 2.786 | 2.786 |
| ADAMTS14 | 8.78E-03 | 2.076 | 2.076 |
| C12orf23 | 8.89E-03 | 1.234 | 1.234 |
| RNF175 | 8.92E-03 | 2.362 | 2.362 |
| C10orf112 | 8.92E-03 | 2.062 | 2.062 |
| CPZ | 8.95E-03 | 2.484 | 2.484 |
| TRIM46 | 9.01E-03 | 2.837 | 2.837 |
| ZNF583 | 9.02E-03 | 1.178 | 1.178 |
| PDPN | 9.08E-03 | 3.170 | 3.170 |
| JAZF1 | 9.13E-03 | 1.450 | 1.450 |
| COL13A1 | 9.13E-03 | 1.908 | 1.908 |
| SDSL | 9.16E-03 | 1.513 | 1.513 |
| PDZRN4 | 9.21E-03 | 3.864 | 3.864 |
| MILR1 | 9.23E-03 | 2.483 | 2.483 |
| TUSC1 | 9.25E-03 | 1.035 | 1.035 |
| HECTD2 | 9.26E-03 | 1.019 | 1.019 |
| EBF4 | 9.27E-03 | 1.389 | 1.389 |
| CRISPLD2 | 9.36E-03 | 2.444 | 2.444 |
| CILP2 | 9.38E-03 | 3.781 | 3.781 |
| ARHGEF25 | 9.38E-03 | 1.559 | 1.559 |
| APLN | 9.39E-03 | 1.858 | 1.858 |
| CNTLN | 9.43E-03 | 1.499 | 1.499 |
| SIRPB1 | 9.45E-03 | 1.331 | 1.331 |
| LRRC17 | 9.45E-03 | 2.811 | 2.811 |
| AMIGO2 | 9.46E-03 | 1.619 | 1.619 |
| QPCTL | 9.53E-03 | 1.290 | 1.290 |
| RASA3 | 9.60E-03 | 1.112 | 1.112 |
| LHX8 | 9.64E-03 | 2.604 | 2.604 |
| PPAPDC1A | 9.69E-03 | 4.772 | 4.772 |
| ENPP1 | 9.69E-03 | 3.217 | 3.217 |
| TUSC3 | 9.71E-03 | 2.441 | 2.441 |
| GUCY1B3 | 9.72E-03 | 1.442 | 1.442 |
| TRAM2 | 9.74E-03 | 1.789 | 1.789 |
| ZNF610 | 9.74E-03 | 2.588 | 2.588 |
| ZKSCAN2 | 9.75E-03 | 1.166 | 1.166 |
| COL5A2 | 9.77E-03 | 3.716 | 3.716 |
| GDF11 | 9.79E-03 | 1.193 | 1.193 |
| MFAP2 | 9.80E-03 | 3.985 | 3.985 |
| DENND5B | 9.82E-03 | 1.174 | 1.174 |
| FLJ31662 | 9.88E-03 | 1.134 | 1.134 |
| CBLN3 | 9.92E-03 | 1.040 | 1.040 |
| PNMAL1 | 9.92E-03 | 3.073 | 3.073 |
| RNF207 | 9.95E-03 | 1.506 | 1.506 |
| **B, Downregulated genes** | | | |
| **Gene name** | **P-value** | **Log2FC** | **AbsLog2FC** |
| CNKSR1 | 5.87E-08 | -4.775 | 4.775 |
| SNAR-A3 | 2.38E-06 | -1.388 | 1.388 |
| SNAR-B2 | 3.03E-06 | -2.061 | 2.061 |
| SNAR-D | 4.07E-06 | -2.031 | 2.031 |
| OR7E24 | 4.78E-06 | -1.809 | 1.809 |
| DCXR | 7.15E-06 | -1.340 | 1.340 |
| OR7E156P | 7.91E-06 | -1.986 | 1.986 |
| C19orf33 | 8.48E-06 | -4.355 | 4.355 |
| NXPH4 | 9.53E-06 | -2.534 | 2.534 |
| SNAR-H | 2.04E-05 | -2.048 | 2.048 |
| SPRR3 | 2.12E-05 | -5.037 | 5.037 |
| CITED4 | 2.21E-05 | -3.506 | 3.506 |
| KLK11 | 2.24E-05 | -5.302 | 5.302 |
| OR7E12P | 2.51E-05 | -2.168 | 2.168 |
| LARP1B | 2.62E-05 | -1.199 | 1.199 |
| MGLL | 2.81E-05 | -2.937 | 2.937 |
| C20orf134 | 3.30E-05 | -1.584 | 1.584 |
| OR7E47P | 3.75E-05 | -1.935 | 1.935 |
| BCL2L2 | 4.43E-05 | -1.042 | 1.042 |
| MT1G | 4.57E-05 | -3.030 | 3.030 |
| CRB3 | 4.75E-05 | -2.635 | 2.635 |
| OR7E14P | 5.00E-05 | -2.017 | 2.017 |
| NFASC | 5.00E-05 | -3.660 | 3.660 |
| IDH2 | 5.79E-05 | -2.329 | 2.329 |
| COBL | 7.35E-05 | -3.658 | 3.658 |
| YIF1B | 8.26E-05 | -3.062 | 3.062 |
| CAB39L | 9.11E-05 | -2.207 | 2.207 |
| CBLC | 9.40E-05 | -4.409 | 4.409 |
| PDCD4 | 9.55E-05 | -1.759 | 1.759 |
| KLK1 | 1.02E-04 | -3.636 | 3.636 |
| SBSN | 1.04E-04 | -8.421 | 8.421 |
| MGST2 | 1.05E-04 | -2.024 | 2.024 |
| CGN | 1.10E-04 | -3.045 | 3.045 |
| DBI | 1.31E-04 | -3.075 | 3.075 |
| HMGCS1 | 1.40E-04 | -2.223 | 2.223 |
| CCDC56 | 1.46E-04 | -1.718 | 1.718 |
| TSTA3 | 1.49E-04 | -1.558 | 1.558 |
| USP54 | 1.53E-04 | -1.409 | 1.409 |
| ELOVL7 | 1.54E-04 | -2.366 | 2.366 |
| RAB3D | 1.55E-04 | -2.709 | 2.709 |
| WDR62 | 1.57E-04 | -3.354 | 3.354 |
| EHF | 1.64E-04 | -2.741 | 2.741 |
| SPRR1B | 1.66E-04 | -6.183 | 6.183 |
| TTC22 | 1.70E-04 | -2.982 | 2.982 |
| SLC25A10 | 1.75E-04 | -2.187 | 2.187 |
| CRNN | 1.84E-04 | -6.260 | 6.260 |
| KRT76 | 1.88E-04 | -13.942 | 13.942 |
| P2RY2 | 1.97E-04 | -2.726 | 2.726 |
| KRT4 | 2.02E-04 | -9.063 | 9.063 |
| HK2 | 2.06E-04 | -1.728 | 1.728 |
| CKMT1A | 2.10E-04 | -3.083 | 3.083 |
| C14orf80 | 2.11E-04 | -1.055 | 1.055 |
| TRIM7 | 2.11E-04 | -3.607 | 3.607 |
| LY6D | 2.21E-04 | -5.265 | 5.265 |
| PDK1 | 2.21E-04 | -1.520 | 1.520 |
| RALGPS1 | 2.23E-04 | -2.552 | 2.552 |
| KPNA4 | 2.26E-04 | -1.141 | 1.141 |
| RHBG | 2.27E-04 | -3.101 | 3.101 |
| EPB41L4A | 2.33E-04 | -1.802 | 1.802 |
| C9orf140 | 2.33E-04 | -2.979 | 2.979 |
| KIF1C | 2.35E-04 | -1.689 | 1.689 |
| SOX15 | 2.47E-04 | -3.452 | 3.452 |
| CLDN7 | 2.53E-04 | -2.226 | 2.226 |
| TACC2 | 2.65E-04 | -2.029 | 2.029 |
| SNAR-G2 | 2.77E-04 | -1.831 | 1.831 |
| CSTB | 2.80E-04 | -3.164 | 3.164 |
| ANO10 | 2.90E-04 | -1.650 | 1.650 |
| MBP | 2.91E-04 | -1.458 | 1.458 |
| CSTA | 2.99E-04 | -4.545 | 4.545 |
| USP2 | 3.03E-04 | -5.294 | 5.294 |
| ESRP1 | 3.04E-04 | -2.646 | 2.646 |
| GPR126 | 3.04E-04 | -3.514 | 3.514 |
| MGC72080 | 3.15E-04 | -1.386 | 1.386 |
| ARHGEF16 | 3.21E-04 | -1.919 | 1.919 |
| MT1H | 3.21E-04 | -4.431 | 4.431 |
| SETD7 | 3.26E-04 | -1.532 | 1.532 |
| TUFT1 | 3.30E-04 | -2.772 | 2.772 |
| NRADDP | 3.30E-04 | -3.600 | 3.600 |
| DNAJA4 | 3.31E-04 | -2.283 | 2.283 |
| KRT1 | 3.38E-04 | -11.147 | 11.147 |
| TNFRSF11A | 3.53E-04 | -2.339 | 2.339 |
| NEBL | 3.63E-04 | -3.335 | 3.335 |
| WNT4 | 3.64E-04 | -2.666 | 2.666 |
| TST | 3.68E-04 | -1.981 | 1.981 |
| FAM83F | 3.68E-04 | -2.360 | 2.360 |
| CS | 3.75E-04 | -1.150 | 1.150 |
| CXCR2 | 3.77E-04 | -3.172 | 3.172 |
| AQP3 | 3.79E-04 | -5.487 | 5.487 |
| B3GALT4 | 3.90E-04 | -1.590 | 1.590 |
| NIPAL2 | 3.99E-04 | -1.809 | 1.809 |
| KIAA0922 | 4.03E-04 | -1.436 | 1.436 |
| GRHL1 | 4.18E-04 | -3.347 | 3.347 |
| FABP6 | 4.18E-04 | -2.602 | 2.602 |
| HS3ST6 | 4.19E-04 | -6.015 | 6.015 |
| ZBTB24 | 4.21E-04 | -1.642 | 1.642 |
| CYB5A | 4.25E-04 | -1.460 | 1.460 |
| KCNK7 | 4.31E-04 | -5.819 | 5.819 |
| PIK3C2B | 4.40E-04 | -1.576 | 1.576 |
| FAM195A | 4.47E-04 | -1.228 | 1.228 |
| NOD2 | 4.49E-04 | -2.417 | 2.417 |
| HYAL1 | 4.54E-04 | -2.142 | 2.142 |
| MAL2 | 4.73E-04 | -3.555 | 3.555 |
| ATL2 | 4.82E-04 | -1.363 | 1.363 |
| INPP5J | 4.82E-04 | -1.823 | 1.823 |
| ENTPD5 | 4.88E-04 | -1.393 | 1.393 |
| NIPAL1 | 4.90E-04 | -2.561 | 2.561 |
| FUT3 | 5.05E-04 | -3.616 | 3.616 |
| IMPA2 | 5.05E-04 | -3.444 | 3.444 |
| KLRG2 | 5.08E-04 | -3.701 | 3.701 |
| PITPNA | 5.15E-04 | -1.935 | 1.935 |
| LEO1 | 5.18E-04 | -1.089 | 1.089 |
| CAMSAP3 | 5.25E-04 | -3.039 | 3.039 |
| GOT2 | 5.28E-04 | -1.129 | 1.129 |
| IDI1 | 5.28E-04 | -1.996 | 1.996 |
| LLGL2 | 5.30E-04 | -2.324 | 2.324 |
| TEX101 | 5.30E-04 | -4.255 | 4.255 |
| TTC39A | 5.49E-04 | -4.726 | 4.726 |
| SLCO4A1 | 5.52E-04 | -3.570 | 3.570 |
| CEBPG | 5.53E-04 | -1.305 | 1.305 |
| MAGIX | 5.54E-04 | -2.124 | 2.124 |
| ACAT2 | 5.59E-04 | -1.742 | 1.742 |
| E2F2 | 5.62E-04 | -2.370 | 2.370 |
| SPRR1A | 5.73E-04 | -7.478 | 7.478 |
| PGLYRP3 | 5.74E-04 | -5.864 | 5.864 |
| GPSM2 | 5.82E-04 | -3.140 | 3.140 |
| SPTBN2 | 5.84E-04 | -2.016 | 2.016 |
| LGALS3 | 5.92E-04 | -1.406 | 1.406 |
| MRPL23 | 5.95E-04 | -1.072 | 1.072 |
| IRX4 | 5.98E-04 | -2.719 | 2.719 |
| RFX2 | 6.07E-04 | -1.592 | 1.592 |
| EPHX2 | 6.10E-04 | -1.732 | 1.732 |
| GRHL3 | 6.37E-04 | -6.529 | 6.529 |
| ESRP2 | 6.41E-04 | -2.224 | 2.224 |
| ALDH3A2 | 6.50E-04 | -2.707 | 2.707 |
| AACS | 6.52E-04 | -1.477 | 1.477 |
| PDS5A | 6.58E-04 | -1.356 | 1.356 |
| ABHD5 | 6.61E-04 | -2.298 | 2.298 |
| TXN | 6.72E-04 | -2.380 | 2.380 |
| CAPNS2 | 6.77E-04 | -5.934 | 5.934 |
| FUT1 | 6.86E-04 | -2.111 | 2.111 |
| ELL3 | 6.93E-04 | -2.486 | 2.486 |
| PI4K2B | 6.98E-04 | -1.423 | 1.423 |
| FGFBP1 | 7.01E-04 | -7.251 | 7.251 |
| CA12 | 7.11E-04 | -2.029 | 2.029 |
| XK | 7.20E-04 | -1.698 | 1.698 |
| DUSP23 | 7.23E-04 | -1.051 | 1.051 |
| PHF19 | 7.31E-04 | -1.368 | 1.368 |
| SLC7A1 | 7.37E-04 | -1.939 | 1.939 |
| C9orf125 | 7.43E-04 | -1.401 | 1.401 |
| ZBTB7B | 7.43E-04 | -2.060 | 2.060 |
| HSPB8 | 7.44E-04 | -5.252 | 5.252 |
| ABLIM1 | 7.47E-04 | -2.853 | 2.853 |
| GPX3 | 7.52E-04 | -2.772 | 2.772 |
| SYTL4 | 7.54E-04 | -2.090 | 2.090 |
| KANK1 | 7.75E-04 | -2.284 | 2.284 |
| PACSIN3 | 7.80E-04 | -1.660 | 1.660 |
| HEBP2 | 7.86E-04 | -1.601 | 1.601 |
| DUSP22 | 7.86E-04 | -1.697 | 1.697 |
| GCH1 | 7.97E-04 | -1.426 | 1.426 |
| BARD1 | 8.13E-04 | -1.763 | 1.763 |
| KIAA0101 | 8.17E-04 | -2.053 | 2.053 |
| SERPINB1 | 8.29E-04 | -2.930 | 2.930 |
| H19 | 8.31E-04 | -4.058 | 4.058 |
| PIR | 8.34E-04 | -2.776 | 2.776 |
| HOOK1 | 8.37E-04 | -2.023 | 2.023 |
| PRSS8 | 8.37E-04 | -1.703 | 1.703 |
| BLVRB | 8.47E-04 | -1.621 | 1.621 |
| CLDN4 | 8.49E-04 | -3.358 | 3.358 |
| CCDC85C | 8.51E-04 | -1.863 | 1.863 |
| XPA | 8.55E-04 | -1.080 | 1.080 |
| C1orf210 | 8.56E-04 | -2.737 | 2.737 |
| CHD7 | 8.62E-04 | -1.435 | 1.435 |
| FAAH2 | 8.75E-04 | -2.161 | 2.161 |
| TPRN | 8.78E-04 | -1.786 | 1.786 |
| NUDT8 | 8.89E-04 | -2.804 | 2.804 |
| PLLP | 9.03E-04 | -1.740 | 1.740 |
| LYNX1 | 9.17E-04 | -1.357 | 1.357 |
| CDCA8 | 9.18E-04 | -1.858 | 1.858 |
| SETD8 | 9.18E-04 | -1.375 | 1.375 |
| KIAA1598 | 9.23E-04 | -1.018 | 1.018 |
| CLIP4 | 9.24E-04 | -1.944 | 1.944 |
| CCDC64B | 9.24E-04 | -3.375 | 3.375 |
| CERS4 | 9.27E-04 | -2.029 | 2.029 |
| MAPK13 | 9.27E-04 | -3.081 | 3.081 |
| CASZ1 | 9.42E-04 | -1.740 | 1.740 |
| DSP | 9.45E-04 | -2.701 | 2.701 |
| ZNF750 | 9.46E-04 | -3.255 | 3.255 |
| ARHGEF37 | 9.46E-04 | -1.992 | 1.992 |
| TMEM11 | 9.48E-04 | -1.113 | 1.113 |
| SHMT1 | 9.75E-04 | -2.238 | 2.238 |
| FAM83C | 9.77E-04 | -7.185 | 7.185 |
| PPP2R3A | 9.85E-04 | -1.782 | 1.782 |
| LGALS7 | 9.87E-04 | -3.847 | 3.847 |
| ATP5S | 9.92E-04 | -1.096 | 1.096 |
| ARSG | 1.00E-03 | -1.928 | 1.928 |
| EVPL | 1.00E-03 | -2.498 | 2.498 |
| PAQR5 | 1.01E-03 | -3.087 | 3.087 |
| DSC2 | 1.02E-03 | -3.765 | 3.765 |
| FGD2 | 1.03E-03 | -1.851 | 1.851 |
| NDUFA4L2 | 1.04E-03 | -3.465 | 3.465 |
| EPB41L5 | 1.04E-03 | -1.665 | 1.665 |
| LSS | 1.04E-03 | -1.094 | 1.094 |
| PCSK6 | 1.05E-03 | -2.992 | 2.992 |
| SLC25A25 | 1.06E-03 | -2.662 | 2.662 |
| SLC39A11 | 1.06E-03 | -1.264 | 1.264 |
| MKNK2 | 1.06E-03 | -1.902 | 1.902 |
| STK40 | 1.07E-03 | -1.483 | 1.483 |
| C19orf57 | 1.09E-03 | -2.091 | 2.091 |
| CYB5R1 | 1.09E-03 | -1.225 | 1.225 |
| TLCD1 | 1.09E-03 | -1.798 | 1.798 |
| ADAM15 | 1.10E-03 | -1.395 | 1.395 |
| C17orf109 | 1.11E-03 | -4.318 | 4.318 |
| EPB41L1 | 1.12E-03 | -1.280 | 1.280 |
| STIM1 | 1.12E-03 | -1.201 | 1.201 |
| ALDOA | 1.13E-03 | -1.380 | 1.380 |
| CBR3 | 1.14E-03 | -2.496 | 2.496 |
| C8orf55 | 1.14E-03 | -1.501 | 1.501 |
| NR2F6 | 1.14E-03 | -1.140 | 1.140 |
| NUAK2 | 1.14E-03 | -6.089 | 6.089 |
| ARHGEF26 | 1.14E-03 | -1.862 | 1.862 |
| SOX21 | 1.16E-03 | -6.598 | 6.598 |
| BLNK | 1.17E-03 | -2.819 | 2.819 |
| MUC15 | 1.18E-03 | -4.848 | 4.848 |
| TRIP13 | 1.18E-03 | -1.363 | 1.363 |
| CARD14 | 1.18E-03 | -2.769 | 2.769 |
| YWHAB | 1.19E-03 | -1.015 | 1.015 |
| ATP13A4 | 1.19E-03 | -5.366 | 5.366 |
| FASN | 1.19E-03 | -1.344 | 1.344 |
| LAD1 | 1.21E-03 | -3.151 | 3.151 |
| TTC7A | 1.22E-03 | -1.124 | 1.124 |
| TMEM45B | 1.22E-03 | -3.596 | 3.596 |
| NEAT1 | 1.23E-03 | -1.801 | 1.801 |
| CDT1 | 1.23E-03 | -1.772 | 1.772 |
| GATM | 1.24E-03 | -2.589 | 2.589 |
| RAPGEFL1 | 1.24E-03 | -3.369 | 3.369 |
| RAB38 | 1.25E-03 | -3.671 | 3.671 |
| TSPO | 1.25E-03 | -1.369 | 1.369 |
| CRYBG3 | 1.25E-03 | -1.201 | 1.201 |
| FAM115C | 1.25E-03 | -2.209 | 2.209 |
| LETM1 | 1.25E-03 | -1.357 | 1.357 |
| FRMD4B | 1.26E-03 | -1.188 | 1.188 |
| LYPD6B | 1.27E-03 | -2.990 | 2.990 |
| CLIP1 | 1.27E-03 | -1.663 | 1.663 |
| RPP40 | 1.28E-03 | -1.339 | 1.339 |
| MAST4-AS1 | 1.28E-03 | -2.414 | 2.414 |
| SQRDL | 1.29E-03 | -2.013 | 2.013 |
| P39194 | 1.29E-03 | -6.889 | 6.889 |
| CMAS | 1.30E-03 | -1.485 | 1.485 |
| TNNT2 | 1.30E-03 | -2.031 | 2.031 |
| ARHGEF10L | 1.31E-03 | -1.045 | 1.045 |
| DLL1 | 1.32E-03 | -1.483 | 1.483 |
| SPTLC2 | 1.32E-03 | -1.020 | 1.020 |
| STRA13 | 1.32E-03 | -1.023 | 1.023 |
| DDX28 | 1.33E-03 | -1.188 | 1.188 |
| NUP98 | 1.34E-03 | -1.189 | 1.189 |
| TRIM16L | 1.35E-03 | -3.143 | 3.143 |
| CCDC153 | 1.35E-03 | -1.203 | 1.203 |
| ANKRD33B | 1.37E-03 | -2.373 | 2.373 |
| GGCT | 1.38E-03 | -1.125 | 1.125 |
| COQ2 | 1.39E-03 | -1.681 | 1.681 |
| TM7SF2 | 1.41E-03 | -4.203 | 4.203 |
| SPINK5 | 1.42E-03 | -4.916 | 4.916 |
| PITX1 | 1.42E-03 | -2.666 | 2.666 |
| TPPP | 1.42E-03 | -3.455 | 3.455 |
| DIAPH1 | 1.43E-03 | -1.531 | 1.531 |
| ERBB3 | 1.43E-03 | -2.043 | 2.043 |
| CYP51A1 | 1.47E-03 | -1.321 | 1.321 |
| CENPM | 1.48E-03 | -1.429 | 1.429 |
| RRAGD | 1.49E-03 | -3.034 | 3.034 |
| PCDH1 | 1.49E-03 | -2.722 | 2.722 |
| PGD | 1.49E-03 | -2.689 | 2.689 |
| HPDL | 1.50E-03 | -2.082 | 2.082 |
| COX8A | 1.50E-03 | -1.123 | 1.123 |
| RAB4A | 1.52E-03 | -1.557 | 1.557 |
| PKMYT1 | 1.52E-03 | -1.419 | 1.419 |
| KLB | 1.52E-03 | -5.959 | 5.959 |
| ACPP | 1.52E-03 | -3.616 | 3.616 |
| ZNF385A | 1.53E-03 | -1.474 | 1.474 |
| FKBP4 | 1.54E-03 | -1.126 | 1.126 |
| C10orf99 | 1.55E-03 | -6.277 | 6.277 |
| TK1 | 1.55E-03 | -1.838 | 1.838 |
| SORT1 | 1.55E-03 | -2.069 | 2.069 |
| EMP1 | 1.55E-03 | -2.206 | 2.206 |
| FLJ35390 | 1.57E-03 | -1.470 | 1.470 |
| DSC3 | 1.57E-03 | -3.380 | 3.380 |
| GSTA4 | 1.57E-03 | -2.471 | 2.471 |
| RCAN3 | 1.58E-03 | -1.246 | 1.246 |
| ERI2 | 1.59E-03 | -1.261 | 1.261 |
| PKP1 | 1.60E-03 | -3.451 | 3.451 |
| SLC1A4 | 1.60E-03 | -2.072 | 2.072 |
| SNRPA1 | 1.61E-03 | -1.167 | 1.167 |
| SPRR2C | 1.61E-03 | -5.453 | 5.453 |
| MAL | 1.63E-03 | -6.592 | 6.592 |
| ANKRD37 | 1.63E-03 | -2.490 | 2.490 |
| CD9 | 1.63E-03 | -1.624 | 1.624 |
| DOT1L | 1.64E-03 | -1.070 | 1.070 |
| LAMB4 | 1.64E-03 | -6.952 | 6.952 |
| SPRR2A | 1.65E-03 | -5.983 | 5.983 |
| SDR16C5 | 1.65E-03 | -4.209 | 4.209 |
| SYNGR1 | 1.66E-03 | -1.764 | 1.764 |
| HRASLS | 1.66E-03 | -3.664 | 3.664 |
| ARRDC1 | 1.67E-03 | -1.011 | 1.011 |
| MAX | 1.68E-03 | -2.152 | 2.152 |
| EPGN | 1.68E-03 | -2.771 | 2.771 |
| SOX21-AS1 | 1.69E-03 | -6.405 | 6.405 |
| SNAR-F | 1.70E-03 | -1.869 | 1.869 |
| TMEM40 | 1.71E-03 | -3.756 | 3.756 |
| SLC35C1 | 1.72E-03 | -1.245 | 1.245 |
| COMTD1 | 1.72E-03 | -1.557 | 1.557 |
| CYP2C9 | 1.72E-03 | -3.342 | 3.342 |
| ETV2 | 1.73E-03 | -1.035 | 1.035 |
| ELF5 | 1.73E-03 | -9.011 | 9.011 |
| C1orf135 | 1.76E-03 | -1.960 | 1.960 |
| FAM46B | 1.77E-03 | -5.357 | 5.357 |
| DAPP1 | 1.77E-03 | -2.809 | 2.809 |
| AHDC1 | 1.77E-03 | -1.222 | 1.222 |
| MLLT4 | 1.78E-03 | -1.282 | 1.282 |
| C11orf80 | 1.78E-03 | -1.582 | 1.582 |
| EMP2 | 1.78E-03 | -1.659 | 1.659 |
| MTM1 | 1.79E-03 | -1.322 | 1.322 |
| MLANA | 1.79E-03 | -5.750 | 5.750 |
| ADH7 | 1.79E-03 | -6.299 | 6.299 |
| PER2 | 1.80E-03 | -2.054 | 2.054 |
| COX7B | 1.81E-03 | -1.317 | 1.317 |
| PARD3 | 1.82E-03 | -1.546 | 1.546 |
| ZHX1 | 1.82E-03 | -1.451 | 1.451 |
| FDFT1 | 1.82E-03 | -1.421 | 1.421 |
| MYO19 | 1.83E-03 | -1.141 | 1.141 |
| RAD51 | 1.83E-03 | -1.603 | 1.603 |
| TMEM125 | 1.85E-03 | -2.916 | 2.916 |
| PSMD7 | 1.85E-03 | -1.032 | 1.032 |
| TUBB4B | 1.85E-03 | -1.988 | 1.988 |
| BDH1 | 1.85E-03 | -2.296 | 2.296 |
| ALDH3A1 | 1.87E-03 | -7.368 | 7.368 |
| ENSA | 1.88E-03 | -1.877 | 1.877 |
| PEX3 | 1.89E-03 | -1.314 | 1.314 |
| MINA | 1.89E-03 | -1.488 | 1.488 |
| JUP | 1.89E-03 | -2.523 | 2.523 |
| OGFRL1 | 1.89E-03 | -1.597 | 1.597 |
| MAP7 | 1.90E-03 | -1.519 | 1.519 |
| ZNF57 | 1.90E-03 | -3.028 | 3.028 |
| AHNAK | 1.90E-03 | -2.692 | 2.692 |
| TFAP2C | 1.90E-03 | -1.657 | 1.657 |
| ABCA5 | 1.92E-03 | -1.038 | 1.038 |
| MIOS | 1.94E-03 | -1.361 | 1.361 |
| KLF8 | 1.95E-03 | -1.714 | 1.714 |
| FAM160A1 | 1.95E-03 | -1.619 | 1.619 |
| TPRG1 | 1.95E-03 | -4.678 | 4.678 |
| GIPC1 | 1.96E-03 | -1.779 | 1.779 |
| CEBPA | 1.96E-03 | -3.118 | 3.118 |
| TJP2 | 1.98E-03 | -1.775 | 1.775 |
| ENDOU | 1.98E-03 | -7.174 | 7.174 |
| NDUFA9 | 1.99E-03 | -1.040 | 1.040 |
| SCNN1A | 2.00E-03 | -3.372 | 3.372 |
| SLC16A6 | 2.01E-03 | -3.779 | 3.779 |
| TOM1L2 | 2.01E-03 | -1.300 | 1.300 |
| MPP7 | 2.01E-03 | -2.642 | 2.642 |
| F12 | 2.01E-03 | -2.339 | 2.339 |
| OR7E37P | 2.02E-03 | -2.002 | 2.002 |
| BRCC3 | 2.02E-03 | -1.186 | 1.186 |
| RGS14 | 2.03E-03 | -1.772 | 1.772 |
| EIF6 | 2.03E-03 | -1.525 | 1.525 |
| HIST1H2AG | 2.03E-03 | -1.557 | 1.557 |
| GALK1 | 2.06E-03 | -1.060 | 1.060 |
| TPRXL | 2.06E-03 | -3.260 | 3.260 |
| PPDPF | 2.06E-03 | -1.254 | 1.254 |
| PIM1 | 2.07E-03 | -3.467 | 3.467 |
| SPRR2F | 2.08E-03 | -6.121 | 6.121 |
| EPHX3 | 2.08E-03 | -2.856 | 2.856 |
| MTUS1 | 2.08E-03 | -1.554 | 1.554 |
| RAB25 | 2.08E-03 | -3.313 | 3.313 |
| BRI3BP | 2.09E-03 | -1.216 | 1.216 |
| KRT33A | 2.10E-03 | -4.056 | 4.056 |
| CYP2C19 | 2.10E-03 | -4.610 | 4.610 |
| ALDH2 | 2.11E-03 | -1.486 | 1.486 |
| CHMP3 | 2.12E-03 | -1.067 | 1.067 |
| NDUFV3 | 2.13E-03 | -1.011 | 1.011 |
| CNFN | 2.15E-03 | -7.875 | 7.875 |
| RPS6KB2 | 2.15E-03 | -1.652 | 1.652 |
| KCNK1 | 2.15E-03 | -1.687 | 1.687 |
| NSDHL | 2.15E-03 | -1.273 | 1.273 |
| PTGER3 | 2.15E-03 | -1.702 | 1.702 |
| GGT6 | 2.17E-03 | -3.369 | 3.369 |
| P2RY1 | 2.20E-03 | -2.684 | 2.684 |
| FOXP2 | 2.20E-03 | -3.302 | 3.302 |
| ERBB2 | 2.21E-03 | -1.214 | 1.214 |
| KRT6B | 2.21E-03 | -3.572 | 3.572 |
| KRT13 | 2.23E-03 | -3.800 | 3.800 |
| AMD1 | 2.24E-03 | -1.334 | 1.334 |
| TRIP10 | 2.24E-03 | -1.249 | 1.249 |
| SGPP2 | 2.24E-03 | -3.786 | 3.786 |
| C10orf58 | 2.24E-03 | -2.167 | 2.167 |
| HN1 | 2.27E-03 | -1.780 | 1.780 |
| S100A14 | 2.27E-03 | -3.201 | 3.201 |
| C9orf103 | 2.28E-03 | -1.028 | 1.028 |
| BSPRY | 2.29E-03 | -5.001 | 5.001 |
| SKA1 | 2.30E-03 | -1.975 | 1.975 |
| OR7G1 | 2.35E-03 | -2.266 | 2.266 |
| JMJD7 | 2.35E-03 | -1.762 | 1.762 |
| FH | 2.36E-03 | -1.409 | 1.409 |
| ARL9 | 2.36E-03 | -2.690 | 2.690 |
| KIAA1522 | 2.36E-03 | -1.218 | 1.218 |
| TP53AIP1 | 2.40E-03 | -2.800 | 2.800 |
| SPRR2E | 2.40E-03 | -6.198 | 6.198 |
| EPHB6 | 2.41E-03 | -1.569 | 1.569 |
| ABLIM2 | 2.44E-03 | -3.288 | 3.288 |
| MYCBP2 | 2.45E-03 | -1.724 | 1.724 |
| ZFAND2B | 2.47E-03 | -1.349 | 1.349 |
| OXR1 | 2.47E-03 | -2.119 | 2.119 |
| DNAH9 | 2.47E-03 | -3.398 | 3.398 |
| TUBG1 | 2.49E-03 | -1.241 | 1.241 |
| PSAT1 | 2.49E-03 | -2.179 | 2.179 |
| AFG3L2 | 2.49E-03 | -1.061 | 1.061 |
| RMI1 | 2.50E-03 | -1.056 | 1.056 |
| ELOVL6 | 2.51E-03 | -1.806 | 1.806 |
| FAM83G | 2.53E-03 | -2.692 | 2.692 |
| COQ3 | 2.55E-03 | -1.414 | 1.414 |
| OCLN | 2.55E-03 | -2.076 | 2.076 |
| POF1B | 2.57E-03 | -5.370 | 5.370 |
| ACAP3 | 2.58E-03 | -1.098 | 1.098 |
| TMEM184A | 2.59E-03 | -2.330 | 2.330 |
| SOX2 | 2.60E-03 | -3.738 | 3.738 |
| BNIPL | 2.64E-03 | -2.837 | 2.837 |
| TECR | 2.65E-03 | -2.249 | 2.249 |
| TMPRSS11BNL | 2.67E-03 | -3.929 | 3.929 |
| LMNA | 2.69E-03 | -1.014 | 1.014 |
| FAM120C | 2.70E-03 | -2.434 | 2.434 |
| HMGCR | 2.70E-03 | -2.377 | 2.377 |
| TMEM52 | 2.70E-03 | -3.086 | 3.086 |
| NEFM | 2.70E-03 | -2.973 | 2.973 |
| GFOD2 | 2.71E-03 | -1.371 | 1.371 |
| ECT2 | 2.72E-03 | -1.359 | 1.359 |
| PPP2R5A | 2.73E-03 | -1.582 | 1.582 |
| TALDO1 | 2.73E-03 | -1.329 | 1.329 |
| NIPAL4 | 2.73E-03 | -2.969 | 2.969 |
| SLC16A14 | 2.74E-03 | -1.773 | 1.773 |
| ARHGAP27 | 2.74E-03 | -1.796 | 1.796 |
| STK39 | 2.75E-03 | -3.106 | 3.106 |
| PLD1 | 2.76E-03 | -2.304 | 2.304 |
| RAB11FIP1 | 2.76E-03 | -2.517 | 2.517 |
| LYPD3 | 2.77E-03 | -3.886 | 3.886 |
| SAR1B | 2.77E-03 | -1.371 | 1.371 |
| HSD17B8 | 2.77E-03 | -1.460 | 1.460 |
| RSPH9 | 2.78E-03 | -1.057 | 1.057 |
| DENND2C | 2.78E-03 | -2.597 | 2.597 |
| AURKB | 2.78E-03 | -2.193 | 2.193 |
| CARD6 | 2.79E-03 | -1.433 | 1.433 |
| ZDHHC13 | 2.79E-03 | -1.907 | 1.907 |
| CDCA5 | 2.79E-03 | -1.477 | 1.477 |
| SLC25A23 | 2.80E-03 | -1.743 | 1.743 |
| DENND2D | 2.80E-03 | -1.858 | 1.858 |
| HIPK2 | 2.80E-03 | -2.064 | 2.064 |
| CHAF1B | 2.81E-03 | -1.182 | 1.182 |
| SLC39A2 | 2.82E-03 | -4.576 | 4.576 |
| MALL | 2.82E-03 | -3.160 | 3.160 |
| HOPX | 2.83E-03 | -5.210 | 5.210 |
| FLJ41649 | 2.85E-03 | -2.021 | 2.021 |
| ICT1 | 2.86E-03 | -1.060 | 1.060 |
| AIMP2 | 2.87E-03 | -1.029 | 1.029 |
| TM4SF1 | 2.87E-03 | -1.696 | 1.696 |
| ATP10B | 2.88E-03 | -4.431 | 4.431 |
| HRASLS2 | 2.89E-03 | -2.658 | 2.658 |
| CTNNBIP1 | 2.90E-03 | -3.285 | 3.285 |
| SORD | 2.90E-03 | -3.851 | 3.851 |
| KIAA1468 | 2.90E-03 | -1.298 | 1.298 |
| GLTP | 2.90E-03 | -3.519 | 3.519 |
| CENPP | 2.91E-03 | -2.564 | 2.564 |
| MYB | 2.94E-03 | -1.907 | 1.907 |
| L3MBTL4 | 2.95E-03 | -2.190 | 2.190 |
| WNK1 | 2.97E-03 | -1.544 | 1.544 |
| OAT | 2.97E-03 | -1.062 | 1.062 |
| KLF3 | 3.00E-03 | -1.872 | 1.872 |
| GRB7 | 3.01E-03 | -1.542 | 1.542 |
| RHCG | 3.02E-03 | -4.661 | 4.661 |
| LDLR | 3.02E-03 | -2.340 | 2.340 |
| KCP | 3.03E-03 | -1.223 | 1.223 |
| DBNDD1 | 3.04E-03 | -2.712 | 2.712 |
| POLR3B | 3.06E-03 | -1.389 | 1.389 |
| MDH2 | 3.06E-03 | -1.135 | 1.135 |
| PRODH | 3.08E-03 | -2.215 | 2.215 |
| KRT31 | 3.08E-03 | -6.727 | 6.727 |
| SIAH2 | 3.08E-03 | -1.758 | 1.758 |
| C12orf66 | 3.08E-03 | -1.216 | 1.216 |
| MIF4GD | 3.10E-03 | -1.284 | 1.284 |
| ACO2 | 3.11E-03 | -1.107 | 1.107 |
| TREX1 | 3.11E-03 | -1.528 | 1.528 |
| PPP2R2A | 3.12E-03 | -1.620 | 1.620 |
| C9orf24 | 3.12E-03 | -1.751 | 1.751 |
| IL34 | 3.12E-03 | -2.131 | 2.131 |
| CYC1 | 3.13E-03 | -1.543 | 1.543 |
| WIBG | 3.14E-03 | -1.073 | 1.073 |
| ACR | 3.16E-03 | -1.279 | 1.279 |
| OGDH | 3.16E-03 | -1.053 | 1.053 |
| CDC25A | 3.16E-03 | -1.536 | 1.536 |
| GPR45 | 3.16E-03 | -4.245 | 4.245 |
| FAM53B | 3.17E-03 | -1.121 | 1.121 |
| PERP | 3.17E-03 | -1.635 | 1.635 |
| NCOA1 | 3.18E-03 | -1.010 | 1.010 |
| EXPH5 | 3.18E-03 | -3.055 | 3.055 |
| CLYBL | 3.18E-03 | -1.326 | 1.326 |
| DSG1 | 3.22E-03 | -9.818 | 9.818 |
| SLBP | 3.23E-03 | -1.098 | 1.098 |
| TMEM154 | 3.25E-03 | -2.356 | 2.356 |
| PTK6 | 3.27E-03 | -4.191 | 4.191 |
| STRBP | 3.28E-03 | -1.362 | 1.362 |
| TUBA4A | 3.28E-03 | -2.090 | 2.090 |
| CHAF1A | 3.29E-03 | -1.180 | 1.180 |
| NDUFV2 | 3.30E-03 | -1.105 | 1.105 |
| POU3F1 | 3.30E-03 | -2.483 | 2.483 |
| PXMP2 | 3.31E-03 | -5.886 | 5.886 |
| ALDOC | 3.32E-03 | -1.960 | 1.960 |
| PRRT4 | 3.32E-03 | -1.988 | 1.988 |
| SERAC1 | 3.34E-03 | -1.114 | 1.114 |
| CCDC124 | 3.35E-03 | -1.019 | 1.019 |
| PPP1CB | 3.37E-03 | -1.383 | 1.383 |
| ASF1B | 3.38E-03 | -1.462 | 1.462 |
| PRDX6 | 3.39E-03 | -1.592 | 1.592 |
| CIDEB | 3.39E-03 | -1.293 | 1.293 |
| LGALSL | 3.41E-03 | -3.965 | 3.965 |
| LRRC1 | 3.41E-03 | -1.351 | 1.351 |
| E2F1 | 3.42E-03 | -1.632 | 1.632 |
| CSRP2 | 3.42E-03 | -3.134 | 3.134 |
| NUSAP1 | 3.43E-03 | -1.624 | 1.624 |
| TAS1R3 | 3.44E-03 | -3.079 | 3.079 |
| ANKRD9 | 3.44E-03 | -1.394 | 1.394 |
| EIF5A | 3.45E-03 | -1.181 | 1.181 |
| RNF167 | 3.46E-03 | -1.407 | 1.407 |
| KIAA1671 | 3.46E-03 | -1.614 | 1.614 |
| GBP6 | 3.47E-03 | -3.741 | 3.741 |
| ABHD11 | 3.47E-03 | -1.094 | 1.094 |
| FOXN1 | 3.49E-03 | -1.784 | 1.784 |
| GPT2 | 3.50E-03 | -2.710 | 2.710 |
| TUBB8 | 3.50E-03 | -2.122 | 2.122 |
| DSG3 | 3.51E-03 | -3.098 | 3.098 |
| FAM126B | 3.53E-03 | -1.059 | 1.059 |
| HDHD3 | 3.53E-03 | -1.464 | 1.464 |
| RREB1 | 3.54E-03 | -1.142 | 1.142 |
| MFHAS1 | 3.58E-03 | -1.677 | 1.677 |
| C7orf46 | 3.61E-03 | -1.548 | 1.548 |
| VMAC | 3.62E-03 | -1.089 | 1.089 |
| NMU | 3.63E-03 | -2.366 | 2.366 |
| CPEB2 | 3.64E-03 | -2.055 | 2.055 |
| AHNAK2 | 3.64E-03 | -2.265 | 2.265 |
| LAMTOR3 | 3.64E-03 | -1.048 | 1.048 |
| MFSD6 | 3.64E-03 | -1.491 | 1.491 |
| LYSMD4 | 3.64E-03 | -1.054 | 1.054 |
| AGSK1 | 3.66E-03 | -2.344 | 2.344 |
| MBNL3 | 3.66E-03 | -1.479 | 1.479 |
| EPN2 | 3.67E-03 | -1.251 | 1.251 |
| ITPKC | 3.68E-03 | -1.831 | 1.831 |
| RDH13 | 3.69E-03 | -1.453 | 1.453 |
| ANKRD5 | 3.70E-03 | -1.144 | 1.144 |
| WDR33 | 3.70E-03 | -1.144 | 1.144 |
| MLIP | 3.70E-03 | -5.454 | 5.454 |
| RNF141 | 3.71E-03 | -1.369 | 1.369 |
| E2F8 | 3.71E-03 | -4.336 | 4.336 |
| PIGN | 3.72E-03 | -1.492 | 1.492 |
| MST1R | 3.72E-03 | -2.990 | 2.990 |
| RANGAP1 | 3.72E-03 | -1.343 | 1.343 |
| MIDN | 3.72E-03 | -1.398 | 1.398 |
| DAAM1 | 3.73E-03 | -1.919 | 1.919 |
| AFTPH | 3.73E-03 | -1.041 | 1.041 |
| PPP2R2C | 3.73E-03 | -5.396 | 5.396 |
| POU3F3 | 3.74E-03 | -2.237 | 2.237 |
| A2ML1 | 3.74E-03 | -6.315 | 6.315 |
| MVD | 3.75E-03 | -1.213 | 1.213 |
| SOX6 | 3.76E-03 | -2.818 | 2.818 |
| AIM1L | 3.78E-03 | -3.358 | 3.358 |
| ANKRD35 | 3.79E-03 | -1.970 | 1.970 |
| SNX21 | 3.79E-03 | -1.199 | 1.199 |
| TRIM29 | 3.80E-03 | -1.982 | 1.982 |
| COQ9 | 3.80E-03 | -1.006 | 1.006 |
| HIST1H1A | 3.80E-03 | -2.571 | 2.571 |
| CA13 | 3.81E-03 | -1.049 | 1.049 |
| ATP5G3 | 3.81E-03 | -1.107 | 1.107 |
| IL22RA1 | 3.82E-03 | -4.593 | 4.593 |
| YWHAZ | 3.82E-03 | -2.116 | 2.116 |
| REEP6 | 3.83E-03 | -1.380 | 1.380 |
| SERBP1 | 3.84E-03 | -1.193 | 1.193 |
| NNAT | 3.85E-03 | -2.003 | 2.003 |
| FAM102A | 3.86E-03 | -1.504 | 1.504 |
| PHKA1 | 3.87E-03 | -1.425 | 1.425 |
| GLTPD1 | 3.87E-03 | -1.352 | 1.352 |
| C19orf73 | 3.88E-03 | -1.168 | 1.168 |
| DYNLL1 | 3.88E-03 | -1.690 | 1.690 |
| NBEAL2 | 3.88E-03 | -1.942 | 1.942 |
| ATP5G1 | 3.88E-03 | -1.570 | 1.570 |
| VPS37B | 3.89E-03 | -1.035 | 1.035 |
| SCNN1B | 3.93E-03 | -4.981 | 4.981 |
| SASH1 | 3.93E-03 | -1.395 | 1.395 |
| ANXA8L2 | 3.93E-03 | -2.084 | 2.084 |
| OSBPL1A | 3.95E-03 | -1.363 | 1.363 |
| DEGS1 | 3.95E-03 | -1.343 | 1.343 |
| CSE1L | 3.95E-03 | -1.114 | 1.114 |
| CCDC120 | 3.98E-03 | -1.397 | 1.397 |
| PAIP2B | 3.98E-03 | -2.269 | 2.269 |
| AP1B1 | 3.99E-03 | -1.303 | 1.303 |
| DHRS11 | 3.99E-03 | -1.512 | 1.512 |
| MAFA | 4.00E-03 | -2.708 | 2.708 |
| ARL4D | 4.01E-03 | -1.803 | 1.803 |
| SUSD4 | 4.02E-03 | -2.565 | 2.565 |
| TRIM47 | 4.02E-03 | -1.111 | 1.111 |
| CCDC6 | 4.03E-03 | -1.057 | 1.057 |
| VIT | 4.03E-03 | -2.250 | 2.250 |
| SLK | 4.03E-03 | -1.687 | 1.687 |
| TEX2 | 4.06E-03 | -1.041 | 1.041 |
| STARD5 | 4.07E-03 | -1.914 | 1.914 |
| KRT80 | 4.08E-03 | -4.284 | 4.284 |
| VTA1 | 4.09E-03 | -1.300 | 1.300 |
| EPB41L4B | 4.09E-03 | -3.154 | 3.154 |
| TMEM30B | 4.10E-03 | -1.639 | 1.639 |
| NFKBIB | 4.10E-03 | -1.115 | 1.115 |
| GUK1 | 4.11E-03 | -1.213 | 1.213 |
| TMPRSS11B | 4.16E-03 | -6.551 | 6.551 |
| SLC16A9 | 4.17E-03 | -3.920 | 3.920 |
| CCDC9 | 4.17E-03 | -2.085 | 2.085 |
| ACTR3 | 4.21E-03 | -1.442 | 1.442 |
| ROR1 | 4.22E-03 | -1.798 | 1.798 |
| EBPL | 4.22E-03 | -1.304 | 1.304 |
| C1orf21 | 4.23E-03 | -1.281 | 1.281 |
| IKZF5 | 4.23E-03 | -1.140 | 1.140 |
| LRRFIP2 | 4.23E-03 | -1.347 | 1.347 |
| GPR115 | 4.23E-03 | -2.255 | 2.255 |
| RNF187 | 4.24E-03 | -1.001 | 1.001 |
| RMND5B | 4.25E-03 | -1.548 | 1.548 |
| NT5C3 | 4.28E-03 | -1.233 | 1.233 |
| KRT5 | 4.28E-03 | -1.656 | 1.656 |
| KRT6C | 4.29E-03 | -3.388 | 3.388 |
| PLEKHM1 | 4.29E-03 | -1.292 | 1.292 |
| DNAJB1 | 4.31E-03 | -1.543 | 1.543 |
| ALDH3B2 | 4.31E-03 | -3.480 | 3.480 |
| ZNF185 | 4.32E-03 | -2.665 | 2.665 |
| ZXDA | 4.32E-03 | -2.233 | 2.233 |
| EDN1 | 4.32E-03 | -2.295 | 2.295 |
| HPSE | 4.34E-03 | -4.051 | 4.051 |
| SPRR2D | 4.34E-03 | -6.533 | 6.533 |
| TMEM14A | 4.35E-03 | -1.536 | 1.536 |
| SH3GL3 | 4.35E-03 | -3.921 | 3.921 |
| Q8VGA8 | 4.36E-03 | -2.092 | 2.092 |
| LNX1 | 4.39E-03 | -2.172 | 2.172 |
| RHOV | 4.39E-03 | -1.924 | 1.924 |
| MANEAL | 4.40E-03 | -1.884 | 1.884 |
| C21orf15 | 4.41E-03 | -4.645 | 4.645 |
| GAS2L1 | 4.41E-03 | -1.196 | 1.196 |
| DCAF11 | 4.43E-03 | -1.148 | 1.148 |
| CYP2F1 | 4.44E-03 | -8.806 | 8.806 |
| PLEKHA5 | 4.44E-03 | -2.885 | 2.885 |
| PALMD | 4.44E-03 | -1.637 | 1.637 |
| CYCS | 4.45E-03 | -1.669 | 1.669 |
| PLEKHF2 | 4.45E-03 | -1.443 | 1.443 |
| COX5A | 4.45E-03 | -1.125 | 1.125 |
| CDHR1 | 4.45E-03 | -6.012 | 6.012 |
| MAPKAPK3 | 4.46E-03 | -1.543 | 1.543 |
| PSMD6 | 4.46E-03 | -1.135 | 1.135 |
| KIAA1737 | 4.47E-03 | -1.322 | 1.322 |
| SLC22A23 | 4.47E-03 | -1.636 | 1.636 |
| IGF2BP3 | 4.48E-03 | -1.679 | 1.679 |
| DAPL1 | 4.51E-03 | -3.720 | 3.720 |
| SULT2B1 | 4.52E-03 | -5.416 | 5.416 |
| FAM22A | 4.52E-03 | -6.339 | 6.339 |
| IPPK | 4.52E-03 | -1.950 | 1.950 |
| MRPL12 | 4.53E-03 | -1.028 | 1.028 |
| CCDC90A | 4.53E-03 | -1.245 | 1.245 |
| SH3GLB2 | 4.55E-03 | -1.415 | 1.415 |
| PVT1 | 4.56E-03 | -1.676 | 1.676 |
| TPD52 | 4.57E-03 | -1.436 | 1.436 |
| BCHE | 4.57E-03 | -2.995 | 2.995 |
| KRT16 | 4.58E-03 | -3.496 | 3.496 |
| KIAA0232 | 4.58E-03 | -1.049 | 1.049 |
| FAM189A2 | 4.59E-03 | -1.266 | 1.266 |
| PLK3 | 4.60E-03 | -1.388 | 1.388 |
| ARHGEF4 | 4.62E-03 | -1.555 | 1.555 |
| TOLLIP | 4.64E-03 | -1.544 | 1.544 |
| MINK1 | 4.64E-03 | -1.496 | 1.496 |
| PPA1 | 4.65E-03 | -1.036 | 1.036 |
| SEPT5 | 4.65E-03 | -1.683 | 1.683 |
| ADIPOR2 | 4.66E-03 | -1.286 | 1.286 |
| PKP3 | 4.67E-03 | -2.847 | 2.847 |
| ANKRD57 | 4.67E-03 | -2.611 | 2.611 |
| CMPK1 | 4.68E-03 | -1.039 | 1.039 |
| CYP2C18 | 4.69E-03 | -4.603 | 4.603 |
| ZNF655 | 4.70E-03 | -1.217 | 1.217 |
| KRT26 | 4.71E-03 | -6.382 | 6.382 |
| ATP1B1 | 4.71E-03 | -2.420 | 2.420 |
| PPARGC1B | 4.73E-03 | -1.630 | 1.630 |
| NDUFB7 | 4.73E-03 | -1.023 | 1.023 |
| HOMER2 | 4.74E-03 | -1.299 | 1.299 |
| TUBA3C | 4.76E-03 | -1.617 | 1.617 |
| GANC | 4.78E-03 | -1.068 | 1.068 |
| EXO1 | 4.79E-03 | -1.586 | 1.586 |
| AHSA1 | 4.80E-03 | -1.013 | 1.013 |
| LIPE | 4.80E-03 | -1.115 | 1.115 |
| MCM4 | 4.81E-03 | -1.379 | 1.379 |
| GSTZ1 | 4.81E-03 | -1.107 | 1.107 |
| MAPK3 | 4.82E-03 | -1.618 | 1.618 |
| BRCA1 | 4.82E-03 | -1.171 | 1.171 |
| CNPPD1 | 4.85E-03 | -1.551 | 1.551 |
| FAM57A | 4.86E-03 | -1.693 | 1.693 |
| FOXE1 | 4.86E-03 | -3.886 | 3.886 |
| EVPLL | 4.88E-03 | -3.846 | 3.846 |
| POLR3G | 4.89E-03 | -1.660 | 1.660 |
| MSMO1 | 4.92E-03 | -1.673 | 1.673 |
| KRT10 | 4.95E-03 | -3.087 | 3.087 |
| DUOXA1 | 4.97E-03 | -2.052 | 2.052 |
| ITPR2 | 4.97E-03 | -1.422 | 1.422 |
| GDPD2 | 4.98E-03 | -4.547 | 4.547 |
| STK24 | 4.99E-03 | -1.361 | 1.361 |
| VDAC1 | 5.02E-03 | -1.286 | 1.286 |
| LRRC20 | 5.02E-03 | -2.796 | 2.796 |
| PPL | 5.02E-03 | -4.494 | 4.494 |
| RAC1 | 5.04E-03 | -1.268 | 1.268 |
| ZNF165 | 5.05E-03 | -1.298 | 1.298 |
| TMEM79 | 5.07E-03 | -3.965 | 3.965 |
| GPRC5D | 5.08E-03 | -4.657 | 4.657 |
| ABCD3 | 5.09E-03 | -1.226 | 1.226 |
| TPI1 | 5.10E-03 | -1.177 | 1.177 |
| ANXA7 | 5.11E-03 | -1.063 | 1.063 |
| GAN | 5.13E-03 | -1.963 | 1.963 |
| SDCBP2 | 5.14E-03 | -3.040 | 3.040 |
| INCENP | 5.15E-03 | -1.462 | 1.462 |
| SMPD2 | 5.15E-03 | -2.177 | 2.177 |
| C2orf29 | 5.17E-03 | -1.031 | 1.031 |
| B3GNT8 | 5.19E-03 | -2.206 | 2.206 |
| SAMD10 | 5.19E-03 | -1.504 | 1.504 |
| FBXL16 | 5.19E-03 | -2.672 | 2.672 |
| SPACA4 | 5.20E-03 | -4.109 | 4.109 |
| TMOD3 | 5.21E-03 | -1.446 | 1.446 |
| LMTK2 | 5.24E-03 | -1.346 | 1.346 |
| CDC46 | 5.24E-03 | -1.379 | 1.379 |
| C20orf24 | 5.24E-03 | -1.670 | 1.670 |
| PGM2 | 5.25E-03 | -1.880 | 1.880 |
| C10orf57 | 5.26E-03 | -1.331 | 1.331 |
| ANKRD22 | 5.26E-03 | -4.632 | 4.632 |
| C1orf55 | 5.27E-03 | -1.088 | 1.088 |
| C20orf111 | 5.29E-03 | -1.255 | 1.255 |
| C4orf3 | 5.30E-03 | -1.923 | 1.923 |
| HMGN5 | 5.30E-03 | -1.300 | 1.300 |
| ATP5A1 | 5.30E-03 | -1.041 | 1.041 |
| NAAA | 5.31E-03 | -1.274 | 1.274 |
| HSD11B2 | 5.35E-03 | -3.114 | 3.114 |
| FGFR3 | 5.37E-03 | -2.292 | 2.292 |
| SLMO2 | 5.37E-03 | -1.214 | 1.214 |
| RNF208 | 5.39E-03 | -1.635 | 1.635 |
| ASCL2 | 5.41E-03 | -2.911 | 2.911 |
| MAFB | 5.43E-03 | -1.667 | 1.667 |
| TTK | 5.44E-03 | -2.129 | 2.129 |
| PHLDB3 | 5.45E-03 | -1.483 | 1.483 |
| SYNGR2 | 5.47E-03 | -1.086 | 1.086 |
| ESPN | 5.48E-03 | -1.788 | 1.788 |
| BCKDHB | 5.49E-03 | -1.136 | 1.136 |
| KRT14 | 5.50E-03 | -2.092 | 2.092 |
| IRAK1 | 5.51E-03 | -1.042 | 1.042 |
| SUCLA2 | 5.52E-03 | -1.523 | 1.523 |
| TICAM1 | 5.56E-03 | -1.521 | 1.521 |
| ZNRF2 | 5.58E-03 | -1.098 | 1.098 |
| RSPH3 | 5.59E-03 | -1.120 | 1.120 |
| CENPN | 5.62E-03 | -1.531 | 1.531 |
| KLC3 | 5.63E-03 | -2.283 | 2.283 |
| AKAP1 | 5.64E-03 | -1.253 | 1.253 |
| OVOL1 | 5.65E-03 | -2.476 | 2.476 |
| PCMT1 | 5.66E-03 | -1.105 | 1.105 |
| ATL3 | 5.66E-03 | -1.135 | 1.135 |
| PLP1 | 5.68E-03 | -2.905 | 2.905 |
| DYNLT3 | 5.68E-03 | -1.640 | 1.640 |
| DEPTOR | 5.68E-03 | -1.325 | 1.325 |
| SIRT7 | 5.69E-03 | -1.594 | 1.594 |
| CRABP2 | 5.70E-03 | -3.579 | 3.579 |
| SH2D3A | 5.71E-03 | -1.126 | 1.126 |
| STAC2 | 5.71E-03 | -4.638 | 4.638 |
| CDKN2AIP | 5.75E-03 | -1.288 | 1.288 |
| KCTD9 | 5.75E-03 | -1.288 | 1.288 |
| B3GNT5 | 5.78E-03 | -2.187 | 2.187 |
| PTGR1 | 5.81E-03 | -2.061 | 2.061 |
| BRP44 | 5.81E-03 | -1.103 | 1.103 |
| VDAC2 | 5.82E-03 | -1.361 | 1.361 |
| USP53 | 5.82E-03 | -1.292 | 1.292 |
| ACOX3 | 5.82E-03 | -1.776 | 1.776 |
| FOXM1 | 5.85E-03 | -1.564 | 1.564 |
| CSDA | 5.87E-03 | -1.103 | 1.103 |
| ACAT1 | 5.87E-03 | -1.553 | 1.553 |
| OIP5 | 5.87E-03 | -1.674 | 1.674 |
| AADAC | 5.88E-03 | -10.198 | 10.198 |
| BNIP3 | 5.88E-03 | -1.809 | 1.809 |
| GRTP1 | 5.90E-03 | -1.564 | 1.564 |
| BASP1P1 | 5.90E-03 | -4.036 | 4.036 |
| GAB1 | 5.92E-03 | -1.315 | 1.315 |
| FDPSL2A | 5.92E-03 | -1.214 | 1.214 |
| RFC3 | 5.94E-03 | -1.025 | 1.025 |
| EFNA3 | 5.95E-03 | -2.986 | 2.986 |
| TACSTD2 | 5.96E-03 | -1.639 | 1.639 |
| AMFR | 5.96E-03 | -1.141 | 1.141 |
| ANXA1 | 5.98E-03 | -1.212 | 1.212 |
| RTN4IP1 | 5.99E-03 | -1.032 | 1.032 |
| CEP76 | 6.01E-03 | -1.424 | 1.424 |
| UBE2G1 | 6.01E-03 | -1.613 | 1.613 |
| ADM | 6.02E-03 | -3.336 | 3.336 |
| NEK2 | 6.07E-03 | -2.046 | 2.046 |
| EBP | 6.07E-03 | -1.294 | 1.294 |
| TUBB2A | 6.07E-03 | -2.540 | 2.540 |
| NDRG1 | 6.08E-03 | -1.796 | 1.796 |
| HTATIP2 | 6.12E-03 | -1.772 | 1.772 |
| CTRB2 | 6.13E-03 | -3.930 | 3.930 |
| CAPG | 6.14E-03 | -1.441 | 1.441 |
| EIF3J | 6.14E-03 | -1.237 | 1.237 |
| PSEN2 | 6.15E-03 | -1.031 | 1.031 |
| C15orf42 | 6.15E-03 | -2.068 | 2.068 |
| HSD17B7 | 6.15E-03 | -1.161 | 1.161 |
| ACE2 | 6.16E-03 | -3.865 | 3.865 |
| CHP | 6.17E-03 | -1.765 | 1.765 |
| CLTB | 6.17E-03 | -1.509 | 1.509 |
| SSX2IP | 6.18E-03 | -1.762 | 1.762 |
| PRSS2 | 6.19E-03 | -5.729 | 5.729 |
| LYPD5 | 6.20E-03 | -4.451 | 4.451 |
| PPIA | 6.21E-03 | -1.041 | 1.041 |
| ELOVL1 | 6.22E-03 | -2.004 | 2.004 |
| NDOR1 | 6.24E-03 | -1.952 | 1.952 |
| SERPINB2 | 6.27E-03 | -6.562 | 6.562 |
| SLC6A9 | 6.27E-03 | -2.332 | 2.332 |
| SYPL1 | 6.30E-03 | -1.195 | 1.195 |
| TGM5 | 6.30E-03 | -4.414 | 4.414 |
| FAM3B | 6.32E-03 | -7.863 | 7.863 |
| GRIN2A | 6.33E-03 | -3.570 | 3.570 |
| AIM1 | 6.34E-03 | -1.601 | 1.601 |
| EZR | 6.34E-03 | -2.116 | 2.116 |
| ADAT2 | 6.35E-03 | -2.165 | 2.165 |
| CDH26 | 6.36E-03 | -3.053 | 3.053 |
| DOCK9 | 6.37E-03 | -1.329 | 1.329 |
| IKZF2 | 6.39E-03 | -1.479 | 1.479 |
| STXBP2 | 6.39E-03 | -1.136 | 1.136 |
| SRP68 | 6.40E-03 | -1.172 | 1.172 |
| TUBAL3 | 6.44E-03 | -1.903 | 1.903 |
| AKR1C1 | 6.45E-03 | -3.873 | 3.873 |
| SCAMP2 | 6.48E-03 | -1.193 | 1.193 |
| C15orf41 | 6.48E-03 | -1.299 | 1.299 |
| PHLPP1 | 6.51E-03 | -1.117 | 1.117 |
| KIF11 | 6.52E-03 | -1.558 | 1.558 |
| UQCRFS1 | 6.55E-03 | -1.032 | 1.032 |
| CPEB3 | 6.57E-03 | -1.242 | 1.242 |
| HCCS | 6.60E-03 | -1.236 | 1.236 |
| DTX2 | 6.60E-03 | -1.444 | 1.444 |
| RAB6C | 6.61E-03 | -1.039 | 1.039 |
| GJB5 | 6.63E-03 | -2.176 | 2.176 |
| CCDC167 | 6.65E-03 | -1.120 | 1.120 |
| ZCCHC6 | 6.66E-03 | -1.321 | 1.321 |
| ATP6V1D | 6.67E-03 | -1.266 | 1.266 |
| TPI1P2 | 6.67E-03 | -1.213 | 1.213 |
| KREMEN1 | 6.71E-03 | -1.929 | 1.929 |
| AK4 | 6.72E-03 | -1.262 | 1.262 |
| UCHL5 | 6.72E-03 | -1.111 | 1.111 |
| MT1X | 6.72E-03 | -2.081 | 2.081 |
| PRC1 | 6.72E-03 | -1.304 | 1.304 |
| PXMP4 | 6.74E-03 | -1.092 | 1.092 |
| RAB11FIP4 | 6.75E-03 | -1.051 | 1.051 |
| KIAA0513 | 6.76E-03 | -1.012 | 1.012 |
| SEMA4D | 6.76E-03 | -1.699 | 1.699 |
| ZBTB42 | 6.77E-03 | -1.032 | 1.032 |
| TMEM63C | 6.79E-03 | -3.233 | 3.233 |
| SYK | 6.81E-03 | -1.345 | 1.345 |
| KRT16P2 | 6.81E-03 | -2.541 | 2.541 |
| NIPAL3 | 6.83E-03 | -1.395 | 1.395 |
| MAPK9 | 6.84E-03 | -1.067 | 1.067 |
| RIOK3 | 6.85E-03 | -1.069 | 1.069 |
| RRM2 | 6.85E-03 | -1.573 | 1.573 |
| ASPG | 6.87E-03 | -4.237 | 4.237 |
| PRDX2 | 6.89E-03 | -1.181 | 1.181 |
| GINS2 | 6.89E-03 | -1.321 | 1.321 |
| RIMS3 | 6.91E-03 | -1.516 | 1.516 |
| FUT6 | 6.93E-03 | -4.182 | 4.182 |
| MARK2 | 6.93E-03 | -1.029 | 1.029 |
| NAPA | 6.95E-03 | -1.204 | 1.204 |
| CAMKK1 | 6.97E-03 | -1.734 | 1.734 |
| HSPA2 | 6.98E-03 | -1.472 | 1.472 |
| RMND5A | 6.99E-03 | -1.639 | 1.639 |
| C1orf226 | 6.99E-03 | -1.571 | 1.571 |
| UGT1A6 | 7.00E-03 | -5.001 | 5.001 |
| GOLGA4 | 7.02E-03 | -1.156 | 1.156 |
| SHROOM3 | 7.05E-03 | -3.012 | 3.012 |
| PSORS1C2 | 7.05E-03 | -5.555 | 5.555 |
| SCRIB | 7.06E-03 | -1.265 | 1.265 |
| PPP1R3C | 7.06E-03 | -1.960 | 1.960 |
| GSPT1 | 7.10E-03 | -1.030 | 1.030 |
| NAPRT1 | 7.10E-03 | -2.084 | 2.084 |
| CCNB2 | 7.11E-03 | -1.568 | 1.568 |
| C2orf55 | 7.11E-03 | -1.691 | 1.691 |
| SH2D4A | 7.13E-03 | -1.978 | 1.978 |
| DOHH | 7.15E-03 | -1.012 | 1.012 |
| UGT1A8 | 7.17E-03 | -5.168 | 5.168 |
| KDM2B | 7.17E-03 | -1.137 | 1.137 |
| USH1G | 7.18E-03 | -2.398 | 2.398 |
| CAT | 7.22E-03 | -1.028 | 1.028 |
| C12orf29 | 7.23E-03 | -1.377 | 1.377 |
| C8orf73 | 7.23E-03 | -1.270 | 1.270 |
| SLC44A2 | 7.24E-03 | -1.046 | 1.046 |
| NCCRP1 | 7.27E-03 | -4.244 | 4.244 |
| MYZAP | 7.27E-03 | -4.132 | 4.132 |
| ESCO2 | 7.27E-03 | -2.085 | 2.085 |
| ETNK2 | 7.28E-03 | -2.701 | 2.701 |
| MAMDC2 | 7.29E-03 | -2.704 | 2.704 |
| RPRML | 7.30E-03 | -4.800 | 4.800 |
| COX5B | 7.32E-03 | -1.052 | 1.052 |
| GSTP1 | 7.32E-03 | -1.113 | 1.113 |
| GPD1L | 7.34E-03 | -1.491 | 1.491 |
| GNG5 | 7.34E-03 | -1.013 | 1.013 |
| RAET1E | 7.37E-03 | -6.675 | 6.675 |
| C6orf132 | 7.37E-03 | -3.622 | 3.622 |
| CENPH | 7.38E-03 | -1.225 | 1.225 |
| PLEKHA1 | 7.38E-03 | -1.014 | 1.014 |
| CYP4F12 | 7.39E-03 | -2.727 | 2.727 |
| DIP2B | 7.39E-03 | -1.455 | 1.455 |
| TOM1L1 | 7.40E-03 | -1.280 | 1.280 |
| EEF2K | 7.43E-03 | -1.130 | 1.130 |
| FEM1A | 7.44E-03 | -1.103 | 1.103 |
| TYRO3 | 7.44E-03 | -2.326 | 2.326 |
| PDZK1IP1 | 7.44E-03 | -2.242 | 2.242 |
| ADIPOR1 | 7.45E-03 | -1.214 | 1.214 |
| L2HGDH | 7.45E-03 | -1.241 | 1.241 |
| PAFAH1B1 | 7.45E-03 | -1.298 | 1.298 |
| BAG1 | 7.46E-03 | -1.129 | 1.129 |
| BCMO1 | 7.46E-03 | -4.033 | 4.033 |
| SH3BP1 | 7.47E-03 | -1.127 | 1.127 |
| AKR1CL1 | 7.48E-03 | -3.888 | 3.888 |
| FAM180B | 7.51E-03 | -3.398 | 3.398 |
| ORC6 | 7.52E-03 | -1.568 | 1.568 |
| NOP16 | 7.54E-03 | -1.251 | 1.251 |
| SIRT2 | 7.58E-03 | -1.193 | 1.193 |
| DUOX1 | 7.58E-03 | -1.969 | 1.969 |
| SNAR-G1 | 7.59E-03 | -1.568 | 1.568 |
| TGFBR3 | 7.60E-03 | -1.434 | 1.434 |
| PPP2CA | 7.62E-03 | -1.101 | 1.101 |
| FAM129B | 7.63E-03 | -1.456 | 1.456 |
| OR2W5 | 7.67E-03 | -5.983 | 5.983 |
| CDC45 | 7.69E-03 | -1.286 | 1.286 |
| BIRC5 | 7.70E-03 | -1.627 | 1.627 |
| ARG2 | 7.71E-03 | -2.111 | 2.111 |
| OSBPL2 | 7.73E-03 | -1.263 | 1.263 |
| DUSP5 | 7.73E-03 | -1.824 | 1.824 |
| MYCL1 | 7.74E-03 | -1.933 | 1.933 |
| RND3 | 7.75E-03 | -2.434 | 2.434 |
| MELK | 7.76E-03 | -1.519 | 1.519 |
| FONG | 7.76E-03 | -1.653 | 1.653 |
| RASSF3 | 7.78E-03 | -4.404 | 4.404 |
| CERS3 | 7.78E-03 | -1.822 | 1.822 |
| TRIM35 | 7.79E-03 | -1.017 | 1.017 |
| C1orf116 | 7.81E-03 | -2.033 | 2.033 |
| MAP3K8 | 7.81E-03 | -1.877 | 1.877 |
| MVK | 7.83E-03 | -1.671 | 1.671 |
| STAU2 | 7.85E-03 | -1.074 | 1.074 |
| RNLS | 7.85E-03 | -1.195 | 1.195 |
| DTL | 7.86E-03 | -1.323 | 1.323 |
| S100A16 | 7.87E-03 | -2.183 | 2.183 |
| MAPK8IP2 | 7.89E-03 | -1.214 | 1.214 |
| RSPH1 | 7.90E-03 | -1.113 | 1.113 |
| GLRX2 | 7.90E-03 | -1.006 | 1.006 |
| ULK3 | 7.92E-03 | -1.420 | 1.420 |
| RAB35 | 7.92E-03 | -1.067 | 1.067 |
| LRRC4 | 7.92E-03 | -2.029 | 2.029 |
| DGAT2 | 7.92E-03 | -3.456 | 3.456 |
| FXYD3 | 7.93E-03 | -1.454 | 1.454 |
| VPS25 | 7.96E-03 | -1.434 | 1.434 |
| RND1 | 7.97E-03 | -2.434 | 2.434 |
| MED8 | 7.98E-03 | -1.182 | 1.182 |
| SBF1 | 8.02E-03 | -1.406 | 1.406 |
| BTN2A1 | 8.03E-03 | -1.519 | 1.519 |
| PAX9 | 8.04E-03 | -3.889 | 3.889 |
| TAGLN2 | 8.04E-03 | -1.268 | 1.268 |
| HMGA1 | 8.05E-03 | -2.234 | 2.234 |
| CCNA2 | 8.05E-03 | -1.701 | 1.701 |
| ENO1 | 8.06E-03 | -1.135 | 1.135 |
| SYTL1 | 8.06E-03 | -1.085 | 1.085 |
| NDRG2 | 8.06E-03 | -1.885 | 1.885 |
| CENPA | 8.07E-03 | -2.091 | 2.091 |
| MTF1 | 8.08E-03 | -1.352 | 1.352 |
| ADK | 8.09E-03 | -1.357 | 1.357 |
| DDX3X | 8.10E-03 | -1.369 | 1.369 |
| TSKS | 8.11E-03 | -2.089 | 2.089 |
| CYP4F2 | 8.12E-03 | -2.785 | 2.785 |
| ANKRD18B | 8.13E-03 | -1.520 | 1.520 |
| ADRB2 | 8.15E-03 | -3.925 | 3.925 |
| CXADRP3 | 8.16E-03 | -1.494 | 1.494 |
| TIMM8B | 8.18E-03 | -1.096 | 1.096 |
| MPZL3 | 8.18E-03 | -3.389 | 3.389 |
| NDUFB3 | 8.19E-03 | -1.086 | 1.086 |
| NRTN | 8.20E-03 | -2.033 | 2.033 |
| AMACR | 8.21E-03 | -1.717 | 1.717 |
| SLC47A2 | 8.22E-03 | -7.551 | 7.551 |
| PVRL4 | 8.23E-03 | -1.205 | 1.205 |
| CCL26 | 8.24E-03 | -1.592 | 1.592 |
| ACSS1 | 8.25E-03 | -1.044 | 1.044 |
| SIGLEC11 | 8.26E-03 | -2.786 | 2.786 |
| PAOX | 8.28E-03 | -1.037 | 1.037 |
| TRERF1 | 8.29E-03 | -1.238 | 1.238 |
| MYO5B | 8.29E-03 | -1.526 | 1.526 |
| AVEN | 8.31E-03 | -1.156 | 1.156 |
| MBD2 | 8.31E-03 | -1.329 | 1.329 |
| FAM108B1 | 8.32E-03 | -1.481 | 1.481 |
| MARCH7 | 8.32E-03 | -1.098 | 1.098 |
| IL1RN | 8.33E-03 | -1.858 | 1.858 |
| ACSL1 | 8.33E-03 | -1.090 | 1.090 |
| GSTM3 | 8.36E-03 | -1.412 | 1.412 |
| KCNK6 | 8.37E-03 | -1.528 | 1.528 |
| NUCB2 | 8.38E-03 | -1.726 | 1.726 |
| SSFA2 | 8.39E-03 | -1.400 | 1.400 |
| CHMP4C | 8.40E-03 | -2.468 | 2.468 |
| ELF3 | 8.41E-03 | -2.695 | 2.695 |
| MND1 | 8.42E-03 | -1.175 | 1.175 |
| RABEPK | 8.44E-03 | -2.073 | 2.073 |
| ACOT8 | 8.46E-03 | -1.661 | 1.661 |
| IL18 | 8.47E-03 | -3.657 | 3.657 |
| ZNF706 | 8.50E-03 | -1.073 | 1.073 |
| HIST1H2AI | 8.57E-03 | -1.698 | 1.698 |
| C8orf33 | 8.60E-03 | -1.722 | 1.722 |
| HRAS | 8.63E-03 | -1.265 | 1.265 |
| MARC1 | 8.65E-03 | -2.283 | 2.283 |
| MTHFD2 | 8.66E-03 | -1.043 | 1.043 |
| BRF1 | 8.68E-03 | -1.004 | 1.004 |
| C1orf183 | 8.69E-03 | -1.014 | 1.014 |
| SPPL3 | 8.69E-03 | -1.404 | 1.404 |
| TOB1 | 8.70E-03 | -1.131 | 1.131 |
| HK1 | 8.70E-03 | -1.470 | 1.470 |
| WEE1 | 8.71E-03 | -2.900 | 2.900 |
| BRP44L | 8.78E-03 | -1.198 | 1.198 |
| MANSC1 | 8.79E-03 | -1.868 | 1.868 |
| SERPINB11 | 8.81E-03 | -8.991 | 8.991 |
| NAA20 | 8.83E-03 | -1.393 | 1.393 |
| ERCC6L | 8.83E-03 | -1.467 | 1.467 |
| ARG1 | 8.84E-03 | -9.465 | 9.465 |
| FZD10 | 8.85E-03 | -3.162 | 3.162 |
| CKAP2L | 8.87E-03 | -1.813 | 1.813 |
| SORBS1 | 8.88E-03 | -2.826 | 2.826 |
| RNH1 | 8.88E-03 | -1.473 | 1.473 |
| TMPRSS11D | 8.88E-03 | -2.182 | 2.182 |
| ATP1B3 | 8.92E-03 | -1.334 | 1.334 |
| SH3BGRL2 | 8.93E-03 | -1.980 | 1.980 |
| MACROD1 | 9.01E-03 | -1.456 | 1.456 |
| DOPEY2 | 9.02E-03 | -2.038 | 2.038 |
| FOXI2 | 9.03E-03 | -1.965 | 1.965 |
| ALAD | 9.03E-03 | -1.258 | 1.258 |
| RG9MTD1 | 9.05E-03 | -1.061 | 1.061 |
| CENPE | 9.07E-03 | -1.419 | 1.419 |
| DCT | 9.07E-03 | -2.691 | 2.691 |
| CASC5 | 9.09E-03 | -1.596 | 1.596 |
| BCAS2 | 9.10E-03 | -1.248 | 1.248 |
| RBM47 | 9.12E-03 | -1.440 | 1.440 |
| RDH12 | 9.17E-03 | -9.594 | 9.594 |
| KLF5 | 9.17E-03 | -2.256 | 2.256 |
| OTUD1 | 9.18E-03 | -1.131 | 1.131 |
| MID2 | 9.18E-03 | -1.138 | 1.138 |
| IL20 | 9.19E-03 | -4.949 | 4.949 |
| ANKRD29 | 9.21E-03 | -1.754 | 1.754 |
| CWH43 | 9.22E-03 | -3.720 | 3.720 |
| CENPF | 9.27E-03 | -1.435 | 1.435 |
| KIF21A | 9.27E-03 | -2.003 | 2.003 |
| PGK1 | 9.28E-03 | -1.135 | 1.135 |
| VSIG10L | 9.31E-03 | -4.257 | 4.257 |
| VDAC3 | 9.33E-03 | -1.006 | 1.006 |
| DHCR24 | 9.34E-03 | -1.219 | 1.219 |
| CDS1 | 9.36E-03 | -1.621 | 1.621 |
| NDUFB9 | 9.39E-03 | -1.044 | 1.044 |
| CHP2 | 9.41E-03 | -8.472 | 8.472 |
| S100A11 | 9.41E-03 | -1.043 | 1.043 |
| OXGR1 | 9.42E-03 | -1.691 | 1.691 |
| UBE2T | 9.45E-03 | -1.142 | 1.142 |
| TOP2A | 9.47E-03 | -1.853 | 1.853 |
| LRRC8B | 9.50E-03 | -1.134 | 1.134 |
| CNKSR3 | 9.51E-03 | -1.837 | 1.837 |
| MUSK | 9.53E-03 | -1.560 | 1.560 |
| RAB40AL | 9.58E-03 | -2.692 | 2.692 |
| ANLN | 9.60E-03 | -1.878 | 1.878 |
| LPAR5 | 9.62E-03 | -1.962 | 1.962 |
| MKI67 | 9.62E-03 | -2.112 | 2.112 |
| NAGK | 9.63E-03 | -1.784 | 1.784 |
| KLK10 | 9.66E-03 | -6.144 | 6.144 |
| SSNA1 | 9.68E-03 | -1.009 | 1.009 |
| HECA | 9.70E-03 | -1.098 | 1.098 |
| SH3YL1 | 9.73E-03 | -1.053 | 1.053 |
| MAPT | 9.76E-03 | -4.124 | 4.124 |
| RIIAD1 | 9.77E-03 | -3.589 | 3.589 |
| GALE | 9.80E-03 | -2.162 | 2.162 |
| SRXN1 | 9.81E-03 | -1.574 | 1.574 |
| CHCHD7 | 9.84E-03 | -1.310 | 1.310 |
| CTPS | 9.90E-03 | -1.009 | 1.009 |
| BAG5 | 9.90E-03 | -1.038 | 1.038 |
| REEP4 | 9.91E-03 | -1.778 | 1.778 |
| PRSS3 | 9.91E-03 | -6.012 | 6.012 |
| GMPPB | 9.91E-03 | -1.145 | 1.145 |
| CDK1 | 9.95E-03 | -1.583 | 1.583 |
| HSPB1 | 9.95E-03 | -1.307 | 1.307 |
| ZBTB20-AS1 | 9.99E-03 | -2.528 | 2.528 |

**Supplementary Table 2.** A total of 106 genes in the PPI network considered as hubs.

| **Gene name** | **Degree** | **Betweenness** |
| --- | --- | --- |
| HRAS | 119 | 0.069268989 |
| CDK1 | 95 | 0.020525163 |
| MAPK3 | 94 | 0.037301872 |
| ERBB2 | 88 | 0.041252237 |
| AURKB | 87 | 0.017148566 |
| COL1A1 | 85 | 0.022081069 |
| CYCS | 82 | 0.035804681 |
| KIF11 | 80 | 0.010903706 |
| BRCA1 | 79 | 0.020175658 |
| CCNA2 | 78 | 0.006947749 |
| CAT | 75 | 0.0249146 |
| RAD51 | 75 | 0.012875949 |
| RAC1 | 71 | 0.029317153 |
| RRM2 | 70 | 0.008593498 |
| CENPE | 70 | 0.006803541 |
| ANLN | 68 | 0.013782186 |
| COL1A2 | 66 | 0.008366615 |
| BIRC5 | 66 | 0.006104508 |
| TPI1 | 64 | 0.010910305 |
| EXO1 | 64 | 0.005062973 |
| COL5A1 | 63 | 0.00792567 |
| CDC25A | 62 | 0.005145472 |
| SOX2 | 61 | 0.026312526 |
| FOXM1 | 61 | 0.005918559 |
| DTL | 61 | 0.005093317 |
| TRIP13 | 60 | 0.006503583 |
| ENO1 | 58 | 0.014993432 |
| GOT2 | 57 | 0.009971852 |
| FYN | 56 | 0.017968793 |
| TK1 | 56 | 0.008781771 |
| ACO2 | 56 | 0.008542405 |
| MDH2 | 56 | 0.007399942 |
| POSTN | 54 | 0.005640934 |
| PDGFRB | 53 | 0.014781422 |
| CS | 53 | 0.006096533 |
| YWHAZ | 51 | 0.012011786 |
| ECT2 | 50 | 0.006449625 |
| MAPT | 49 | 0.016550376 |
| COL6A1 | 49 | 0.005264148 |
| RAC2 | 48 | 0.010902097 |
| ATP5A1 | 48 | 0.008264238 |
| CYC1 | 48 | 0.007149878 |
| COL4A1 | 48 | 0.006638627 |
| PRKCA | 46 | 0.01396252 |
| APOE | 45 | 0.016964882 |
| DLG4 | 45 | 0.014207983 |
| PPP2CA | 45 | 0.009401953 |
| MMP14 | 45 | 0.008959473 |
| YWHAB | 44 | 0.010841959 |
| PGK1 | 44 | 0.005666219 |
| EZR | 43 | 0.01507245 |
| FASN | 43 | 0.013556492 |
| TXN | 43 | 0.012698024 |
| RUNX2 | 43 | 0.010804088 |
| TALDO1 | 43 | 0.006294611 |
| TGFB3 | 41 | 0.009734241 |
| VDAC1 | 41 | 0.006369264 |
| SNAI1 | 40 | 0.010422104 |
| HSPG2 | 40 | 0.006578166 |
| NDUFA9 | 40 | 0.00630584 |
| SERPINH1 | 38 | 0.006940163 |
| IRS1 | 38 | 0.006807083 |
| IDH2 | 38 | 0.006343054 |
| OCLN | 36 | 0.01129048 |
| VCAN | 36 | 0.007129831 |
| PPP1CB | 35 | 0.013582908 |
| LMNA | 35 | 0.0086201 |
| HSPB1 | 35 | 0.007870615 |
| LEF1 | 35 | 0.007621994 |
| SYK | 35 | 0.007504681 |
| HMGCR | 35 | 0.005871575 |
| FGFR1 | 35 | 0.005145382 |
| ITGA1 | 34 | 0.006990643 |
| MLLT4 | 33 | 0.008425134 |
| GSTP1 | 33 | 0.006700857 |
| SNAP25 | 32 | 0.010904677 |
| KRT14 | 32 | 0.010313167 |
| LGALS3 | 32 | 0.007809553 |
| LDLR | 32 | 0.007545664 |
| P4HA1 | 32 | 0.006714828 |
| FGFR3 | 32 | 0.006085743 |
| MAPK9 | 31 | 0.008521958 |
| PLCB1 | 31 | 0.008218425 |
| GNB3 | 31 | 0.006733097 |
| DSP | 30 | 0.00682035 |
| SLBP | 30 | 0.006324139 |
| KRT5 | 30 | 0.005890489 |
| MVK | 30 | 0.005026545 |
| PARD3 | 29 | 0.009517001 |
| CACNA1C | 29 | 0.006839419 |
| JUP | 28 | 0.008062768 |
| NR3C1 | 28 | 0.007731143 |
| PPL | 28 | 0.007592727 |
| CEBPA | 28 | 0.007432799 |
| FKBP10 | 28 | 0.005822996 |
| ANXA1 | 27 | 0.007519516 |
| ADRB2 | 27 | 0.005052912 |
| SIRT2 | 26 | 0.008907165 |
| STX1A | 26 | 0.008681713 |
| MBP | 26 | 0.008282631 |
| IGF2 | 26 | 0.007920007 |
| MYH10 | 26 | 0.007468766 |
| GLI3 | 26 | 0.005299369 |
| PDGFA | 26 | 0.004971763 |
| PAFAH1B1 | 25 | 0.009675687 |
| IL18 | 25 | 0.005674206 |

PPI, protein-protein interaction.

**Supplementary Table 3.** Signaling pathways significantly deregulated in primary ameloblastoma compared to the normal gingiva.

| **Cluster No.** | **Pathway identifier** | **Pathway name** | **# Entities** | **FDR** |
| --- | --- | --- | --- | --- |
| 1 | R-HSA-1640170 | Cell Cycle | 46 | 5.66E-15 |
|  | R-HSA-69278 | Cell Cycle, Mitotic | 41 | 5.66E-15 |
|  | R-HSA-69620 | Cell Cycle Checkpoints | 23 | 5.66E-15 |
|  | R-HSA-453279 | Mitotic G1 phase and G1/S transition | 19 | 5.66E-15 |
|  | R-HSA-69206 | G1/S Transition | 16 | 5.66E-15 |
|  | R-HSA-69205 | G1/S-Specific Transcription | 11 | 9.33E-15 |
|  | R-HSA-2500257 | Resolution of Sister Chromatid Cohesion | 14 | 1.12E-13 |
|  | R-HSA-68877 | Mitotic Prometaphase | 15 | 2.31E-12 |
|  | R-HSA-156711 | Polo-like kinase mediated events | 8 | 8.42E-12 |
|  | R-HSA-141424 | Amplification of signal from the kinetochores | 11 | 2.45E-11 |
|  | R-HSA-141444 | Amplification of signal from unattached kinetochores via a MAD2 inhibitory signal | 11 | 2.45E-11 |
|  | R-HSA-69618 | Mitotic Spindle Checkpoint | 11 | 1.31E-10 |
|  | R-HSA-68882 | Mitotic Anaphase | 14 | 2.26E-10 |
|  | R-HSA-2555396 | Mitotic Metaphase and Anaphase | 14 | 2.26E-10 |
|  | R-HSA-9648025 | EML4 and NUDC in mitotic spindle formation | 11 | 2.52E-10 |
|  | R-HSA-1538133 | G0 and Early G1 | 8 | 2.52E-10 |
|  | R-HSA-69275 | G2/M Transition | 13 | 3.64E-10 |
|  | R-HSA-453274 | Mitotic G2-G2/M phases | 13 | 3.81E-10 |
|  | R-HSA-69242 | S Phase | 12 | 7.71E-10 |
|  | R-HSA-5663220 | RHO GTPases Activate Formins | 11 | 1.66E-09 |
|  | R-HSA-2467813 | Separation of Sister Chromatids | 12 | 1.76E-09 |
|  | R-HSA-69481 | G2/M Checkpoints | 11 | 2.14E-09 |
|  | R-HSA-69273 | Cyclin A/B1/B2 associated events during G2/M transition | 7 | 2.63E-09 |
|  | R-HSA-68886 | M Phase | 15 | 9.22E-09 |
|  | R-HSA-69239 | Synthesis of DNA | 9 | 1.39E-07 |
|  | R-HSA-73886 | Chromosome Maintenance | 9 | 1.91E-07 |
|  | R-HSA-69306 | DNA Replication | 9 | 2.43E-07 |
|  | R-HSA-69190 | DNA strand elongation | 6 | 2.65E-07 |
|  | R-HSA-176187 | Activation of ATR in response to replication stress | 6 | 3.09E-07 |
|  | R-HSA-195258 | RHO GTPase Effectors | 12 | 3.47E-07 |
|  | R-HSA-3700989 | Transcriptional Regulation by TP53 | 14 | 4.24E-07 |
|  | R-HSA-8953750 | Transcriptional Regulation by E2F6 | 6 | 7.09E-07 |
|  | R-HSA-6804756 | Regulation of TP53 Activity through Phosphorylation | 7 | 2.31E-06 |
|  | R-HSA-176974 | Unwinding of DNA | 4 | 2.31E-06 |
|  | R-HSA-5685938 | HDR through Single Strand Annealing (SSA) | 5 | 8.11E-06 |
|  | R-HSA-5693616 | Presynaptic phase of homologous DNA pairing and strand exchange | 5 | 1.03E-05 |
|  | R-HSA-5633007 | Regulation of TP53 Activity | 8 | 1.30E-05 |
|  | R-HSA-5693567 | HDR through Homologous Recombination (HRR) or Single Strand Annealing (SSA) | 7 | 1.34E-05 |
|  | R-HSA-5693579 | Homologous DNA Pairing and Strand Exchange | 5 | 1.44E-05 |
|  | R-HSA-1362300 | Transcription of E2F targets under negative control by p107 (RBL1) and p130 (RBL2) in complex with HDAC1 | 4 | 1.44E-05 |
|  | R-HSA-5693538 | Homology Directed Repair | 7 | 1.52E-05 |
|  | R-HSA-4615885 | SUMOylation of DNA replication proteins | 5 | 2.25E-05 |
|  | R-HSA-5693607 | Processing of DNA double-strand break ends | 6 | 2.41E-05 |
|  | R-HSA-194315 | Signaling by Rho GTPases | 14 | 2.60E-05 |
|  | R-HSA-174143 | APC/C-mediated degradation of cell cycle proteins | 6 | 2.60E-05 |
|  | R-HSA-453276 | Regulation of mitotic cell cycle | 6 | 2.60E-05 |
|  | R-HSA-774815 | Nucleosome assembly | 5 | 2.60E-05 |
|  | R-HSA-606279 | Deposition of new CENPA-containing nucleosomes at the centromere | 5 | 2.60E-05 |
|  | R-HSA-69478 | G2/M DNA replication checkpoint | 3 | 2.60E-05 |
|  | R-HSA-9716542 | Signaling by Rho GTPases, Miro GTPases and RHOBTB3 | 14 | 2.68E-05 |
|  | R-HSA-1362277 | Transcription of E2F targets under negative control by DREAM complex | 4 | 2.76E-05 |
|  | R-HSA-73894 | DNA Repair | 10 | 3.83E-05 |
|  | R-HSA-5693532 | DNA Double-Strand Break Repair | 7 | 6.33E-05 |
|  | R-HSA-5685942 | HDR through Homologous Recombination (HRR) | 5 | 1.03E-04 |
|  | R-HSA-68949 | Orc1 removal from chromatin | 5 | 1.10E-04 |
|  | R-HSA-68962 | Activation of the pre-replicative complex | 4 | 1.14E-04 |
|  | R-HSA-69473 | G2/M DNA damage checkpoint | 5 | 1.79E-04 |
|  | R-HSA-69052 | Switching of origins to a post-replicative state | 5 | 3.42E-04 |
|  | R-HSA-73893 | DNA Damage Bypass | 4 | 5.00E-04 |
|  | R-HSA-9675136 | Diseases of DNA Double-Strand Break Repair | 3 | 6.51E-04 |
|  | R-HSA-9701193 | Defective HDR through Homologous Recombination (HRR) due to PALB2 loss of function | 3 | 6.51E-04 |
|  | R-HSA-9704646 | Defective HDR through Homologous Recombination Repair (HRR) due to PALB2 loss of BRCA2/RAD51/RAD51C binding function | 3 | 6.51E-04 |
|  | R-HSA-9704331 | Defective HDR through Homologous Recombination Repair (HRR) due to PALB2 loss of BRCA1 binding function | 3 | 6.51E-04 |
|  | R-HSA-3108232 | SUMO E3 ligases SUMOylate target proteins | 6 | 7.25E-04 |
|  | R-HSA-212436 | Generic Transcription Pathway | 18 | 7.85E-04 |
|  | R-HSA-170145 | Phosphorylation of proteins involved in the G2/M transition by Cyclin A:Cdc2 complexes | 2 | 8.19E-04 |
|  | R-HSA-6791312 | TP53 Regulates Transcription of Cell Cycle Genes | 4 | 8.21E-04 |
|  | R-HSA-2990846 | SUMOylation | 6 | 9.35E-04 |
|  | R-HSA-180786 | Extension of Telomeres | 4 | 1.03E-03 |
|  | R-HSA-5693554 | Resolution of D-loop Structures through Synthesis-Dependent Strand Annealing (SDSA) | 3 | 1.25E-03 |
|  | R-HSA-110314 | Recognition of DNA damage by PCNA-containing replication complex | 3 | 1.25E-03 |
|  | R-HSA-68911 | G2 Phase | 2 | 1.60E-03 |
|  | R-HSA-73857 | RNA Polymerase II Transcription | 18 | 1.75E-03 |
|  | R-HSA-69002 | DNA Replication Pre-Initiation | 4 | 1.75E-03 |
|  | R-HSA-69202 | Cyclin E associated events during G1/S transition | 4 | 1.75E-03 |
|  | R-HSA-5656169 | Termination of translesion DNA synthesis | 3 | 1.75E-03 |
|  | R-HSA-5693568 | Resolution of D-loop Structures through Holliday Junction Intermediates | 3 | 1.75E-03 |
|  | R-HSA-5693537 | Resolution of D-Loop Structures | 3 | 1.75E-03 |
|  | R-HSA-9675135 | Diseases of DNA repair | 3 | 1.75E-03 |
|  | R-HSA-2980767 | Activation of NIMA Kinases NEK9, NEK6, NEK7 | 2 | 1.75E-03 |
|  | R-HSA-69656 | Cyclin A:Cdk2-associated events at S phase entry | 4 | 1.84E-03 |
|  | R-HSA-2565942 | Regulation of PLK1 Activity at G2/M Transition | 4 | 2.00E-03 |
|  | R-HSA-6804757 | Regulation of TP53 Degradation | 3 | 2.34E-03 |
|  | R-HSA-6806003 | Regulation of TP53 Expression and Degradation | 3 | 2.84E-03 |
|  | R-HSA-110313 | Translesion synthesis by Y family DNA polymerases bypasses lesions on DNA template | 3 | 3.20E-03 |
|  | R-HSA-157579 | Telomere Maintenance | 4 | 3.95E-03 |
|  | R-HSA-74160 | Gene expression (Transcription) | 18 | 4.21E-03 |
|  | R-HSA-2514853 | Condensation of Prometaphase Chromosomes | 2 | 4.77E-03 |
|  | R-HSA-69109 | Leading Strand Synthesis | 2 | 5.41E-03 |
|  | R-HSA-69091 | Polymerase switching | 2 | 5.41E-03 |
|  | R-HSA-912446 | Meiotic recombination | 3 | 5.46E-03 |
|  | R-HSA-162658 | Golgi Cisternae Pericentriolar Stack Reorganization | 2 | 6.09E-03 |
|  | R-HSA-110312 | Translesion synthesis by REV1 | 2 | 7.56E-03 |
|  | R-HSA-5656121 | Translesion synthesis by POLI | 2 | 8.35E-03 |
|  | R-HSA-6804116 | TP53 Regulates Transcription of Genes Involved in G1 Cell Cycle Arrest | 2 | 8.35E-03 |
|  | R-HSA-68867 | Assembly of the pre-replicative complex | 3 | 8.51E-03 |
|  | R-HSA-5655862 | Translesion synthesis by POLK | 2 | 9.18E-03 |
|  | R-HSA-6804114 | TP53 Regulates Transcription of Genes Involved in G2 Cell Cycle Arrest | 2 | 9.18E-03 |
|  | R-HSA-179419 | APC:Cdc20 mediated degradation of cell cycle proteins prior to satisfation of the cell cycle checkpoint | 3 | 9.72E-03 |
|  | R-HSA-8854518 | AURKA Activation by TPX2 | 3 | 9.72E-03 |
|  | R-HSA-176409 | APC/C:Cdc20 mediated degradation of mitotic proteins | 3 | 9.72E-03 |
|  | R-HSA-176814 | Activation of APC/C and APC/C:Cdc20 mediated degradation of mitotic proteins | 3 | 9.72E-03 |
|  | R-HSA-176408 | Regulation of APC/C activators between G1/S and early anaphase | 3 | 9.72E-03 |
|  | R-HSA-6796648 | TP53 Regulates Transcription of DNA Repair Genes | 3 | 9.72E-03 |
|  | R-HSA-1500620 | Meiosis | 3 | 9.72E-03 |
|  | R-HSA-5651801 | PCNA-Dependent Long Patch Base Excision Repair | 2 | 9.72E-03 |
|  | R-HSA-110320 | Translesion Synthesis by POLH | 2 | 9.72E-03 |
|  | R-HSA-69186 | Lagging Strand Synthesis | 2 | 9.72E-03 |
|  | R-HSA-5696397 | Gap-filling DNA repair synthesis and ligation in GG-NER | 2 | 9.72E-03 |
|  | R-HSA-2995383 | Initiation of Nuclear Envelope (NE) Reformation | 2 | 9.72E-03 |
|  | R-HSA-6803205 | TP53 regulates transcription of several additional cell death genes whose specific roles in p53-dependent apoptosis remain uncertain | 2 | 9.72E-03 |
|  | R-HSA-8862803 | Deregulated CDK5 triggers multiple neurodegenerative pathways in Alzheimer's disease models | 2 | 9.72E-03 |
|  | R-HSA-8863678 | Neurodegenerative Diseases | 2 | 9.72E-03 |
|  | R-HSA-174411 | Polymerase switching on the C-strand of the telomere | 2 | 9.72E-03 |
|  | R-HSA-110373 | Resolution of AP sites via the multiple-nucleotide patch replacement pathway | 2 | 1.03E-02 |
|  | R-HSA-171319 | Telomere Extension By Telomerase | 2 | 1.16E-02 |
|  | R-HSA-5696400 | Dual Incision in GG-NER | 2 | 1.65E-02 |
|  | R-HSA-3301854 | Nuclear Pore Complex (NPC) Disassembly | 2 | 1.72E-02 |
|  | R-HSA-1474165 | Reproduction | 3 | 2.10E-02 |
|  | R-HSA-73933 | Resolution of Abasic Sites (AP sites) | 2 | 2.21E-02 |
|  | R-HSA-174417 | Telomere C-strand (Lagging Strand) Synthesis | 2 | 2.47E-02 |
|  | R-HSA-187577 | SCF(Skp2)-mediated degradation of p27/p21 | 2 | 3.53E-02 |
|  | R-HSA-2980766 | Nuclear Envelope Breakdown | 2 | 3.85E-02 |
|  | R-HSA-6782210 | Gap-filling DNA repair synthesis and ligation in TC-NER | 2 | 3.95E-02 |
|  | R-HSA-983189 | Kinesins | 2 | 4.17E-02 |
|  | R-HSA-6782135 | Dual incision in TC-NER | 2 | 4.17E-02 |
|  | R-HSA-380284 | Loss of proteins required for interphase microtubule organization from the centrosome | 2 | 4.51E-02 |
|  | R-HSA-380259 | Loss of Nlp from mitotic centrosomes | 2 | 4.51E-02 |
|  | R-HSA-2559586 | DNA Damage/Telomere Stress Induced Senescence | 2 | 4.51E-02 |
|  | R-HSA-69615 | G1/S DNA Damage Checkpoints | 2 | 4.62E-02 |
|  | R-HSA-174184 | Cdc20:Phospho-APC/C mediated degradation of Cyclin A | 2 | 4.74E-02 |
|  | R-HSA-174178 | APC/C:Cdh1 mediated degradation of Cdc20 and other APC/C:Cdh1 targeted proteins in late mitosis/early G1 | 2 | 4.85E-02 |
|  | R-HSA-5688426 | Deubiquitination | 4 | 4.88E-02 |
|  | R-HSA-9013026 | RHOB GTPase cycle | 2 | 4.97E-02 |
|  | R-HSA-69017 | CDK-mediated phosphorylation and removal of Cdc6 | 2 | 4.97E-02 |
| 2 | R-HSA-1428517 | The citric acid (TCA) cycle and respiratory electron transport | 17 | 1.05E-14 |
|  | R-HSA-2022090 | Assembly of collagen fibrils and other multimeric structures | 12 | 1.05E-14 |
|  | R-HSA-3000178 | ECM proteoglycans | 12 | 5.60E-14 |
|  | R-HSA-1474244 | Extracellular matrix organization | 18 | 5.74E-14 |
|  | R-HSA-8948216 | Collagen chain trimerization | 10 | 1.90E-13 |
|  | R-HSA-1442490 | Collagen degradation | 11 | 2.86E-13 |
|  | R-HSA-1474290 | Collagen formation | 12 | 6.09E-13 |
|  | R-HSA-1650814 | Collagen biosynthesis and modifying enzymes | 10 | 2.47E-11 |
|  | R-HSA-163200 | Respiratory electron transport, ATP synthesis by chemiosmotic coupling, and heat production by uncoupling proteins. | 12 | 4.18E-11 |
|  | R-HSA-216083 | Integrin cell surface interactions | 10 | 6.81E-11 |
|  | R-HSA-1474228 | Degradation of the extracellular matrix | 11 | 5.40E-10 |
|  | R-HSA-611105 | Respiratory electron transport | 10 | 1.15E-09 |
|  | R-HSA-3000171 | Non-integrin membrane-ECM interactions | 8 | 3.24E-09 |
|  | R-HSA-186797 | Signaling by PDGF | 8 | 8.79E-09 |
|  | R-HSA-419037 | NCAM1 interactions | 7 | 1.05E-08 |
|  | R-HSA-2243919 | Crosslinking of collagen fibrils | 6 | 1.14E-08 |
|  | R-HSA-375165 | NCAM signaling for neurite out-growth | 7 | 2.29E-07 |
|  | R-HSA-71387 | Metabolism of carbohydrates | 13 | 4.53E-07 |
|  | R-HSA-70326 | Glucose metabolism | 8 | 1.38E-06 |
|  | R-HSA-8874081 | MET activates PTK2 signaling | 5 | 2.49E-06 |
|  | R-HSA-1430728 | Metabolism | 34 | 2.68E-06 |
|  | R-HSA-70171 | Glycolysis | 7 | 3.46E-06 |
|  | R-HSA-2214320 | Anchoring fibril formation | 4 | 4.51E-06 |
|  | R-HSA-8875878 | MET promotes cell motility | 5 | 1.02E-05 |
|  | R-HSA-71403 | Citric acid cycle (TCA cycle) | 5 | 1.70E-05 |
|  | R-HSA-3000170 | Syndecan interactions | 4 | 5.28E-05 |
|  | R-HSA-70263 | Gluconeogenesis | 5 | 6.49E-05 |
|  | R-HSA-6806834 | Signaling by MET | 5 | 2.18E-04 |
|  | R-HSA-1566948 | Elastic fibre formation | 4 | 2.71E-04 |
|  | R-HSA-9006934 | Signaling by Receptor Tyrosine Kinases | 11 | 2.81E-04 |
|  | R-HSA-71406 | Pyruvate metabolism and Citric Acid (TCA) cycle | 5 | 2.88E-04 |
|  | R-HSA-3000480 | Scavenging by Class A Receptors | 4 | 2.88E-04 |
|  | R-HSA-9636667 | Manipulation of host energy metabolism | 2 | 3.86E-04 |
|  | R-HSA-6799198 | Complex I biogenesis | 4 | 5.88E-04 |
|  | R-HSA-1592230 | Mitochondrial biogenesis | 5 | 1.04E-03 |
|  | R-HSA-3000157 | Laminin interactions | 3 | 1.59E-03 |
|  | R-HSA-71336 | Pentose phosphate pathway | 3 | 4.97E-03 |
|  | R-HSA-430116 | GP1b-IX-V activation signalling | 2 | 6.03E-03 |
|  | R-HSA-9711123 | Cellular response to chemical stress | 5 | 7.33E-03 |
|  | R-HSA-5628897 | TP53 Regulates Metabolic Genes | 4 | 7.94E-03 |
|  | R-HSA-75892 | Platelet Adhesion to exposed collagen | 2 | 8.49E-03 |
|  | R-HSA-9029558 | NR1H2 & NR1H3 regulate gene expression linked to lipogenesis | 2 | 9.56E-03 |
|  | R-HSA-422475 | Axon guidance | 8 | 1.19E-02 |
|  | R-HSA-1268020 | Mitochondrial protein import | 3 | 1.25E-02 |
|  | R-HSA-9675108 | Nervous system development | 8 | 1.62E-02 |
|  | R-HSA-9707564 | Cytoprotection by HMOX1 | 4 | 1.62E-02 |
|  | R-HSA-2173782 | Binding and Uptake of Ligands by Scavenger Receptors | 4 | 1.62E-02 |
|  | R-HSA-163210 | Formation of ATP by chemiosmotic coupling | 2 | 1.62E-02 |
|  | R-HSA-9609507 | Protein localization | 4 | 1.72E-02 |
|  | R-HSA-8949215 | Mitochondrial calcium ion transport | 2 | 1.76E-02 |
|  | R-HSA-2151201 | Transcriptional activation of mitochondrial biogenesis | 3 | 1.84E-02 |
|  | R-HSA-8949613 | Cristae formation | 2 | 2.30E-02 |
|  | R-HSA-8940973 | RUNX2 regulates osteoblast differentiation | 2 | 2.74E-02 |
|  | R-HSA-2129379 | Molecules associated with elastic fibres | 2 | 3.38E-02 |
|  | R-HSA-1852241 | Organelle biogenesis and maintenance | 5 | 4.03E-02 |
|  | R-HSA-114604 | GPVI-mediated activation cascade | 2 | 4.28E-02 |
|  | R-HSA-8941326 | RUNX2 regulates bone development | 2 | 4.28E-02 |
|  | R-HSA-9615017 | FOXO-mediated transcription of oxidative stress, metabolic and neuronal genes | 2 | 4.52E-02 |
|  | R-HSA-9637690 | Response of Mtb to phagocytosis | 2 | 4.52E-02 |
|  | R-HSA-76009 | Platelet Aggregation (Plug Formation) | 2 | 4.52E-02 |
|  | R-HSA-3560782 | Diseases associated with glycosaminoglycan metabolism | 2 | 4.52E-02 |
| 3 | R-HSA-8957322 | Metabolism of steroids | 25 | 2.22E-15 |
|  | R-HSA-1474244 | Extracellular matrix organization | 22 | 2.22E-15 |
|  | R-HSA-2426168 | Activation of gene expression by SREBF (SREBP) | 18 | 2.22E-15 |
|  | R-HSA-1655829 | Regulation of cholesterol biosynthesis by SREBP (SREBF) | 18 | 2.22E-15 |
|  | R-HSA-191273 | Cholesterol biosynthesis | 16 | 2.22E-15 |
|  | R-HSA-1650814 | Collagen biosynthesis and modifying enzymes | 12 | 3.55E-15 |
|  | R-HSA-1474290 | Collagen formation | 12 | 1.04E-13 |
|  | R-HSA-556833 | Metabolism of lipids | 27 | 3.54E-12 |
|  | R-HSA-3000157 | Laminin interactions | 7 | 4.66E-10 |
|  | R-HSA-1474228 | Degradation of the extracellular matrix | 9 | 5.76E-08 |
|  | R-HSA-9619665 | EGR2 and SOX10-mediated initiation of Schwann cell myelination | 6 | 9.69E-08 |
|  | R-HSA-216083 | Integrin cell surface interactions | 7 | 3.64E-07 |
|  | R-HSA-3000178 | ECM proteoglycans | 6 | 4.63E-06 |
|  | R-HSA-8948216 | Collagen chain trimerization | 5 | 5.96E-06 |
|  | R-HSA-1989781 | PPARA activates gene expression | 7 | 3.03E-05 |
|  | R-HSA-400206 | Regulation of lipid metabolism by PPARalpha | 7 | 3.26E-05 |
|  | R-HSA-2022090 | Assembly of collagen fibrils and other multimeric structures | 5 | 3.26E-05 |
|  | R-HSA-8874081 | MET activates PTK2 signaling | 4 | 3.93E-05 |
|  | R-HSA-8875878 | MET promotes cell motility | 4 | 1.48E-04 |
|  | R-HSA-1430728 | Metabolism | 28 | 2.42E-04 |
|  | R-HSA-3000171 | Non-integrin membrane-ECM interactions | 4 | 3.82E-04 |
|  | R-HSA-1442490 | Collagen degradation | 4 | 6.12E-04 |
|  | R-HSA-6806834 | Signaling by MET | 4 | 1.53E-03 |
|  | R-HSA-8957275 | Post-translational protein phosphorylation | 4 | 3.40E-03 |
|  | R-HSA-6807047 | Cholesterol biosynthesis via desmosterol | 2 | 4.14E-03 |
|  | R-HSA-6807062 | Cholesterol biosynthesis via lathosterol | 2 | 4.14E-03 |
|  | R-HSA-381426 | Regulation of Insulin-like Growth Factor (IGF) transport and uptake by Insulin-like Growth Factor Binding Proteins (IGFBPs) | 4 | 4.46E-03 |
|  | R-HSA-77111 | Synthesis of Ketone Bodies | 2 | 1.19E-02 |
|  | R-HSA-74182 | Ketone body metabolism | 2 | 1.60E-02 |
|  | R-HSA-8964038 | LDL clearance | 2 | 1.60E-02 |
|  | R-HSA-9675108 | Nervous system development | 7 | 2.75E-02 |
|  | R-HSA-2129379 | Molecules associated with elastic fibres | 2 | 2.75E-02 |
|  | R-HSA-8964043 | Plasma lipoprotein clearance | 2 | 2.75E-02 |
|  | R-HSA-1566948 | Elastic fibre formation | 2 | 2.75E-02 |
|  | R-HSA-373760 | L1CAM interactions | 3 | 2.78E-02 |
|  | R-HSA-1251985 | Nuclear signaling by ERBB4 | 2 | 2.87E-02 |
| 5 | R-HSA-1430728 | Metabolism | 11 | 4.33E-06 |
|  | R-HSA-71406 | Pyruvate metabolism and Citric Acid (TCA) cycle | 4 | 6.07E-06 |
|  | R-HSA-1428517 | The citric acid (TCA) cycle and respiratory electron transport | 5 | 7.39E-06 |
|  | R-HSA-1268020 | Mitochondrial protein import | 4 | 7.39E-06 |
|  | R-HSA-71403 | Citric acid cycle (TCA cycle) | 3 | 1.48E-05 |
|  | R-HSA-8964540 | Alanine metabolism | 2 | 1.57E-05 |
|  | R-HSA-70263 | Gluconeogenesis | 3 | 3.80E-05 |
|  | R-HSA-9609507 | Protein localization | 4 | 1.14E-04 |
|  | R-HSA-70326 | Glucose metabolism | 3 | 6.26E-04 |
|  | R-HSA-71387 | Metabolism of carbohydrates | 4 | 9.37E-04 |
|  | R-HSA-70171 | Glycolysis | 2 | 8.56E-03 |
| 6 | R-HSA-400042 | Adrenaline,noradrenaline inhibits insulin secretion | 4 | 2.53E-05 |
|  | R-HSA-156590 | Glutathione conjugation | 4 | 1.34E-04 |
|  | R-HSA-8964315 | G beta:gamma signalling through BTK | 3 | 1.34E-04 |
|  | R-HSA-8964616 | G beta:gamma signalling through CDC42 | 3 | 1.34E-04 |
|  | R-HSA-418217 | G beta:gamma signalling through PLC beta | 3 | 1.34E-04 |
|  | R-HSA-392851 | Prostacyclin signalling through prostacyclin receptor | 3 | 1.34E-04 |
|  | R-HSA-500657 | Presynaptic function of Kainate receptors | 3 | 1.34E-04 |
|  | R-HSA-1430728 | Metabolism | 15 | 1.38E-04 |
|  | R-HSA-392170 | ADP signalling through P2Y purinoceptor 12 | 3 | 1.38E-04 |
|  | R-HSA-202040 | G-protein activation | 3 | 1.38E-04 |
|  | R-HSA-428930 | Thromboxane signalling through TP receptor | 3 | 1.38E-04 |
|  | R-HSA-392451 | G beta:gamma signalling through PI3Kgamma | 3 | 1.38E-04 |
|  | R-HSA-418592 | ADP signalling through P2Y purinoceptor 1 | 3 | 1.38E-04 |
|  | R-HSA-1296059 | G protein gated Potassium channels | 3 | 1.38E-04 |
|  | R-HSA-997272 | Inhibition of voltage gated Ca2+ channels via Gbeta/gamma subunits | 3 | 1.38E-04 |
|  | R-HSA-1296041 | Activation of G protein gated Potassium channels | 3 | 1.38E-04 |
|  | R-HSA-422356 | Regulation of insulin secretion | 4 | 1.53E-04 |
|  | R-HSA-451326 | Activation of kainate receptors upon glutamate binding | 3 | 1.62E-04 |
|  | R-HSA-456926 | Thrombin signalling through proteinase activated receptors (PARs) | 3 | 1.62E-04 |
|  | R-HSA-420092 | Glucagon-type ligand receptors | 3 | 1.62E-04 |
|  | R-HSA-418346 | Platelet homeostasis | 4 | 1.80E-04 |
|  | R-HSA-1296065 | Inwardly rectifying K+ channels | 3 | 1.83E-04 |
|  | R-HSA-397795 | G-protein beta:gamma signalling | 3 | 1.87E-04 |
|  | R-HSA-392518 | Signal amplification | 3 | 1.87E-04 |
|  | R-HSA-163359 | Glucagon signaling in metabolic regulation | 3 | 1.87E-04 |
|  | R-HSA-6814122 | Cooperation of PDCL (PhLP1) and TRiC/CCT in G-protein beta folding | 3 | 2.01E-04 |
|  | R-HSA-991365 | Activation of GABAB receptors | 3 | 2.92E-04 |
|  | R-HSA-977444 | GABA B receptor activation | 3 | 2.92E-04 |
|  | R-HSA-381676 | Glucagon-like Peptide-1 (GLP1) regulates insulin secretion | 3 | 2.92E-04 |
|  | R-HSA-163685 | Integration of energy metabolism | 4 | 3.10E-04 |
|  | R-HSA-432040 | Vasopressin regulates renal water homeostasis via Aquaporins | 3 | 3.48E-04 |
|  | R-HSA-211859 | Biological oxidations | 6 | 4.62E-04 |
|  | R-HSA-418597 | G alpha (z) signalling events | 3 | 4.87E-04 |
|  | R-HSA-977443 | GABA receptor activation | 3 | 6.11E-04 |
|  | R-HSA-445717 | Aquaporin-mediated transport | 3 | 6.38E-04 |
|  | R-HSA-4086398 | Ca2+ pathway | 3 | 1.07E-03 |
|  | R-HSA-416482 | G alpha (12/13) signalling events | 3 | 1.23E-03 |
|  | R-HSA-390466 | Chaperonin-mediated protein folding | 3 | 1.40E-03 |
|  | R-HSA-373080 | Class B/2 (Secretin family receptors) | 3 | 1.53E-03 |
|  | R-HSA-391251 | Protein folding | 3 | 1.67E-03 |
|  | R-HSA-156580 | Phase II - Conjugation of compounds | 4 | 1.89E-03 |
|  | R-HSA-1296071 | Potassium Channels | 3 | 1.92E-03 |
|  | R-HSA-9009391 | Extra-nuclear estrogen signaling | 3 | 2.08E-03 |
|  | R-HSA-111885 | Opioid Signalling | 3 | 2.19E-03 |
|  | R-HSA-2559580 | Oxidative Stress Induced Senescence | 3 | 2.31E-03 |
|  | R-HSA-195721 | Signaling by WNT | 4 | 4.50E-03 |
|  | R-HSA-9660821 | ADORA2B mediated anti-inflammatory cytokines production | 3 | 4.50E-03 |
|  | R-HSA-5673000 | RAF activation | 2 | 4.83E-03 |
|  | R-HSA-3858494 | Beta-catenin independent WNT signaling | 3 | 5.09E-03 |
|  | R-HSA-418555 | G alpha (s) signalling events | 3 | 5.63E-03 |
|  | R-HSA-9658195 | Leishmania infection | 4 | 7.45E-03 |
|  | R-HSA-2559583 | Cellular Senescence | 3 | 8.62E-03 |
|  | R-HSA-112314 | Neurotransmitter receptors and postsynaptic signal transmission | 3 | 1.29E-02 |
|  | R-HSA-8939211 | ESR-mediated signaling | 3 | 1.72E-02 |
|  | R-HSA-1660661 | Sphingolipid de novo biosynthesis | 2 | 1.78E-02 |
|  | R-HSA-416476 | G alpha (q) signalling events | 3 | 2.26E-02 |
|  | R-HSA-76002 | Platelet activation, signaling and aggregation | 3 | 2.44E-02 |
|  | R-HSA-9662851 | Anti-inflammatory response favouring Leishmania parasite infection | 3 | 2.58E-02 |
|  | R-HSA-9664433 | Leishmania parasite growth and survival | 3 | 2.58E-02 |
|  | R-HSA-112315 | Transmission across Chemical Synapses | 3 | 3.15E-02 |
|  | R-HSA-9006931 | Signaling by Nuclear Receptors | 3 | 3.43E-02 |
|  | R-HSA-109582 | Hemostasis | 4 | 3.83E-02 |
|  | R-HSA-418594 | G alpha (i) signalling events | 3 | 3.83E-02 |
|  | R-HSA-112316 | Neuronal System | 3 | 3.83E-02 |
|  | R-HSA-428157 | Sphingolipid metabolism | 2 | 3.83E-02 |
|  | R-HSA-1643685 | Disease | 7 | 4.79E-02 |
| 7 | R-HSA-428157 | Sphingolipid metabolism | 7 | 2.75E-07 |
|  | R-HSA-1660661 | Sphingolipid de novo biosynthesis | 5 | 7.02E-06 |
|  | R-HSA-2022870 | Chondroitin sulfate biosynthesis | 3 | 1.13E-03 |
|  | R-HSA-1793185 | Chondroitin sulfate/dermatan sulfate metabolism | 3 | 1.26E-02 |
|  | R-HSA-2173791 | TGF-beta receptor signaling in EMT (epithelial to mesenchymal transition) | 2 | 3.15E-02 |
|  | R-HSA-2995383 | Initiation of Nuclear Envelope (NE) Reformation | 2 | 3.30E-02 |
|  | R-HSA-2028269 | Signaling by Hippo | 2 | 3.30E-02 |
|  | R-HSA-556833 | Metabolism of lipids | 7 | 3.30E-02 |
|  | R-HSA-1430728 | Metabolism | 12 | 3.36E-02 |
|  | R-HSA-69273 | Cyclin A/B1/B2 associated events during G2/M transition | 2 | 3.79E-02 |
| 8 | R-HSA-2219530 | Constitutive Signaling by Aberrant PI3K in Cancer | 7 | 2.69E-09 |
|  | R-HSA-2219528 | PI3K/AKT Signaling in Cancer | 7 | 6.95E-09 |
|  | R-HSA-6811558 | PI5P, PP2A and IER3 Regulate PI3K/AKT Signaling | 7 | 6.95E-09 |
|  | R-HSA-199418 | Negative regulation of the PI3K/AKT network | 7 | 7.85E-09 |
|  | R-HSA-5663202 | Diseases of signal transduction by growth factor receptors and second messengers | 8 | 1.70E-06 |
|  | R-HSA-1257604 | PIP3 activates AKT signaling | 7 | 1.70E-06 |
|  | R-HSA-9006925 | Intracellular signaling by second messengers | 7 | 3.67E-06 |
|  | R-HSA-9006934 | Signaling by Receptor Tyrosine Kinases | 8 | 6.26E-06 |
|  | R-HSA-5655302 | Signaling by FGFR1 in disease | 4 | 6.84E-06 |
|  | R-HSA-6809371 | Formation of the cornified envelope | 5 | 9.52E-06 |
|  | R-HSA-109704 | PI3K Cascade | 4 | 9.63E-06 |
|  | R-HSA-112399 | IRS-mediated signalling | 4 | 1.28E-05 |
|  | R-HSA-5673001 | RAF/MAP kinase cascade | 6 | 1.47E-05 |
|  | R-HSA-2428928 | IRS-related events triggered by IGF1R | 4 | 1.47E-05 |
|  | R-HSA-74751 | Insulin receptor signalling cascade | 4 | 1.47E-05 |
|  | R-HSA-2428924 | IGF1R signaling cascade | 4 | 1.47E-05 |
|  | R-HSA-2404192 | Signaling by Type 1 Insulin-like Growth Factor 1 Receptor (IGF1R) | 4 | 1.47E-05 |
|  | R-HSA-5684996 | MAPK1/MAPK3 signaling | 6 | 1.58E-05 |
|  | R-HSA-1226099 | Signaling by FGFR in disease | 4 | 2.03E-05 |
|  | R-HSA-5683057 | MAPK family signaling cascades | 6 | 3.15E-05 |
|  | R-HSA-74752 | Signaling by Insulin receptor | 4 | 3.42E-05 |
|  | R-HSA-5654689 | PI-3K cascade:FGFR1 | 3 | 3.42E-05 |
|  | R-HSA-6805567 | Keratinization | 5 | 3.94E-05 |
|  | R-HSA-1839124 | FGFR1 mutant receptor activation | 3 | 9.16E-05 |
|  | R-HSA-5654687 | Downstream signaling of activated FGFR1 | 3 | 9.52E-05 |
|  | R-HSA-1839120 | Signaling by FGFR1 amplification mutants | 2 | 9.52E-05 |
|  | R-HSA-5654736 | Signaling by FGFR1 | 3 | 3.01E-04 |
|  | R-HSA-351906 | Apoptotic cleavage of cell adhesion proteins | 2 | 4.34E-04 |
|  | R-HSA-1839122 | Signaling by activated point mutants of FGFR1 | 2 | 8.05E-04 |
|  | R-HSA-190236 | Signaling by FGFR | 3 | 1.38E-03 |
|  | R-HSA-190242 | FGFR1 ligand binding and activation | 2 | 1.38E-03 |
|  | R-HSA-5654219 | Phospholipase C-mediated cascade: FGFR1 | 2 | 1.38E-03 |
|  | R-HSA-1963642 | PI3K events in ERBB2 signaling | 2 | 1.38E-03 |
|  | R-HSA-1266738 | Developmental Biology | 7 | 1.99E-03 |
|  | R-HSA-6803205 | TP53 regulates transcription of several additional cell death genes whose specific roles in p53-dependent apoptosis remain uncertain | 2 | 2.22E-03 |
|  | R-HSA-5654688 | SHC-mediated cascade:FGFR1 | 2 | 2.55E-03 |
|  | R-HSA-5654693 | FRS-mediated FGFR1 signaling | 2 | 2.72E-03 |
|  | R-HSA-9664565 | Signaling by ERBB2 KD Mutants | 2 | 2.89E-03 |
|  | R-HSA-1227990 | Signaling by ERBB2 in Cancer | 2 | 2.89E-03 |
|  | R-HSA-186763 | Downstream signal transduction | 2 | 2.89E-03 |
|  | R-HSA-111465 | Apoptotic cleavage of cellular proteins | 2 | 3.05E-03 |
|  | R-HSA-5654726 | Negative regulation of FGFR1 signaling | 2 | 3.21E-03 |
|  | R-HSA-1643685 | Disease | 9 | 3.39E-03 |
|  | R-HSA-162582 | Signal Transduction | 10 | 3.46E-03 |
|  | R-HSA-75153 | Apoptotic execution phase | 2 | 6.08E-03 |
|  | R-HSA-1227986 | Signaling by ERBB2 | 2 | 9.55E-03 |
|  | R-HSA-186797 | Signaling by PDGF | 2 | 1.01E-02 |
|  | R-HSA-5633008 | TP53 Regulates Transcription of Cell Death Genes | 2 | 1.41E-02 |
|  | R-HSA-6798695 | Neutrophil degranulation | 3 | 3.05E-02 |
|  | R-HSA-166520 | Signaling by NTRKs | 2 | 3.05E-02 |
|  | R-HSA-109581 | Apoptosis | 2 | 3.05E-02 |
|  | R-HSA-5357801 | Programmed Cell Death | 2 | 3.44E-02 |
| 9 | R-HSA-9639288 | Amino acids regulate mTORC1 | 5 | 5.84E-08 |
|  | R-HSA-5173214 | O-glycosylation of TSR domain-containing proteins | 4 | 1.12E-06 |
|  | R-HSA-9711097 | Cellular response to starvation | 5 | 3.53E-06 |
|  | R-HSA-5173105 | O-linked glycosylation | 4 | 3.79E-05 |
|  | R-HSA-5083635 | Defective B3GALTL causes Peters-plus syndrome (PpS) | 3 | 5.69E-05 |
|  | R-HSA-3906995 | Diseases associated with O-glycosylation of proteins | 3 | 3.28E-04 |
|  | R-HSA-2262752 | Cellular responses to stress | 5 | 1.51E-03 |
|  | R-HSA-8953897 | Cellular responses to stimuli | 5 | 1.51E-03 |
|  | R-HSA-3781865 | Diseases of glycosylation | 3 | 1.51E-03 |
|  | R-HSA-166208 | mTORC1-mediated signalling | 2 | 1.51E-03 |
|  | R-HSA-380972 | Energy dependent regulation of mTOR by LKB1-AMPK | 2 | 1.51E-03 |
|  | R-HSA-165159 | MTOR signalling | 2 | 2.69E-03 |
|  | R-HSA-5668914 | Diseases of metabolism | 3 | 4.30E-03 |
|  | R-HSA-8943724 | Regulation of PTEN gene transcription | 2 | 4.30E-03 |
|  | R-HSA-5628897 | TP53 Regulates Metabolic Genes | 2 | 9.07E-03 |
|  | R-HSA-1632852 | Macroautophagy | 2 | 2.14E-02 |
|  | R-HSA-6807070 | PTEN Regulation | 2 | 2.20E-02 |
|  | R-HSA-9612973 | Autophagy | 2 | 2.62E-02 |
|  | R-HSA-597592 | Post-translational protein modification | 4 | 3.82E-02 |
|  | R-HSA-1257604 | PIP3 activates AKT signaling | 2 | 3.82E-02 |
|  | R-HSA-1474244 | Extracellular matrix organization | 2 | 3.82E-02 |
|  | R-HSA-9006925 | Intracellular signaling by second messengers | 2 | 3.82E-02 |
|  | R-HSA-3700989 | Transcriptional Regulation by TP53 | 2 | 4.55E-02 |
| 14 | R-HSA-9609507 | Protein localization | 8 | 2.68E-10 |
|  | R-HSA-9033241 | Peroxisomal protein import | 6 | 2.36E-09 |
|  | R-HSA-556833 | Metabolism of lipids | 10 | 8.50E-06 |
|  | R-HSA-390918 | Peroxisomal lipid metabolism | 4 | 4.03E-05 |
|  | R-HSA-8978868 | Fatty acid metabolism | 6 | 5.24E-05 |
|  | R-HSA-389887 | Beta-oxidation of pristanoyl-CoA | 3 | 5.31E-05 |
|  | R-HSA-163560 | Triglyceride catabolism | 3 | 1.18E-04 |
|  | R-HSA-8979227 | Triglyceride metabolism | 3 | 4.01E-04 |
|  | R-HSA-1430728 | Metabolism | 11 | 1.98E-03 |
|  | R-HSA-9029569 | NR1H3 & NR1H2 regulate gene expression linked to cholesterol transport and efflux | 2 | 1.42E-02 |
|  | R-HSA-193368 | Synthesis of bile acids and bile salts via 7alpha-hydroxycholesterol | 2 | 1.56E-02 |
|  | R-HSA-9024446 | NR1H2 and NR1H3-mediated signaling | 2 | 1.94E-02 |
|  | R-HSA-174824 | Plasma lipoprotein assembly, remodeling, and clearance | 2 | 2.56E-02 |
|  | R-HSA-192105 | Synthesis of bile acids and bile salts | 2 | 2.60E-02 |
|  | R-HSA-194068 | Bile acid and bile salt metabolism | 2 | 2.96E-02 |
|  | R-HSA-8957322 | Metabolism of steroids | 2 | 4.84E-02 |
| 15 | R-HSA-194315 | Signaling by Rho GTPases | 12 | 1.66E-06 |
|  | R-HSA-9716542 | Signaling by Rho GTPases, Miro GTPases and RHOBTB3 | 12 | 1.66E-06 |
|  | R-HSA-195258 | RHO GTPase Effectors | 9 | 1.66E-06 |
|  | R-HSA-3371497 | HSP90 chaperone cycle for steroid hormone receptors (SHR) in the presence of ligand | 6 | 1.66E-06 |
|  | R-HSA-5626467 | RHO GTPases activate IQGAPs | 5 | 1.66E-06 |
|  | R-HSA-983189 | Kinesins | 5 | 1.90E-05 |
|  | R-HSA-8856688 | Golgi-to-ER retrograde transport | 6 | 2.73E-05 |
|  | R-HSA-2132295 | MHC class II antigen presentation | 6 | 2.73E-05 |
|  | R-HSA-5663220 | RHO GTPases Activate Formins | 6 | 2.73E-05 |
|  | R-HSA-5625970 | RHO GTPases activate KTN1 | 3 | 7.22E-05 |
|  | R-HSA-983231 | Factors involved in megakaryocyte development and platelet production | 6 | 8.25E-05 |
|  | R-HSA-6811434 | COPI-dependent Golgi-to-ER retrograde traffic | 5 | 8.25E-05 |
|  | R-HSA-9646399 | Aggrephagy | 4 | 8.25E-05 |
|  | R-HSA-9648025 | EML4 and NUDC in mitotic spindle formation | 5 | 1.33E-04 |
|  | R-HSA-6811442 | Intra-Golgi and retrograde Golgi-to-ER traffic | 6 | 1.42E-04 |
|  | R-HSA-2500257 | Resolution of Sister Chromatid Cohesion | 5 | 1.83E-04 |
|  | R-HSA-6811436 | COPI-independent Golgi-to-ER retrograde traffic | 4 | 1.83E-04 |
|  | R-HSA-190840 | Microtubule-dependent trafficking of connexons from Golgi to the plasma membrane | 3 | 2.37E-04 |
|  | R-HSA-190872 | Transport of connexons to the plasma membrane | 3 | 2.50E-04 |
|  | R-HSA-389977 | Post-chaperonin tubulin folding pathway | 3 | 3.20E-04 |
|  | R-HSA-1445148 | Translocation of SLC2A4 (GLUT4) to the plasma membrane | 4 | 3.46E-04 |
|  | R-HSA-3858494 | Beta-catenin independent WNT signaling | 5 | 3.88E-04 |
|  | R-HSA-389960 | Formation of tubulin folding intermediates by CCT/TriC | 3 | 4.57E-04 |
|  | R-HSA-9663891 | Selective autophagy | 4 | 4.98E-04 |
|  | R-HSA-162582 | Signal Transduction | 17 | 5.94E-04 |
|  | R-HSA-2467813 | Separation of Sister Chromatids | 5 | 5.94E-04 |
|  | R-HSA-438064 | Post NMDA receptor activation events | 4 | 5.94E-04 |
|  | R-HSA-380320 | Recruitment of NuMA to mitotic centrosomes | 4 | 5.94E-04 |
|  | R-HSA-4086400 | PCP/CE pathway | 4 | 5.94E-04 |
|  | R-HSA-9619483 | Activation of AMPK downstream of NMDARs | 3 | 5.94E-04 |
|  | R-HSA-389958 | Cooperation of Prefoldin and TriC/CCT in actin and tubulin folding | 3 | 5.94E-04 |
|  | R-HSA-199991 | Membrane Trafficking | 8 | 6.07E-04 |
|  | R-HSA-195721 | Signaling by WNT | 6 | 6.07E-04 |
|  | R-HSA-6807878 | COPI-mediated anterograde transport | 4 | 6.07E-04 |
|  | R-HSA-9668328 | Sealing of the nuclear envelope (NE) by ESCRT-III | 3 | 6.07E-04 |
|  | R-HSA-68877 | Mitotic Prometaphase | 5 | 6.53E-04 |
|  | R-HSA-190861 | Gap junction assembly | 3 | 6.89E-04 |
|  | R-HSA-442755 | Activation of NMDA receptors and postsynaptic events | 4 | 7.48E-04 |
|  | R-HSA-5610787 | Hedgehog 'off' state | 4 | 9.68E-04 |
|  | R-HSA-9609736 | Assembly and cell surface presentation of NMDA receptors | 3 | 9.68E-04 |
|  | R-HSA-2262752 | Cellular responses to stress | 9 | 9.72E-04 |
|  | R-HSA-373760 | L1CAM interactions | 4 | 1.06E-03 |
|  | R-HSA-8953897 | Cellular responses to stimuli | 9 | 1.08E-03 |
|  | R-HSA-68882 | Mitotic Anaphase | 5 | 1.08E-03 |
|  | R-HSA-2555396 | Mitotic Metaphase and Anaphase | 5 | 1.08E-03 |
|  | R-HSA-3371556 | Cellular response to heat stress | 4 | 1.08E-03 |
|  | R-HSA-190828 | Gap junction trafficking | 3 | 1.08E-03 |
|  | R-HSA-8955332 | Carboxyterminal post-translational modifications of tubulin | 3 | 1.08E-03 |
|  | R-HSA-437239 | Recycling pathway of L1 | 3 | 1.08E-03 |
|  | R-HSA-5620924 | Intraflagellar transport | 3 | 1.14E-03 |
|  | R-HSA-157858 | Gap junction trafficking and regulation | 3 | 1.14E-03 |
|  | R-HSA-422475 | Axon guidance | 7 | 1.14E-03 |
|  | R-HSA-1632852 | Macroautophagy | 4 | 1.46E-03 |
|  | R-HSA-5653656 | Vesicle-mediated transport | 8 | 1.64E-03 |
|  | R-HSA-9675108 | Nervous system development | 7 | 1.64E-03 |
|  | R-HSA-9012999 | RHO GTPase cycle | 6 | 1.67E-03 |
|  | R-HSA-199977 | ER to Golgi Anterograde Transport | 4 | 1.67E-03 |
|  | R-HSA-9612973 | Autophagy | 4 | 1.67E-03 |
|  | R-HSA-5358351 | Signaling by Hedgehog | 4 | 1.67E-03 |
|  | R-HSA-416550 | Sema4D mediated inhibition of cell attachment and migration | 2 | 1.67E-03 |
|  | R-HSA-399955 | SEMA3A-Plexin repulsion signaling by inhibiting Integrin adhesion | 2 | 2.63E-03 |
|  | R-HSA-8852276 | The role of GTSE1 in G2/M progression after G2 checkpoint | 3 | 2.68E-03 |
|  | R-HSA-2995410 | Nuclear Envelope (NE) Reassembly | 3 | 3.17E-03 |
|  | R-HSA-5617833 | Cilium Assembly | 4 | 3.69E-03 |
|  | R-HSA-69275 | G2/M Transition | 4 | 3.96E-03 |
|  | R-HSA-390466 | Chaperonin-mediated protein folding | 3 | 4.06E-03 |
|  | R-HSA-453274 | Mitotic G2-G2/M phases | 4 | 4.09E-03 |
|  | R-HSA-9609690 | HCMV Early Events | 4 | 4.16E-03 |
|  | R-HSA-373080 | Class B/2 (Secretin family receptors) | 3 | 4.43E-03 |
|  | R-HSA-948021 | Transport to the Golgi and subsequent modification | 4 | 4.45E-03 |
|  | R-HSA-1266738 | Developmental Biology | 9 | 4.69E-03 |
|  | R-HSA-391251 | Protein folding | 3 | 4.83E-03 |
| 16 | R-HSA-112316 | Neuronal System | 8 | 3.58E-03 |
|  | R-HSA-112315 | Transmission across Chemical Synapses | 7 | 3.58E-03 |
|  | R-HSA-6794361 | Neurexins and neuroligins | 4 | 3.58E-03 |
|  | R-HSA-1236394 | Signaling by ERBB4 | 4 | 5.09E-03 |
|  | R-HSA-9013694 | Signaling by NOTCH4 | 4 | 5.09E-03 |
|  | R-HSA-6794362 | Protein-protein interactions at synapses | 4 | 5.09E-03 |
|  | R-HSA-450531 | Regulation of mRNA stability by proteins that bind AU-rich elements | 4 | 5.09E-03 |
|  | R-HSA-8941333 | RUNX2 regulates genes involved in differentiation of myeloid cells | 2 | 5.10E-03 |
|  | R-HSA-442755 | Activation of NMDA receptors and postsynaptic events | 4 | 8.28E-03 |
|  | R-HSA-1251985 | Nuclear signaling by ERBB4 | 3 | 8.90E-03 |
|  | R-HSA-9609736 | Assembly and cell surface presentation of NMDA receptors | 3 | 9.16E-03 |
|  | R-HSA-8854050 | FBXL7 down-regulates AURKA during mitotic entry and in early mitosis | 3 | 9.39E-03 |
|  | R-HSA-1483191 | Synthesis of PC | 3 | 9.39E-03 |
|  | R-HSA-450408 | AUF1 (hnRNP D0) binds and destabilizes mRNA | 3 | 9.39E-03 |
|  | R-HSA-9604323 | Negative regulation of NOTCH4 signaling | 3 | 9.39E-03 |
|  | R-HSA-8939246 | RUNX1 regulates transcription of genes involved in differentiation of myeloid cells | 2 | 9.39E-03 |
|  | R-HSA-9013700 | NOTCH4 Activation and Transmission of Signal to the Nucleus | 2 | 9.39E-03 |
|  | R-HSA-8878171 | Transcriptional regulation by RUNX1 | 5 | 9.96E-03 |
|  | R-HSA-8878166 | Transcriptional regulation by RUNX2 | 4 | 9.96E-03 |
|  | R-HSA-8932339 | ROS sensing by NFE2L2 | 3 | 9.96E-03 |
|  | R-HSA-8948751 | Regulation of PTEN stability and activity | 3 | 1.52E-02 |
|  | R-HSA-9707587 | Regulation of HMOX1 expression and activity | 3 | 1.52E-02 |
|  | R-HSA-983168 | Antigen processing: Ubiquitination & Proteasome degradation | 5 | 1.85E-02 |
|  | R-HSA-6798695 | Neutrophil degranulation | 6 | 2.01E-02 |
|  | R-HSA-109581 | Apoptosis | 4 | 2.03E-02 |
|  | R-HSA-977225 | Amyloid fiber formation | 3 | 2.03E-02 |
|  | R-HSA-8849932 | Synaptic adhesion-like molecules | 2 | 2.03E-02 |
|  | R-HSA-69275 | G2/M Transition | 4 | 2.24E-02 |
|  | R-HSA-453274 | Mitotic G2-G2/M phases | 4 | 2.24E-02 |
|  | R-HSA-201681 | TCF dependent signaling in response to WNT | 4 | 2.24E-02 |
|  | R-HSA-438064 | Post NMDA receptor activation events | 3 | 2.24E-02 |
|  | R-HSA-422085 | Synthesis, secretion, and deacylation of Ghrelin | 2 | 2.24E-02 |
|  | R-HSA-438066 | Unblocking of NMDA receptors, glutamate binding and activation | 2 | 2.24E-02 |
|  | R-HSA-9617324 | Negative regulation of NMDA receptor-mediated neuronal transmission | 2 | 2.24E-02 |
|  | R-HSA-212676 | Dopamine Neurotransmitter Release Cycle | 2 | 2.24E-02 |
|  | R-HSA-5687128 | MAPK6/MAPK4 signaling | 3 | 2.43E-02 |
|  | R-HSA-392499 | Metabolism of proteins | 13 | 2.44E-02 |
|  | R-HSA-112314 | Neurotransmitter receptors and postsynaptic signal transmission | 4 | 2.44E-02 |
|  | R-HSA-9620244 | Long-term potentiation | 2 | 2.44E-02 |
|  | R-HSA-9022699 | MECP2 regulates neuronal receptors and channels | 2 | 2.58E-02 |
|  | R-HSA-5357801 | Programmed Cell Death | 4 | 2.72E-02 |
|  | R-HSA-8951664 | Neddylation | 4 | 2.84E-02 |
|  | R-HSA-112311 | Neurotransmitter clearance | 2 | 2.91E-02 |
|  | R-HSA-157118 | Signaling by NOTCH | 4 | 3.16E-02 |
|  | R-HSA-212436 | Generic Transcription Pathway | 10 | 3.85E-02 |
|  | R-HSA-73857 | RNA Polymerase II Transcription | 10 | 4.00E-02 |
|  | R-HSA-74160 | Gene expression (Transcription) | 10 | 4.00E-02 |
|  | R-HSA-1640170 | Cell Cycle | 6 | 4.00E-02 |
|  | R-HSA-983169 | Class I MHC mediated antigen processing & presentation | 5 | 4.00E-02 |
|  | R-HSA-422475 | Axon guidance | 5 | 4.00E-02 |
|  | R-HSA-69278 | Cell Cycle, Mitotic | 5 | 4.00E-02 |
|  | R-HSA-69620 | Cell Cycle Checkpoints | 4 | 4.00E-02 |
|  | R-HSA-195721 | Signaling by WNT | 4 | 4.00E-02 |
|  | R-HSA-5683057 | MAPK family signaling cascades | 4 | 4.00E-02 |
|  | R-HSA-69481 | G2/M Checkpoints | 3 | 4.00E-02 |
|  | R-HSA-9707564 | Cytoprotection by HMOX1 | 3 | 4.00E-02 |
|  | R-HSA-6807070 | PTEN Regulation | 3 | 4.00E-02 |
|  | R-HSA-2467813 | Separation of Sister Chromatids | 3 | 4.00E-02 |
|  | R-HSA-9711123 | Cellular response to chemical stress | 3 | 4.00E-02 |
|  | R-HSA-211733 | Regulation of activated PAK-2p34 by proteasome mediated degradation | 2 | 4.00E-02 |
|  | R-HSA-350562 | Regulation of ornithine decarboxylase (ODC) | 2 | 4.00E-02 |
|  | R-HSA-8864260 | Transcriptional regulation by the AP-2 (TFAP2) family of transcription factors | 2 | 4.00E-02 |
|  | R-HSA-180534 | Vpu mediated degradation of CD4 | 2 | 4.00E-02 |
|  | R-HSA-1236978 | Cross-presentation of soluble exogenous antigens (endosomes) | 2 | 4.00E-02 |
|  | R-HSA-69613 | p53-Independent G1/S DNA damage checkpoint | 2 | 4.00E-02 |
|  | R-HSA-69601 | Ubiquitin Mediated Degradation of Phosphorylated Cdc25A | 2 | 4.00E-02 |
|  | R-HSA-69610 | p53-Independent DNA Damage Response | 2 | 4.00E-02 |
|  | R-HSA-349425 | Autodegradation of the E3 ubiquitin ligase COP1 | 2 | 4.00E-02 |
|  | R-HSA-75815 | Ubiquitin-dependent degradation of Cyclin D | 2 | 4.00E-02 |
|  | R-HSA-169911 | Regulation of Apoptosis | 2 | 4.00E-02 |
|  | R-HSA-174113 | SCF-beta-TrCP mediated degradation of Emi1 | 2 | 4.00E-02 |
|  | R-HSA-180585 | Vif-mediated degradation of APOBEC3G | 2 | 4.00E-02 |
|  | R-HSA-4641258 | Degradation of DVL | 2 | 4.00E-02 |
|  | R-HSA-4641257 | Degradation of AXIN | 2 | 4.00E-02 |
|  | R-HSA-8941858 | Regulation of RUNX3 expression and activity | 2 | 4.00E-02 |
|  | R-HSA-68827 | CDT1 association with the CDC6:ORC:origin complex | 2 | 4.00E-02 |
|  | R-HSA-69541 | Stabilization of p53 | 2 | 4.00E-02 |
|  | R-HSA-5362768 | Hh mutants are degraded by ERAD | 2 | 4.00E-02 |
|  | R-HSA-5676590 | NIK-->noncanonical NF-kB signaling | 2 | 4.00E-02 |
|  | R-HSA-5610780 | Degradation of GLI1 by the proteasome | 2 | 4.00E-02 |
|  | R-HSA-5610785 | GLI3 is processed to GLI3R by the proteasome | 2 | 4.00E-02 |
|  | R-HSA-5610783 | Degradation of GLI2 by the proteasome | 2 | 4.00E-02 |
|  | R-HSA-187577 | SCF(Skp2)-mediated degradation of p27/p21 | 2 | 4.00E-02 |
|  | R-HSA-174084 | Autodegradation of Cdh1 by Cdh1:APC/C | 2 | 4.00E-02 |
|  | R-HSA-5387390 | Hh mutants abrogate ligand secretion | 2 | 4.00E-02 |
|  | R-HSA-5678895 | Defective CFTR causes cystic fibrosis | 2 | 4.00E-02 |
|  | R-HSA-4608870 | Asymmetric localization of PCP proteins | 2 | 4.00E-02 |
|  | R-HSA-5607761 | Dectin-1 mediated noncanonical NF-kB signaling | 2 | 4.00E-02 |
|  | R-HSA-9029569 | NR1H3 & NR1H2 regulate gene expression linked to cholesterol transport and efflux | 2 | 4.00E-02 |
|  | R-HSA-174154 | APC/C:Cdc20 mediated degradation of Securin | 2 | 4.00E-02 |
|  | R-HSA-68867 | Assembly of the pre-replicative complex | 2 | 4.00E-02 |
|  | R-HSA-69580 | p53-Dependent G1/S DNA damage checkpoint | 2 | 4.00E-02 |
|  | R-HSA-69563 | p53-Dependent G1 DNA Damage Response | 2 | 4.00E-02 |
|  | R-HSA-5658442 | Regulation of RAS by GAPs | 2 | 4.00E-02 |
|  | R-HSA-1169091 | Activation of NF-kappaB in B cells | 2 | 4.00E-02 |
|  | R-HSA-1234176 | Oxygen-dependent proline hydroxylation of Hypoxia-inducible Factor Alpha | 2 | 4.00E-02 |
|  | R-HSA-69615 | G1/S DNA Damage Checkpoints | 2 | 4.00E-02 |
|  | R-HSA-5358346 | Hedgehog ligand biogenesis | 2 | 4.00E-02 |
|  | R-HSA-174184 | Cdc20:Phospho-APC/C mediated degradation of Cyclin A | 2 | 4.00E-02 |
|  | R-HSA-68949 | Orc1 removal from chromatin | 2 | 4.00E-02 |
|  | R-HSA-174178 | APC/C:Cdh1 mediated degradation of Cdc20 and other APC/C:Cdh1 targeted proteins in late mitosis/early G1 | 2 | 4.00E-02 |
|  | R-HSA-179419 | APC:Cdc20 mediated degradation of cell cycle proteins prior to satisfation of the cell cycle checkpoint | 2 | 4.00E-02 |
|  | R-HSA-69017 | CDK-mediated phosphorylation and removal of Cdc6 | 2 | 4.00E-02 |
|  | R-HSA-9662360 | Sensory processing of sound by inner hair cells of the cochlea | 2 | 4.00E-02 |
|  | R-HSA-176409 | APC/C:Cdc20 mediated degradation of mitotic proteins | 2 | 4.00E-02 |
|  | R-HSA-176814 | Activation of APC/C and APC/C:Cdc20 mediated degradation of mitotic proteins | 2 | 4.00E-02 |
|  | R-HSA-351202 | Metabolism of polyamines | 2 | 4.00E-02 |
|  | R-HSA-8939902 | Regulation of RUNX2 expression and activity | 2 | 4.00E-02 |
|  | R-HSA-8852276 | The role of GTSE1 in G2/M progression after G2 checkpoint | 2 | 4.00E-02 |
|  | R-HSA-176408 | Regulation of APC/C activators between G1/S and early anaphase | 2 | 4.00E-02 |
|  | R-HSA-1483206 | Glycerophospholipid biosynthesis | 3 | 4.03E-02 |
|  | R-HSA-9024446 | NR1H2 and NR1H3-mediated signaling | 2 | 4.18E-02 |
|  | R-HSA-1234174 | Cellular response to hypoxia | 2 | 4.27E-02 |
|  | R-HSA-9006934 | Signaling by Receptor Tyrosine Kinases | 5 | 4.30E-02 |
|  | R-HSA-9659379 | Sensory processing of sound | 2 | 4.36E-02 |
|  | R-HSA-9675108 | Nervous system development | 5 | 4.38E-02 |
|  | R-HSA-69202 | Cyclin E associated events during G1/S transition | 2 | 4.46E-02 |
|  | R-HSA-69002 | DNA Replication Pre-Initiation | 2 | 4.46E-02 |
|  | R-HSA-68886 | M Phase | 4 | 4.56E-02 |
|  | R-HSA-69656 | Cyclin A:Cdk2-associated events at S phase entry | 2 | 4.65E-02 |
|  | R-HSA-1280218 | Adaptive Immune System | 6 | 4.73E-02 |
|  | R-HSA-68882 | Mitotic Anaphase | 3 | 4.73E-02 |
|  | R-HSA-2555396 | Mitotic Metaphase and Anaphase | 3 | 4.73E-02 |
|  | R-HSA-1168372 | Downstream signaling events of B Cell Receptor (BCR) | 2 | 4.73E-02 |
|  | R-HSA-195253 | Degradation of beta-catenin by the destruction complex | 2 | 4.73E-02 |
|  | R-HSA-453276 | Regulation of mitotic cell cycle | 2 | 4.73E-02 |
|  | R-HSA-174143 | APC/C-mediated degradation of cell cycle proteins | 2 | 4.73E-02 |
|  | R-HSA-5632684 | Hedgehog 'on' state | 2 | 4.73E-02 |
|  | R-HSA-69052 | Switching of origins to a post-replicative state | 2 | 4.73E-02 |
|  | R-HSA-5689603 | UCH proteinases | 2 | 4.73E-02 |
|  | R-HSA-112310 | Neurotransmitter release cycle | 2 | 4.73E-02 |
|  | R-HSA-5619084 | ABC transporter disorders | 2 | 4.73E-02 |
|  | R-HSA-4086400 | PCP/CE pathway | 2 | 4.73E-02 |
|  | R-HSA-8986944 | Transcriptional Regulation by MECP2 | 2 | 4.73E-02 |
|  | R-HSA-5668541 | TNFR2 non-canonical NF-kB pathway | 2 | 4.73E-02 |
|  | R-HSA-8939236 | RUNX1 regulates transcription of genes involved in differentiation of HSCs | 2 | 4.73E-02 |
|  | R-HSA-8957275 | Post-translational protein phosphorylation | 2 | 4.73E-02 |
|  | R-HSA-9020702 | Interleukin-1 signaling | 2 | 4.73E-02 |
|  | R-HSA-8878159 | Transcriptional regulation by RUNX3 | 2 | 4.73E-02 |
|  | R-HSA-5607764 | CLEC7A (Dectin-1) signaling | 2 | 4.73E-02 |
|  | R-HSA-382556 | ABC-family proteins mediated transport | 2 | 4.73E-02 |
|  | R-HSA-5610787 | Hedgehog 'off' state | 2 | 4.73E-02 |
|  | R-HSA-202424 | Downstream TCR signaling | 2 | 4.73E-02 |
|  | R-HSA-381426 | Regulation of Insulin-like Growth Factor (IGF) transport and uptake by Insulin-like Growth Factor Binding Proteins (IGFBPs) | 2 | 4.73E-02 |
|  | R-HSA-2980736 | Peptide hormone metabolism | 2 | 4.73E-02 |
|  | R-HSA-373760 | L1CAM interactions | 2 | 4.73E-02 |
|  | R-HSA-69239 | Synthesis of DNA | 2 | 4.73E-02 |
|  | R-HSA-1483257 | Phospholipid metabolism | 3 | 4.89E-02 |
| 17 | R-HSA-6809371 | Formation of the cornified envelope | 12 | 5.77E-15 |
|  | R-HSA-6805567 | Keratinization | 12 | 1.15E-14 |
|  | R-HSA-1266738 | Developmental Biology | 14 | 1.97E-08 |
|  | R-HSA-6785807 | Interleukin-4 and Interleukin-13 signaling | 6 | 1.30E-05 |
|  | R-HSA-449147 | Signaling by Interleukins | 6 | 4.51E-03 |
|  | R-HSA-9702518 | STAT5 activation downstream of FLT3 ITD mutants | 2 | 4.51E-03 |
|  | R-HSA-9703648 | Signaling by FLT3 ITD and TKD mutants | 2 | 7.76E-03 |
|  | R-HSA-9703465 | Signaling by FLT3 fusion proteins | 2 | 7.76E-03 |
|  | R-HSA-9682385 | FLT3 signaling in disease | 2 | 1.16E-02 |
|  | R-HSA-452723 | Transcriptional regulation of pluripotent stem cells | 2 | 1.26E-02 |
|  | R-HSA-1280215 | Cytokine Signaling in Immune system | 6 | 2.96E-02 |
| 20 | R-HSA-8935964 | RUNX1 regulates expression of components of tight junctions | 3 | 7.77E-05 |
|  | R-HSA-8939242 | RUNX1 regulates transcription of genes involved in differentiation of keratinocytes | 3 | 1.01E-04 |
|  | R-HSA-8878171 | Transcriptional regulation by RUNX1 | 6 | 4.30E-04 |
|  | R-HSA-8951911 | RUNX3 regulates RUNX1-mediated transcription | 2 | 1.02E-03 |
|  | R-HSA-1679131 | Trafficking and processing of endosomal TLR | 2 | 1.26E-02 |
|  | R-HSA-8878159 | Transcriptional regulation by RUNX3 | 3 | 2.79E-02 |
|  | R-HSA-6803529 | FGFR2 alternative splicing | 2 | 2.79E-02 |
|  | R-HSA-420029 | Tight junction interactions | 2 | 2.79E-02 |
|  | R-HSA-8934593 | Regulation of RUNX1 Expression and Activity | 2 | 2.82E-02 |
| 21 | R-HSA-6809371 | Formation of the cornified envelope | 5 | 2.37E-05 |
|  | R-HSA-1839130 | Signaling by activated point mutants of FGFR3 | 3 | 2.62E-05 |
|  | R-HSA-2033514 | FGFR3 mutant receptor activation | 3 | 2.62E-05 |
|  | R-HSA-6805567 | Keratinization | 5 | 6.52E-05 |
|  | R-HSA-5655332 | Signaling by FGFR3 in disease | 3 | 7.68E-05 |
|  | R-HSA-8853338 | Signaling by FGFR3 point mutants in cancer | 3 | 7.68E-05 |
|  | R-HSA-2428928 | IRS-related events triggered by IGF1R | 3 | 4.77E-04 |
|  | R-HSA-2428924 | IGF1R signaling cascade | 3 | 4.77E-04 |
|  | R-HSA-2404192 | Signaling by Type 1 Insulin-like Growth Factor 1 Receptor (IGF1R) | 3 | 4.77E-04 |
|  | R-HSA-351906 | Apoptotic cleavage of cell adhesion proteins | 2 | 4.77E-04 |
|  | R-HSA-1226099 | Signaling by FGFR in disease | 3 | 5.71E-04 |
|  | R-HSA-428359 | Insulin-like Growth Factor-2 mRNA Binding Proteins (IGF2BPs/IMPs/VICKZs) bind RNA | 2 | 5.71E-04 |
|  | R-HSA-5654227 | Phospholipase C-mediated cascade; FGFR3 | 2 | 9.09E-04 |
|  | R-HSA-190239 | FGFR3 ligand binding and activation | 2 | 1.01E-03 |
|  | R-HSA-5654710 | PI-3K cascade:FGFR3 | 2 | 1.51E-03 |
|  | R-HSA-5654704 | SHC-mediated cascade:FGFR3 | 2 | 1.51E-03 |
|  | R-HSA-5654706 | FRS-mediated FGFR3 signaling | 2 | 1.63E-03 |
|  | R-HSA-5654732 | Negative regulation of FGFR3 signaling | 2 | 2.40E-03 |
|  | R-HSA-5654708 | Downstream signaling of activated FGFR3 | 2 | 2.40E-03 |
|  | R-HSA-111465 | Apoptotic cleavage of cellular proteins | 2 | 2.40E-03 |
|  | R-HSA-5663202 | Diseases of signal transduction by growth factor receptors and second messengers | 4 | 4.60E-03 |
|  | R-HSA-5654741 | Signaling by FGFR3 | 2 | 4.60E-03 |
|  | R-HSA-75153 | Apoptotic execution phase | 2 | 4.60E-03 |
|  | R-HSA-109704 | PI3K Cascade | 2 | 4.60E-03 |
|  | R-HSA-112399 | IRS-mediated signaling | 2 | 4.60E-03 |
|  | R-HSA-74751 | Insulin receptor signaling cascade | 2 | 5.62E-03 |
|  | R-HSA-1266738 | Developmental Biology | 5 | 9.76E-03 |
|  | R-HSA-5673001 | RAF/MAP kinase cascade | 3 | 9.76E-03 |
|  | R-HSA-5684996 | MAPK1/MAPK3 signaling | 3 | 9.76E-03 |
|  | R-HSA-5683057 | MAPK family signaling cascades | 3 | 9.76E-03 |
|  | R-HSA-2219530 | Constitutive Signaling by Aberrant PI3K in Cancer | 2 | 9.76E-03 |
|  | R-HSA-74752 | Signaling by Insulin receptor | 2 | 9.76E-03 |
|  | R-HSA-190236 | Signaling by FGFR | 2 | 9.76E-03 |
|  | R-HSA-2219528 | PI3K/AKT Signaling in Cancer | 2 | 9.76E-03 |
|  | R-HSA-6811558 | PI5P, PP2A and IER3 Regulate PI3K/AKT Signaling | 2 | 9.76E-03 |
|  | R-HSA-199418 | Negative regulation of the PI3K/AKT network | 2 | 9.76E-03 |
|  | R-HSA-114608 | Platelet degranulation | 2 | 1.00E-02 |
|  | R-HSA-76005 | Response to elevated platelet cytosolic Ca2+ | 2 | 1.10E-02 |
|  | R-HSA-109581 | Apoptosis | 2 | 1.85E-02 |
|  | R-HSA-5357801 | Programmed Cell Death | 2 | 2.76E-02 |
|  | R-HSA-9006934 | Signaling by Receptor Tyrosine Kinases | 3 | 2.83E-02 |
|  | R-HSA-877300 | Interferon gamma signaling | 2 | 3.02E-02 |
|  | R-HSA-76002 | Platelet activation, signaling and aggregation | 2 | 3.99E-02 |
|  | R-HSA-1257604 | PIP3 activates AKT signaling | 2 | 4.71E-02 |

FDR, false discovery rate.

**Supplementary Table 4.** Biological processes significantly deregulated in primary ameloblastoma compared with the normal gingiva.

| **Cluster No.** | **GO ID** | **GO Term** | **# Entities** | **FDR** |
| --- | --- | --- | --- | --- |
| 1 | GO:0006260 | DNA replication | 16 | 4.20E-17 |
|  | GO:0007067 | mitotic nuclear division | 16 | 2.60E-14 |
|  | GO:0007062 | sister chromatid cohesion | 11 | 2.90E-11 |
|  | GO:0051301 | cell division | 15 | 4.80E-11 |
|  | GO:0007059 | chromosome segregation | 9 | 1.00E-09 |
|  | GO:0000082 | G1/S transition of mitotic cell cycle | 9 | 2.40E-08 |
|  | GO:0000086 | G2/M transition of mitotic cell cycle | 8 | 5.50E-06 |
|  | GO:0006281 | DNA repair | 9 | 1.30E-05 |
|  | GO:0051726 | regulation of cell cycle | 7 | 5.10E-05 |
|  | GO:0000083 | regulation of transcription involved in G1/S transition of mitotic cell cycle | 4 | 1.20E-03 |
|  | GO:0000731 | DNA synthesis involved in DNA repair | 4 | 4.10E-03 |
|  | GO:0034080 | CENP-A containing nucleosome assembly | 4 | 6.90E-03 |
|  | GO:0006974 | cellular response to DNA damage stimulus | 6 | 7.40E-03 |
|  | GO:0016925 | protein sumoylation | 5 | 7.40E-03 |
|  | GO:0006268 | DNA unwinding involved in DNA replication | 3 | 7.40E-03 |
|  | GO:0034501 | protein localization to kinetochore | 3 | 7.40E-03 |
|  | GO:0051382 | kinetochore assembly | 3 | 1.00E-02 |
|  | GO:0008283 | cell proliferation | 7 | 1.10E-02 |
|  | GO:0006302 | double-strand break repair | 4 | 1.60E-02 |
|  | GO:0007051 | spindle organization | 3 | 1.60E-02 |
|  | GO:0007088 | regulation of mitotic nuclear division | 3 | 1.60E-02 |
|  | GO:0000070 | mitotic sister chromatid segregation | 3 | 3.40E-02 |
|  | GO:0000732 | strand displacement | 3 | 3.50E-02 |
|  | GO:0000281 | mitotic cytokinesis | 3 | 3.60E-02 |
|  | GO:0007131 | reciprocal meiotic recombination | 3 | 4.30E-02 |
|  | GO:0007049 | cell cycle | 5 | 4.30E-02 |
|  | GO:0006335 | DNA replication-dependent nucleosome assembly | 3 | 4.30E-02 |
|  | GO:0006270 | DNA replication initiation | 3 | 4.50E-02 |
| 2 | GO:0030574 | collagen catabolic process | 11 | 3.50E-13 |
|  | GO:0030198 | extracellular matrix organization | 13 | 1.90E-11 |
|  | GO:0006096 | glycolytic process | 6 | 3.30E-06 |
|  | GO:0061621 | canonical glycolysis | 5 | 5.40E-05 |
|  | GO:0030199 | collagen fibril organization | 5 | 2.30E-04 |
|  | GO:0070208 | protein heterotrimerization | 4 | 3.50E-04 |
|  | GO:0071230 | cellular response to amino acid stimulus | 5 | 3.50E-04 |
|  | GO:0006123 | mitochondrial electron transport, cytochrome c to oxygen | 4 | 8.20E-04 |
|  | GO:0042776 | mitochondrial ATP synthesis coupled proton transport | 4 | 8.50E-04 |
|  | GO:0006099 | tricarboxylic acid cycle | 4 | 2.10E-03 |
|  | GO:0001649 | osteoblast differentiation | 5 | 5.20E-03 |
|  | GO:0006094 | gluconeogenesis | 4 | 6.00E-03 |
|  | GO:0006120 | mitochondrial electron transport, NADH to ubiquinone | 4 | 7.70E-03 |
|  | GO:0006098 | pentose-phosphate shunt | 3 | 8.70E-03 |
|  | GO:0001501 | skeletal system development | 5 | 1.10E-02 |
|  | GO:0032981 | mitochondrial respiratory chain complex I assembly | 4 | 1.30E-02 |
|  | GO:0006122 | mitochondrial electron transport, ubiquinol to cytochrome c | 3 | 1.40E-02 |
|  | GO:0055093 | response to hyperoxia | 3 | 1.70E-02 |
|  | GO:0006103 | 2-oxoglutarate metabolic process | 3 | 1.80E-02 |
|  | GO:0007155 | cell adhesion | 7 | 2.30E-02 |
|  | GO:0001958 | endochondral ossification | 3 | 3.40E-02 |
|  | GO:0035987 | endodermal cell differentiation | 3 | 3.50E-02 |
|  | GO:0006754 | ATP biosynthetic process | 3 | 3.80E-02 |
| 3 | GO:0006695 | cholesterol biosynthetic process | 14 | 1.30E-24 |
|  | GO:0030198 | extracellular matrix organization | 15 | 1.10E-16 |
|  | GO:0008299 | isoprenoid biosynthetic process | 6 | 4.20E-09 |
|  | GO:0055114 | oxidation-reduction process | 11 | 1.30E-05 |
|  | GO:0006694 | steroid biosynthetic process | 4 | 1.70E-03 |
|  | GO:0022617 | extracellular matrix disassembly | 4 | 1.90E-02 |
|  | GO:0007160 | cell-matrix adhesion | 4 | 2.70E-02 |
| 5 | GO:0006099 | tricarboxylic acid cycle | 3 | 6.00E-03 |
|  | GO:0006094 | gluconeogenesis | 3 | 6.90E-03 |
| 7 | GO:0046513 | ceramide biosynthetic process | 5 | 6.20E-06 |
|  | GO:0030148 | sphingolipid biosynthetic process | 5 | 4.70E-05 |
|  | GO:0098609 | cell-cell adhesion | 5 | 2.20E-02 |
|  | GO:0030206 | chondroitin sulfate biosynthetic process | 3 | 2.20E-02 |
| 8 | GO:0014066 | regulation of phosphatidylinositol 3-kinase signaling | 5 | 1.40E-05 |
|  | GO:0046854 | phosphatidylinositol phosphorylation | 5 | 1.50E-05 |
|  | GO:0048015 | phosphatidylinositol-mediated signaling | 5 | 1.60E-05 |
|  | GO:0016337 | single organismal cell-cell adhesion | 4 | 9.70E-04 |
|  | GO:0036092 | phosphatidylinositol-3-phosphate biosynthetic process | 3 | 1.10E-02 |
|  | GO:0000165 | MAPK cascade | 4 | 1.10E-02 |
|  | GO:0014068 | positive regulation of phosphatidylinositol 3-kinase signaling | 3 | 1.50E-02 |
|  | GO:0042060 | wound healing | 3 | 1.90E-02 |
|  | GO:0002159 | desmosome assembly | 2 | 4.20E-02 |
|  | GO:0002934 | desmosome organization | 2 | 4.70E-02 |
| 9 | GO:0032008 | positive regulation of TOR signaling | 3 | 3.40E-03 |
|  | GO:0007229 | integrin-mediated signaling pathway | 3 | 2.30E-02 |
| 14 | GO:0033540 | fatty acid beta-oxidation using acyl-CoA oxidase | 3 | 2.90E-03 |
|  | GO:0006629 | lipid metabolic process | 4 | 5.90E-03 |
|  | GO:0008203 | cholesterol metabolic process | 3 | 2.70E-02 |
| 16 | GO:0043488 | regulation of mRNA stability | 5 | 8.20E-03 |
| 21 | GO:0007156 | homophilic cell adhesion via plasma membrane adhesion molecules | 4 | 1.30E-02 |

GO, gene ontology, FDR, false discovery rate.

**Supplementary Table 5.** Cellular components significantly deregulated in primary ameloblastoma compared to the normal gingiva.

| **GO ID** | **GO Term** | **# Entities** | **FDR** |
| --- | --- | --- | --- |
| GO:0070062 | extracellular exosome | 343 | 2.70E-16 |
| GO:0031012 | extracellular matrix | 61 | 1.80E-09 |
| GO:0030057 | desmosome | 13 | 1.10E-05 |
| GO:0005578 | proteinaceous extracellular matrix | 46 | 1.30E-04 |
| GO:0005581 | collagen trimer | 23 | 2.10E-04 |
| GO:0005913 | cell-cell adherens junction | 51 | 2.60E-04 |
| GO:0005788 | endoplasmic reticulum lumen | 34 | 1.20E-03 |
| GO:0005925 | focal adhesion | 56 | 1.20E-03 |
| GO:0005604 | basement membrane | 19 | 2.00E-03 |
| GO:0005829 | cytosol | 318 | 2.20E-03 |
| GO:0005737 | cytoplasm | 472 | 5.70E-03 |
| GO:0005615 | extracellular space | 143 | 6.40E-03 |
| GO:0001533 | cornified envelope | 13 | 7.10E-03 |
| GO:0005911 | cell-cell junction | 29 | 7.10E-03 |
| GO:0043231 | intracellular membrane-bounded organelle | 69 | 7.10E-03 |
| GO:0005783 | endoplasmic reticulum | 94 | 9.40E-03 |
| GO:0043209 | myelin sheath | 26 | 1.10E-02 |
| GO:0000775 | chromosome centromeric region | 14 | 1.30E-02 |
| GO:0016324 | apical plasma membrane | 39 | 4.10E-02 |
| GO:0005912 | adherens junction | 12 | 4.30E-02 |

GO, gene ontology, FDR, false discovery rate.
